# The Pictet-Spengler Reaction Updates Its Habits

**DOI:** 10.3390/molecules25020414

**Published:** 2020-01-19

**Authors:** Andrea Calcaterra, Laura Mangiardi, Giuliano Delle Monache, Deborah Quaglio, Silvia Balducci, Simone Berardozzi, Antonia Iazzetti, Roberta Franzini, Bruno Botta, Francesca Ghirga

**Affiliations:** 1 Department of Chemistry and Technology of Drugs, “Department of Excellence 2018−2022”, Sapienza University of Rome, Piazzale Aldo Moro 5, 00185 Rome, Italy; laura.mangiardi@uniroma1.it (L.M.); giugiu44@gmail.com (G.D.M.); deborah.quaglio@uniroma1.it (D.Q.); silvia.balducci@uniroma1.it (S.B.); antonia.iazzetti@uniroma1.it (A.I.); roberta.franzini@uniroma1.it (R.F.); bruno.botta@uniroma1.it (B.B.); 2 Center for Life Nano Science@Sapienza, Istituto Italiano di Tecnologia, Viale Regina Elena 291, 00161 Rome, Italy; francesca.ghirga@iit.it; 3 Department of Chemistry and Applied Biosciences, ETH-Zürich, Vladimir-Prelog Weg 4, 8093 Zürich, Switzerland

**Keywords:** Pictet-Spengler, tetrahydroisoquinoline, THIQ, tetrahydro-β-carboline, THBC, alkaloid, total synthesis, natural products, cascade reaction, multicomponent reaction

## Abstract

The Pictet-Spengler reaction (P-S) is one of the most direct, efficient, and variable synthetic method for the construction of privileged pharmacophores such as tetrahydro-isoquinolines (THIQs), tetrahydro-β-carbolines (THBCs), and polyheterocyclic frameworks. In the *lustro* (five-year period) following its centenary birthday, the P-S reaction did not exit the stage but it came up again on limelight with new features. This review focuses on the interesting results achieved in this period (2011–2015), analyzing the versatility of this reaction. Classic P-S was reported in the total synthesis of complex alkaloids, in combination with chiral catalysts as well as for the generation of libraries of compounds in medicinal chemistry. The P-S has been used also in tandem reactions, with the sequences including ring closing metathesis, isomerization, Michael addition, and Gold- or Brønsted acid-catalyzed *N*-acyliminium cyclization. Moreover, the combination of P-S reaction with Ugi multicomponent reaction has been exploited for the construction of highly complex polycyclic architectures in few steps and high yields. The P-S reaction has also been successfully employed in solid-phase synthesis, affording products with different structures, including peptidomimetics, synthetic heterocycles, and natural compounds. Finally, the enzymatic version of P-S has been reported for biosynthesis, biotransformations, and bioconjugations.

*My chameleon soul*

*Keeps turning me around*

(A. Alexopoulos)

## 1. Introduction

Few years ago, we celebrated in a paper [1] the long-lasting presence on stage of the Pictet-Spengler (P-S) cyclization as one of the most direct, efficient, and variable synthetic method for the construction of important privileged pharmacophores, such as tetrahydroisoquinolines (THIQs), tetrahydro-β-carbolines (THBCs), and polyheterocyclic architectures embodying them.

In the Act I of this tribute [1], we put in evidence some facets of the reaction, from the link to the biogenetic pathways of natural products, mainly alkaloids, to the challenge of the stereochemical outcome. Moreover, we highlighted the modifications of parameters and constituents that were employed to improve the reaction:

1. The reactivity of either nucleophile or electrophile was increased, the first by acylation of the N_b_ nitrogen [2] and the second by the presence of electron donating groups on the aromatic system [3].
2. The reaction rate was modified by changes in the reaction conditions [4,5].
3. The stereoselectivity was influenced by condensation with chiral carbonyl derivatives [6] or by the use of internal [7] or external devices [8].
4. The enantioselectivity was improved by the intervention of a chiral catalyst [9,10].

**Scheme 1:**
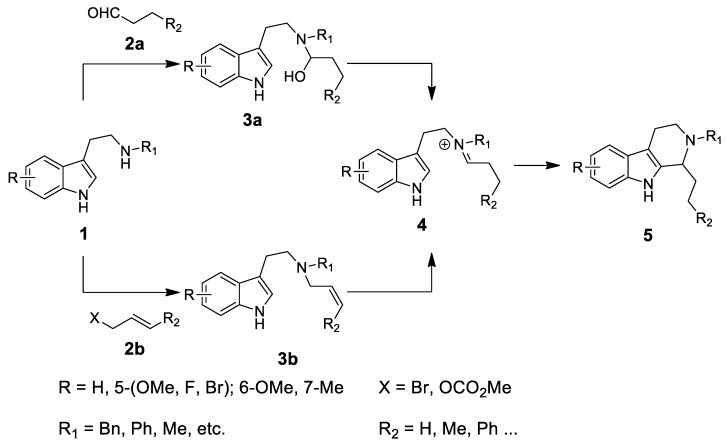
Traditional Pictet-Spengler (P-S) (up) and new metal catalyzed isomerization (down) for the synthesis of tetrahydro-β-carbolines (THBCs).

The P-S reaction essentially relies on the formation from indolylethylamine **1** and a carbonyl compound **2a** through an intermediate **3a** of the ene-iminium ion **4** tethered to an electrophilic system. The iminium ion **4**, under a nucleophilic attack, originates an annulated tetrahydropyridine moiety **5** (Scheme 1, up). Nielsen and coworkers proposed an alternative synthesis of THBCs using instead of the classical P-S addition of an active carbonyl, which is a more convenient “aldehyde free” methodology: the protagonist *N*-acyliminium ion **4** was now created by the metal-catalyzed shift of a double bond in allylic amines **3b** (Scheme 1, down). Ru(PCy_3_)(MPI)(PM)Cl_2_ (Ru alkylidene catalyst) and Rh(PPh_3_)Cl (Wilkinson’s catalyst) were identified as the most efficient catalysts by a reduction of the loading to 0.1 mol % [11].

It should be noted Gholamzadeh recently published a relevant chapter of a book, analyzing the versatility of P-S reaction in the construction of heterocyclic scaffolds [12]. Moreover, Kumar and coworkers published in 2018 a review depicting an overview on synthetic versus enzymatic P-S reaction [13].

## 2. Variations on the Classic Pictet-Spengler Reaction

**Scheme 2:**
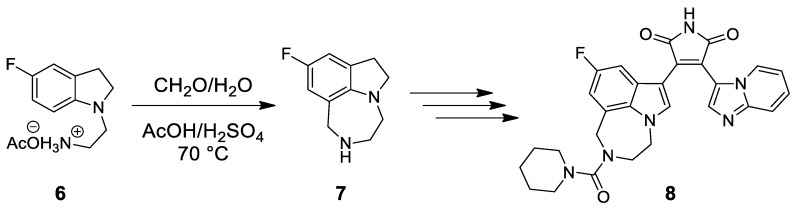
P-S based synthesis of GSK-3 inhibitor bis-arylmaleimide.

The Nielsen procedure is an example of the chameleonic versatility of P-S reaction [1]. For instance, Magnus and coworker employed the P-S methodology to access the indole C-7 position for the preparation of the benzodiazepine tricyclic fragment **7**, starting from commercially available 5-fluoroindoles **6** (Scheme 2). Compound **7** was the precursor of the synthesis of the bis-arylmaleimide **8**, a glycogen synthase kinase-3 (GSK-3) inhibitor, in 33% overall yield [14].

Subsequently, Hooker and coworkers demonstrated that this version of P-S cyclization could be used to label drugs with carbon-11 to study the pharmacokinetics of two potent agonists of the serotonin subtype 2C (5HT_2C_) receptor, namely WAY-163909 and vabicaserin. After ring-closure on C-7 by [^11^C]-formaldehyde, the two products were used in positron emission tomography (PET) imaging [15].

**Scheme 3:**
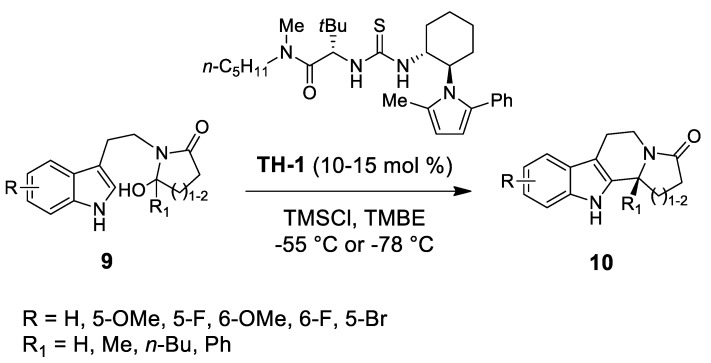
Asymmetric cyclization of hydroxylactams by thiourea catalyst **TH-1**.

The presence of additional rings in polyheterocycles containing THIQ and THBC scaffolds introduced a new complexity, which required variant or complementary reactions. For instance, the dehydrative cyclization of hydroxylactams **9** [16] (sketched in Scheme 3) is a variant that allowed for obtaining the tetracyclic quinolizinones **10**, building the extra ring prior to P-S cyclization.

**Scheme 4:**
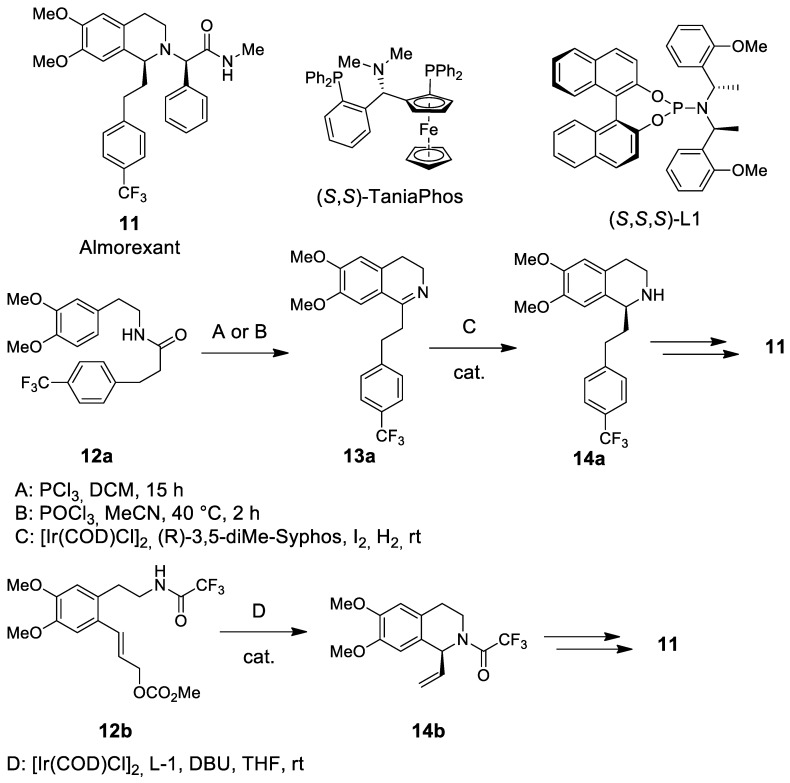
Key enantioselective steps for the synthesis of almorexant.

Two alternative routes to P-S adducts can be envisaged in the two possible pathways leading to almorexant **11**, a potent non-peptidic antagonist of human orexin receptors [17]. In a first formal synthesis (Scheme 4 up), we might combine the Bischler–Napieralsky reaction [18], which provides the 3,4-dihydroisoquinoline **13a** from arylethylamide derivative **12a** through the pathways *A* or *B*, with an asymmetric hydrogenation in presence of an iridium catalyst (pathway *C*) [19,20], which reduces the intermediate to tetrahydroisoquinoline **14a** [21]. In the second one, as reported by Feringa and coworkers, the enantioselective synthesis is achieved via iridium-catalyzed asymmetric intramolecular allylic amidation of derivative **12b** to provide the tetrahydroisoquinoline **14b** through the pathway D (Scheme 4 down) [21,22].

**Scheme 5:**
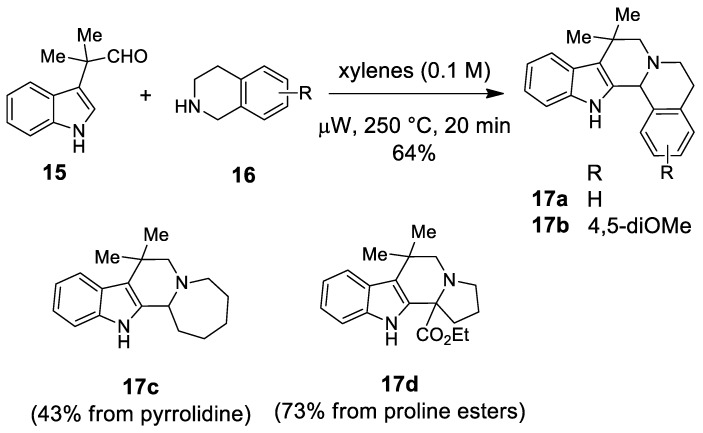
Redox annulation with concomitant formation of C-C bond.

Seidel brought to light a procedure that can be considered as a redox variant of classic P-S reaction [23]. Seidel and coworkers discovered that indole aldehyde **15** engage cyclic amines **16**, such as THIQ, pyrrolidine or proline esters, at a high temperature under microwave conditions to form the corresponding ring fused products **17** in moderate to good yields and in short times (Scheme 5). The addition of appropriate additives (e.g., carboxylic acids) and particular substrate combinations allowed for the reaction to occur at lower temperatures [24].

**Scheme 6:**
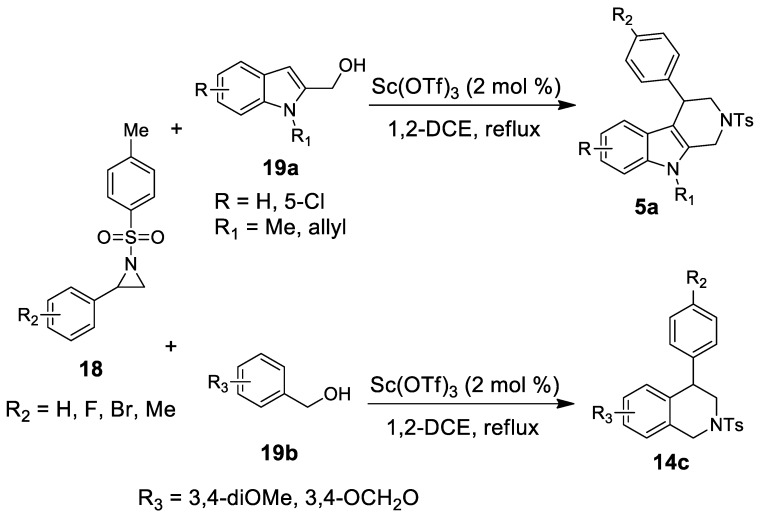
Syntheses of 4-aryl-tetrahydro-β-carbolines and 4-aryl-tetrahydroisoquinolines from aziridines and benzyl alcohols.

Finally, the novel Lewis-acid catalyzed [3 + 3]-annulation process for the synthesis of THBCs **5a** and THIQs **14c** from sulfonyl aziridines **18** and readily available benzylic alcohols **19a** and **19b** (Scheme 6), can be a valuable complement to the more widely used Pictet-Spengler condensation [25,26].

In spite of a fierce competition, the P-S cyclization maintains the role of a protagonist, enriching the chemical literature with examples of its versatility. If we take into consideration for an update the five years (2011–2015) following the one-century birthday, we can highlight that the reaction is still active in the syntheses of biologically relevant benzoannulated nitrogen heterocycles.

## 3. Update (2011–2015) of Pictet-Spengler Reactions

*I think it’s time*

*To do an update*

(Roger Turner)

### 3.1. Tetrahydroisoquinolines and Tetrahydro-β-Carbolines

Several substituted THIQ [27,28,29,30,31,32,33,34,35,36,37,38,39,40] and THBC [41,42,43,44,45,46,47,48,49,50,51,52,53,54,55,56,57,58,59,60,61,62,63,64] have been designed, synthesized via P-S cyclization, and evaluated for their biological activities. The list comprehends aminopeptidase N (APN) inhibition [27], multidrug resistance reversal effect [28], cytotoxicity against K562 [29], non-saccharide activators of antithrombin [31], anticoagulants [32], microtubule disruptors for antiproliferative activity [33,34], cytotoxicity against MOLT-3 [35] or HepG2 [36] cell lines, and the inhibitory activity towards cisplatin-insensitive cell line Skov3 [37] or the growth of *Mycobacterium tuberculosis* [39] for THIQ. For THBC, the inhibition of topoisomerase II [41], the oncogenic RAS-lethality [47], and the antimalarial activity of spirocyclic structures [44,45,46,56,60] were the most studied biological properties. 

Chiral catalysts derived from BINOL [40,44,45] and SPINOL [49] (phosphoric acids and thiourea derivatives) stand out among the usual Brønsted acids: TFA [35,42,47,57], HCl [58], 1,1,1,3,3,3-hexafluoro-2-propanol (HFIP) [59], 2,4,6-trichloro-1,3,5-triazine (TCT) [54], H_2_SO_4_ [50], MeSO_3_H [52], TsOH [58], and mild catalysts, such as phosphate buffer [30,39] and microwave irradiation [61].

**Figure 1:**
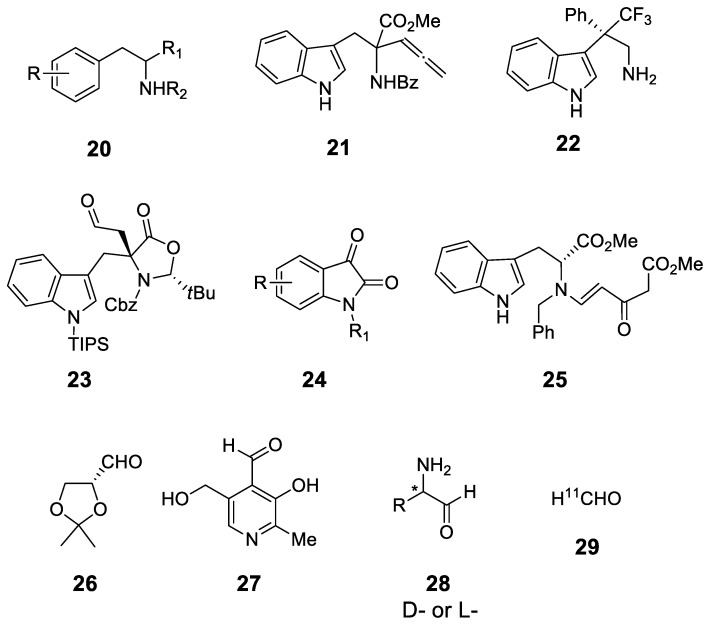
Amine (**20**–**23**) and carbonyl (**24**–**29**) substrates of P-S reactions, leading to tetrahydroisoquinolines (THIQs) and THBCs.

*N*-substituted aryl ethylamines **20** [27,31,35,36,39,40], allene-containing-tryptophan **21** [42], trifluoromethylated precursors **22** [55], and spirocyclic lactams embodying Trp **23** [56] were the most interesting amine substrates. Conversely, isatins **24** [44,45,46], enaminone **25** [52], (*S*)-2,3-*O*-isopropylidene-L-glyceraldehyde **26** [53], pyridoxal **27** [60], L/D-amino aldehydes **28** [57,63], and ^11^C-labeled formaldehyde **29** [58] were the most notable carbonyl components (Figure 1).

**Scheme 7:**
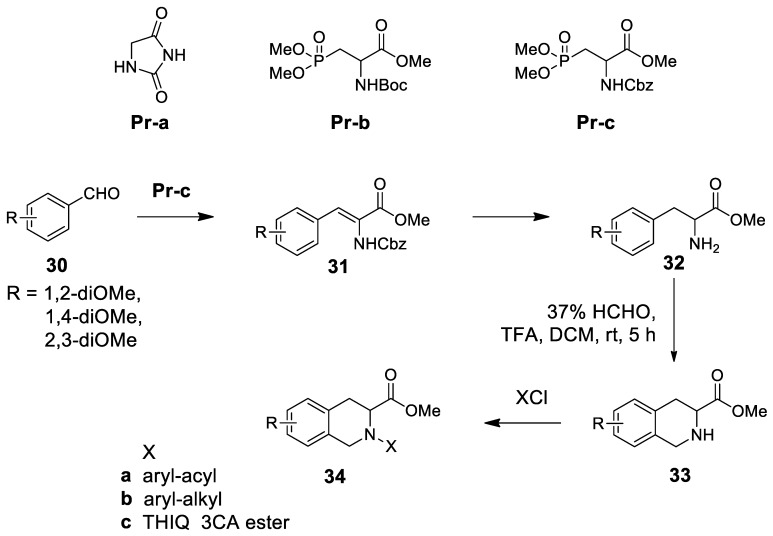
Synthesis of anticoagulants by a glycine donor strategy.

We wish to highlight few papers concerning the P-S cyclization of THIQs [32,33,34,35,36] and THBCs [38,43,51,55]. In the first one, the authors described three complementary high yielding syntheses of electronically rich 1,2,3,4-tetrahydroisoquinoline-3-carboxilic acid (THIQ3CA) esters **33**, which were based on glycine donors including hydantoin **Pr-a**, (±)-Boc-α-phosphonoglycine trimethyl ester **Pr-b**, and (±)-Z-α-phosphonoglycine trimethyl ester **Pr-c** (Scheme 7). Following this approach, a focused library of *N*-arylacyl, *N*-arylalkyl, and bis-THIQ3CA analogs **34** was synthesized [32].

**Scheme 8:**
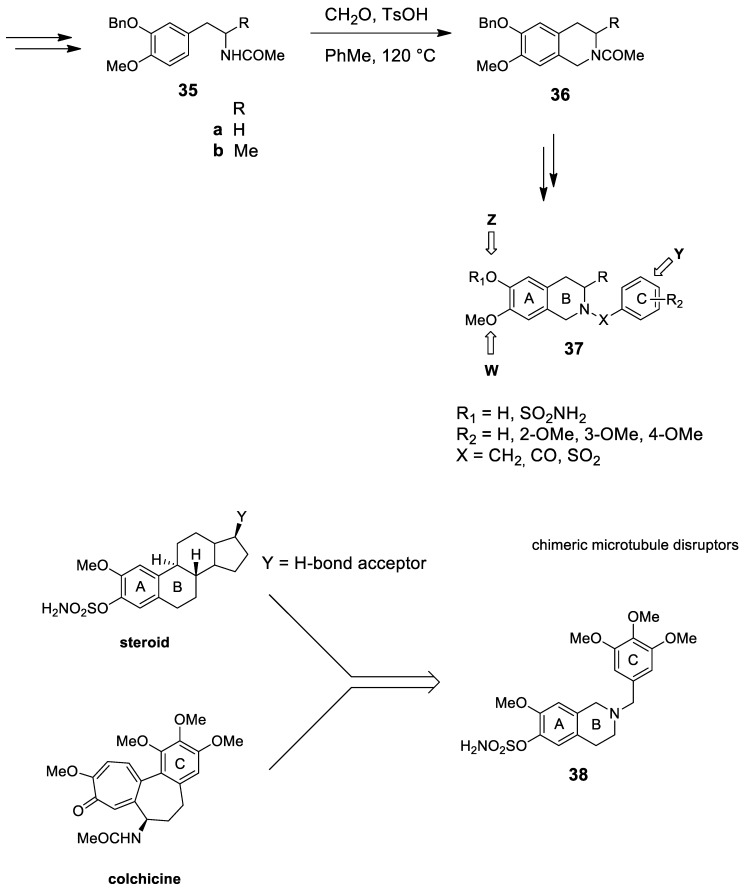
Steroidomimetic and chimeric scaffolds embodying THIQs.

Potter and coworkers developed a new series of anticancer agents by translating the key elements of steroidal pharmacophores into alternate scaffolds. The authors synthesized 6-benzyloxy-7-methoxy tetrahydroisoquinoline **36** via the key P-S reaction (Scheme 8, up), and then connected the steroid A/B ring mimicking THIQ core to monomethoxybenzyl ring through methylene [33,34], carbonyl [34], and sulfonyl linkers X [34], to provide the desired putative steroidomimetic **37**. Finally, the optimization of the representative **37** trough conformational biasing delivered a new series of microtubule disruptors with a 10-fold gain in antiproliferative activity [33]. Linkage of the THIQ-based A/B-mimic **36** with the trimethoxybenzyl motif that is prevalent in colchicine disclosed a series of chimeric molecules **38** (Scheme 8, down), whose activities surpass those of parent steroid derivatives [34].

**Scheme 9:**
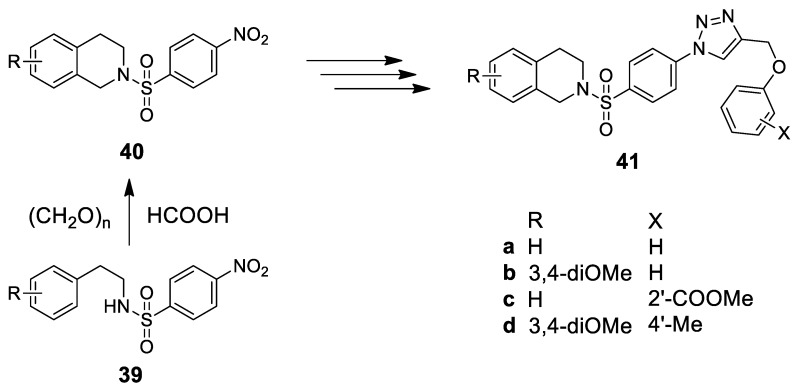
Synthesis of 1-substituted-*N*-benzene sulfonyl THIQ analogs for quantitative structure-activity relationship (QSAR) studies of toxicity.

Studies on the quantitative structure-activity relationship (QSAR) of *N*-benzenesulfonyl THIQ analogs **41** revealed the toxicity of several compounds against MOLT-3 cell lines (Scheme 9). The sulfonyl group was expected both to increase the electrophilicity of the iminium intermediate and to govern the bioactivity [35,36].

**Scheme 10:**
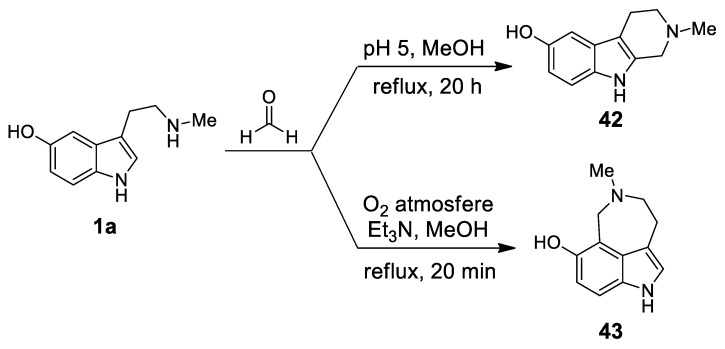
pH-Driven syntheses of P-S adducts constituent of black cohosh.

The structures of *N*-methyl-6-hydroxy-1,2,3,4-tetrahydro-β-carboline (**42**) and 3,4,5,6-tetrahydro-7-hydroxy-5-methyl-1*H*-azepino[5,4,3-*cd*]indole (cimitrypazepine, **43**), which were isolated from the root-extract of *Cimicifuga racemosa* (black cohosh), were confirmed by comparing the mass fragmentations with those of P-S adducts that were synthesized by the condensation of *N_ω_*-methyl serotonin and formaldehyde via a pH-guided nucleophilic attack to different sites (Scheme 10) [48].

**Scheme 11:**
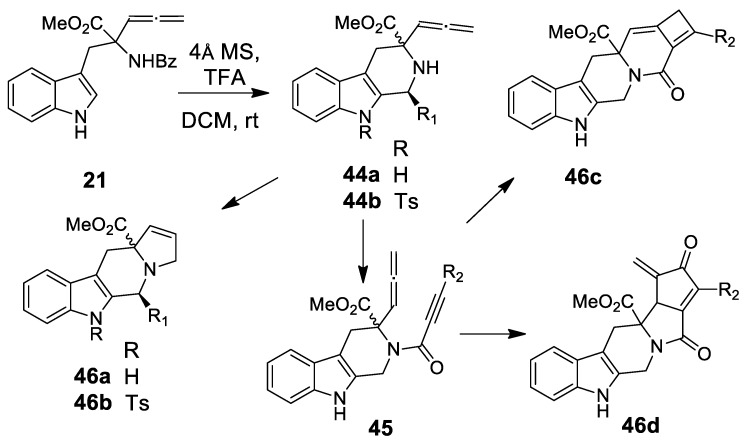
Synthesis of a library of THBC-containing compounds selected and evaluated *in silico*.

Conformationally locked allene-containing THBC (not shown), which was generated from the corresponding tryptophan derivative **21** via P-S cyclization, was subjected to cycloisomerization reaction to give tetrahydroindolizinoindole **44a**. The functionalization of **44a** with an alkyne and [2+2] cycloaddition of the allene-yne provided the tetrahydro-β-carbolinecyclobutanes **44b**, while cyclocarbonylation afforded α-methylenecyclopentenones **46d** (Scheme 11) [51].

A library of 34 THBC-containing compounds (**46**), including the *N*-tosyl derivatives **46b**, was synthesized utilizing a skeletal diversification strategy. *In silico* screening directed these compounds to the appropriate biological targets [51].

**Scheme 12:**
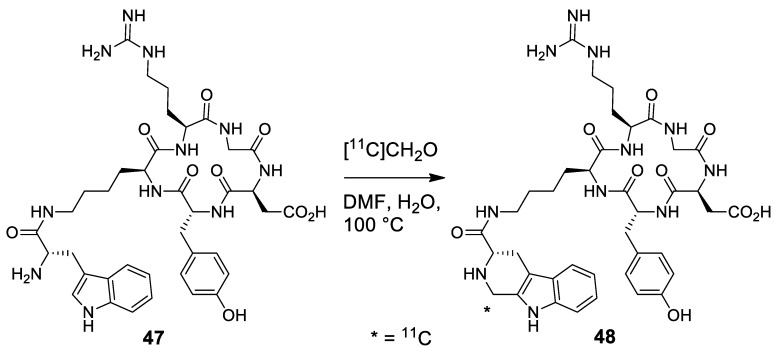
Synthesis of a radiolabeled RGD peptide containing [1-^11^C],2,3,4-tetrahydro-β-carboline-3-carboxilic acid, prepared trough a P-S reaction.

A model P-S condensation of tryptophan (Trp) and [^11^C]formaldehyde in neutral or acidic medium (TsOH or HCl) afforded the desired [1-^11^C],2,3,4-tetrahydro-β-carboline-3-carboxilic acid [1-^11^C]Tpi. Analogously, Trp·HCl-containing (RGD) peptide cyclo[Arg-Gly-Asp-D-Tyr-Lys] **47** successfully gave the labeled [1-^11^C]-containing RGD-peptide **48** (Scheme 12) [58].

Some references on P-S-driven synthesis of THIQ [38,40] and THBC [43,45,53,55] have been cited and/or discussed in other reviews [65,66].

### 3.2. Polyheterocycles

**Figure 2:**
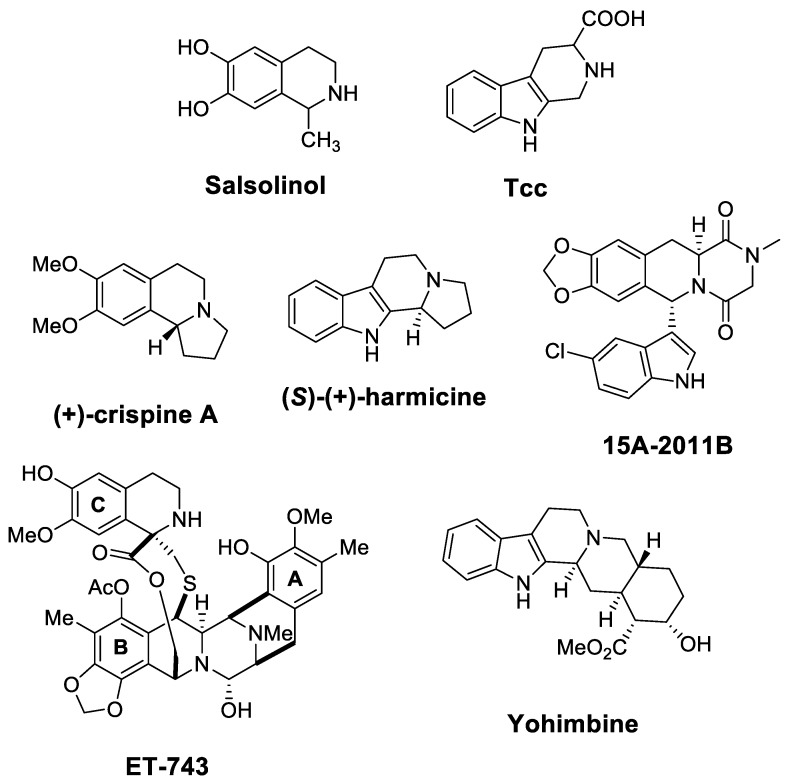
Different chemical structures containing THIQ/THBC motifs, with substituted or fused rings.

The THIQ/THBC motif does not only occur as a simple mono- or plurisubstituted ring system as in salsolinol (5,6-dihydroxytetrahydroisoquinoline) or Tcc (tetrahydro-β-carboline-3-carboxilic acid), but it can be fused with an additional five-membered (e.g., crispine A and/or harmicine) or 6-memberd ring (e.g., ISA-2011B, 1-indol-3-yl-6,7-methylenedioxy-1,2,3,4-THIQ diketopiperazine). The construction of fused rings on the THIQ or THBC skeleton is a key step in most of the total syntheses of natural products (isoquinoline and indole alkaloids), such as ecteinascidin 743 (ET-743) and yohimbine (Figure 2), which will be updated in the next section (*vide infra*).

The additional ring can be an aromatic ring as in protoberberines **53** [67,68], a pyrrole nucleus as in pyrroloisoquinolines (**55** [69,70]) and phenanthroindolizidines (**50** [71,72]), a piperazine or a diketopiperazine fused ring, as in phenathroquinolizidines **51** [72] and THIQ analogs **65** [73].

Phenanthroindolizidines (**50** [71,72]), phenanthroquinolizidines (**51** [72]), tetrahydroprotoberberines (**53** [67,68]), pyrroloisoquinolines (**55** [69,70]), and diketopiperazine-fused THIQs (**65** [73], *vide infra*) embody the tetrahydroisoquinoline skeleton, while indolizinoindoles (**57** [74,75]), THBC-imidazolinediones (**59** [76]), THBC-piperazinedione (**61** [77,78]), the tetracyclic indole alkaloids (*S*)-harmicine (pyrrole-fused THBC, [66,79]), and (*S*)-eleagnine (1-methyl THBC, [66,80] represent the THBC-containing polyheterocycles. The additional ring can be already present in the imine substrate before the P-S reaction (type A) or can be built on the THIQ/THBC skeleton exploiting the functionality of some substituents (type B). The concomitant ring-closures of the dihydropyridine and the additional ring are also feasible (type C).

**Scheme 13:**
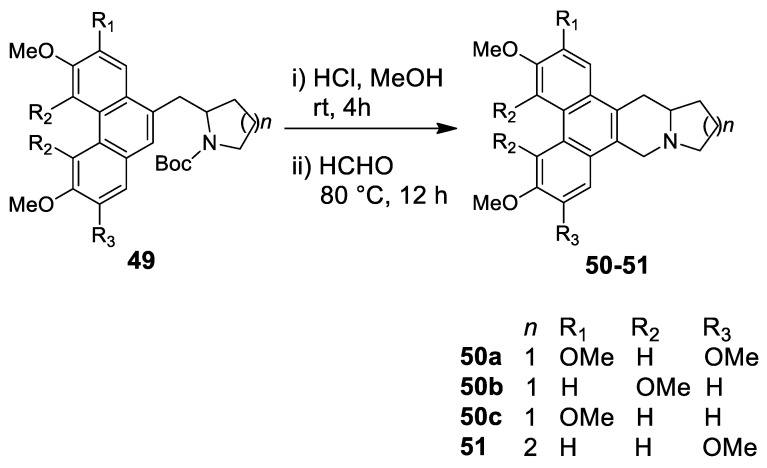
Phenanthroindolizidine (**50**) and phenanthroquinolizidine (**51**) alkaloids from phenanthrene derivatives via P-S cyclization.

Palladium-catalyzed annulation of highly methoxy-substituted 2,2′-diiodobiphenyls with alkynes provided phenanthrene derivatives **49a**, **49b**, and **49c**, which were transformed into phenanthroindolizidines (*n* = 1) **50a** (R_1_, R_3_ = OMe, R_2_ = H) and **50b** (R_1_, R_3_ = H, R_2_ = OMe), and phenanthroquinolizidine (*n* = 2) **51** (R_1_, R_2_ = H, R_3_ = OMe) by a P-S reaction (Scheme 13) [71]. Conversely, 13a-methylphenanthroindolizidine (*n*=1) **50c** (R_1_ = OMe, R_2_ = R_3_ = H; Scheme 13) was obtained by an enantioselective approach, including *inter alia,* an efficient stereoselective Seebach’s alkylation and P-S cyclization [72].

**Scheme 14:**
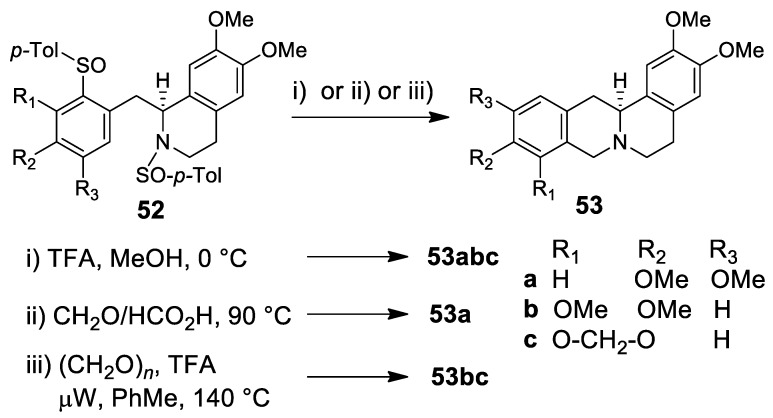
Protoberberine alkaloids via a sulfinyl directed P-S cyclization. * indicated the different conditions used for the reaction, reported in the bottom part of the scheme.

The participation of a sulfinyl group in an *ipso* electrophilic aromatic substitution reaction was the key step of the syntheses of (*S*)-(−)-xylopinine **53a** [67] and successively of (*S*)-(−)-tetrahydropalmatine **53b** and (*S*)-(−)-canadine **53c [68]**, natural products that belong to the tetrahydroprotoberberine alkaloids class (Scheme 14).

**Scheme 15:**
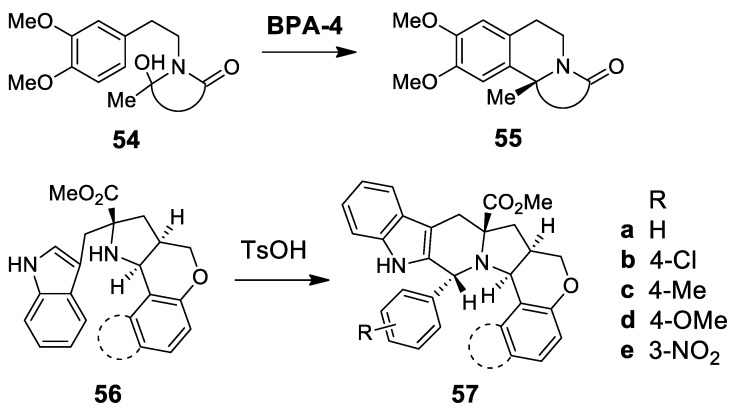
Pyrrolo[2,1-*a*]isoquinolines **55** and indolizinoindoles **57** as examples of previous extra ring closures.

BINOL-derived chiral Brønsted acids catalyze the intramolecular α-amido alkylation of a tertiary *N*-acyliminium ion containing a dimethoxylated phenyl ring as internal π nucleophile. The use of sterically congested **BPA-4** (see Scheme 33) was determinant in obtaining good level of enantioselectivity for the pyrrolo[2,1-*a*]isoquinoline **55** (Scheme 15, up) [69].

Novel chromeno[4,3-*b*]pyrroles **56** were synthesized by intramolecular 1,3-dipolar cycloaddition. A subsequent P-S cyclization in the presence of *p*-TsOH yielded indolizino [6,7] indoles **57** (Scheme 15, down). Chromenopyrroles and indolizinoindoles were both evaluated for their antimicrobial and antioxidant activities [75].

**Scheme 16:**
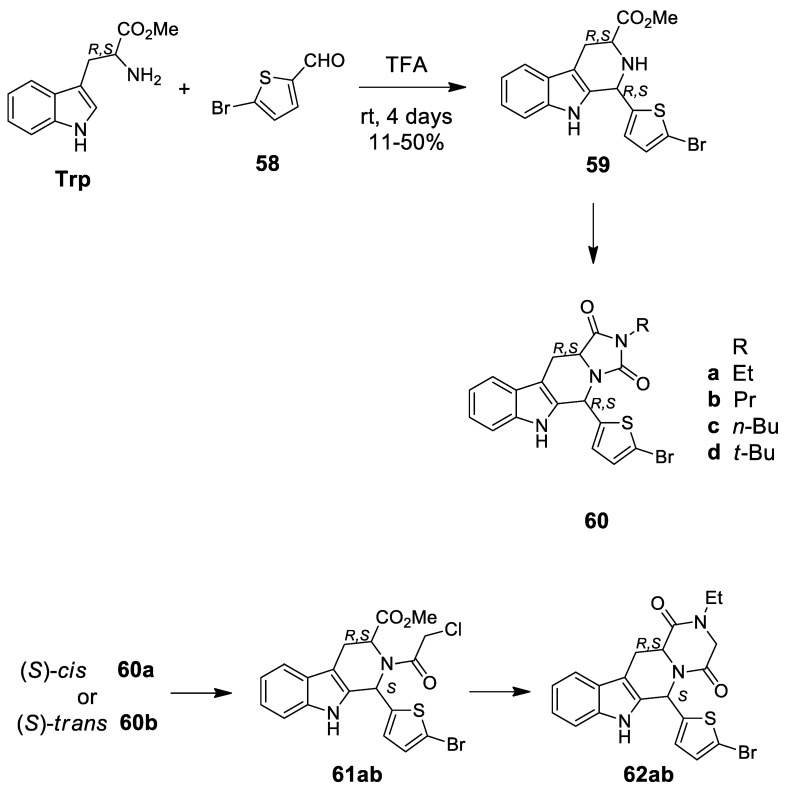
Synthesis of THBCs -with a pendant bromothienyl moiety- and their derivatives (imidazolidinone, piperazinedione, and chloroethanone) with PDE5 inhibitory activity.

In a type B building example, the four diastereomers (1*R*,3*R*; 1*S*,3*S*; 1*R*,3*S*; 1*S*,3*R*) of the 1,3-substituted THBC **59** were synthesized by a non-stereoselective P-S reaction to give the corresponding hydantoin derivatives **60a**–**d** (four diastereomers for each substituent R) by treatment with ethyl-, butyl-, *tert*-butyl-, and allyl-isocyanate. Conversely, the piperazinedione polycycles **62ab** (two diastereomers from (*S*)-*cis* and (*S*)-*trans* **60**) were prepared through the chloroethanone derivatives **61ab** (Scheme 16). The inhibitory activity on phosphodiesterase 5 (PDE5) of all the synthesized compounds was evaluated by structure-activity relationship (SAR) studies [76].

**Scheme 17:**
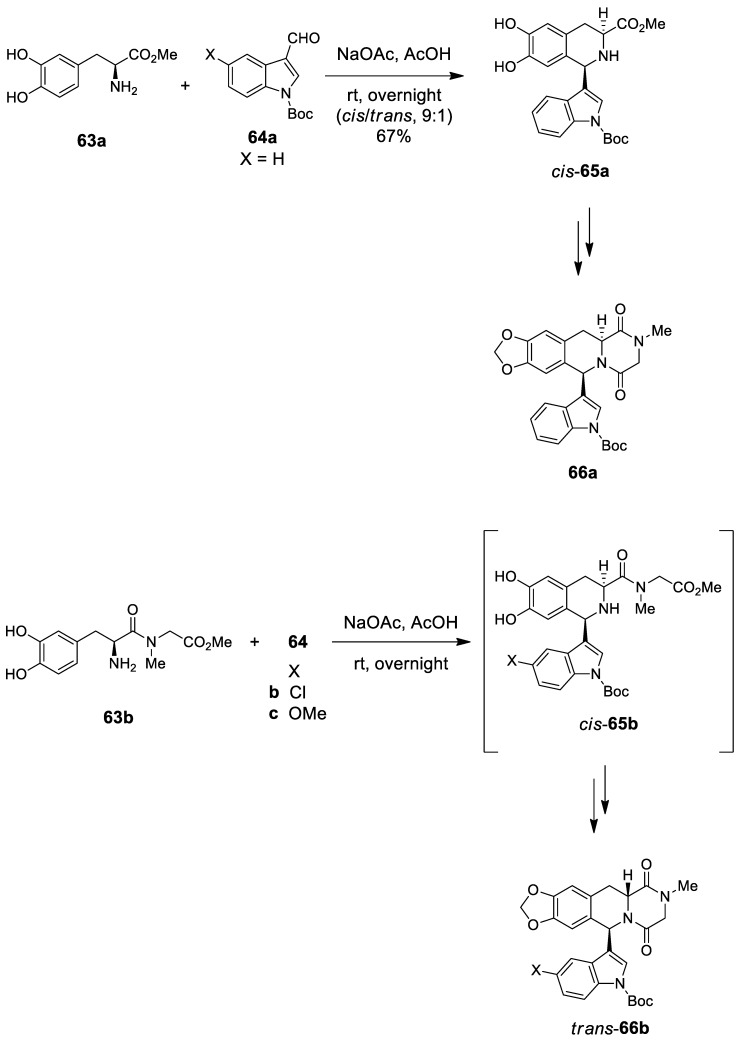
Synthesis of diketopiperazine fused THIQ derivatives through type A and C procedures.

A protocol for the P-S condensation between the methyl ester of L-DOPA **63a** and various *N*-Boc protected 1*H*-indole-3-carbaldehydes **64a** gave C-1 indol-3-yl substituted THIQs **66a** as a mixture of isolable *cis/trans* diastereomers in good yield. Following a Type-A procedure, the THIQ products **65a** can be in turn transformed into optically active diketopiperazine fused analogue **66a**. Alternatively, compounds **66b** were directly prepared drom L-DOPA derivative **63b** by condensation with *N*-Boc protected 1*H*-indole-3-carbaldehydes **64b** without isolation of the P-S precursors **65b** (Scheme 17) [73].

**Scheme 18:**
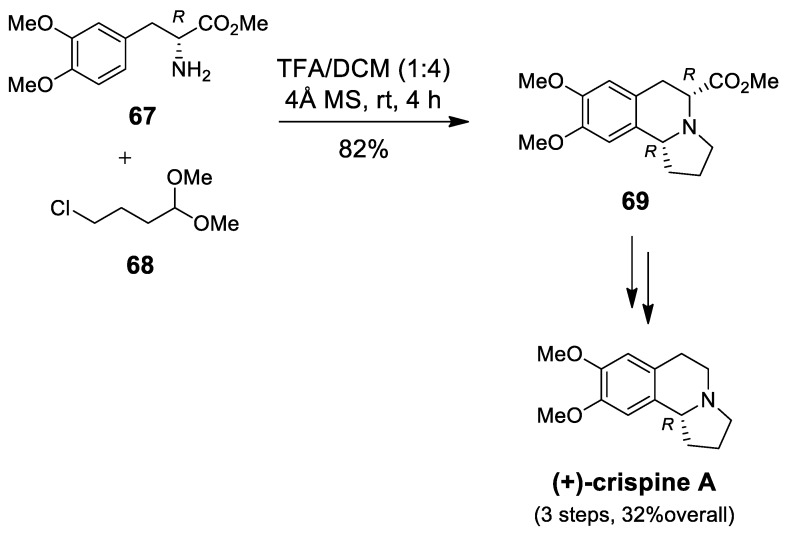
Concise enantiospecific stereoselective synthesis of (+)-crispine A.

The type C procedure foresees two simultaneous ring closures, as in the enantiospecific and stereo-selective P-S bis-cyclization between (*R*)-(−)-methyl-2-amino-3-(3,4-dimethoxyphenyl-propanoate **67** and 4-chloro-1,1-dimethoxybutane **68** preferentially, which provided the *cis*-tricyclic adduct **69** precursor of the natural product (+)-crispine A (Scheme 18). The unnatural antipode (−)-crispine A was similarly prepared from the commercially available (*S*)-(+)-amino acid ester [81].

**Scheme 19:**
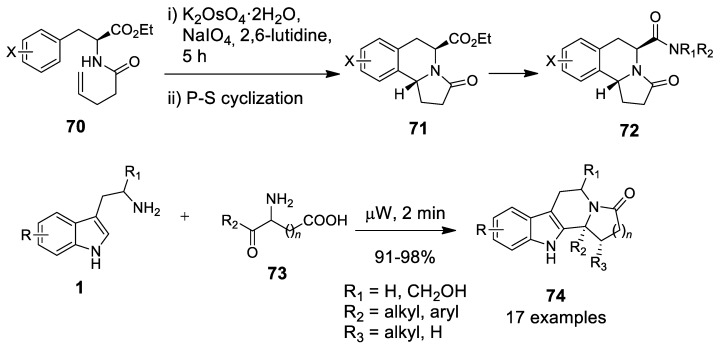
Double ring closure in THIQs and THBCs with an extra fused 2-pyrrolidone nucleus.

Two advanced hexahydropyrrolo[2,1-*a*]isoquinoline intermediates **71**, which incorporate two different halides for diversification, were synthesized through an oxidative cleavage/P-S cyclization sequence in high overall yields. The developed protocol was utilized to construct a 20-membered natural product-like molecular library (**72**, Scheme 19 up) [70].

An asymmetric or racemic diastereoselective reaction afforded indole alkaloids **74** via P-S reaction with short reaction times under solvent- and catalyst-free microwave irradiation (Scheme 19 down) [74].

### 3.3. Total Synthesis of Complex Alkaloid Natural Products

**Figure 3:**
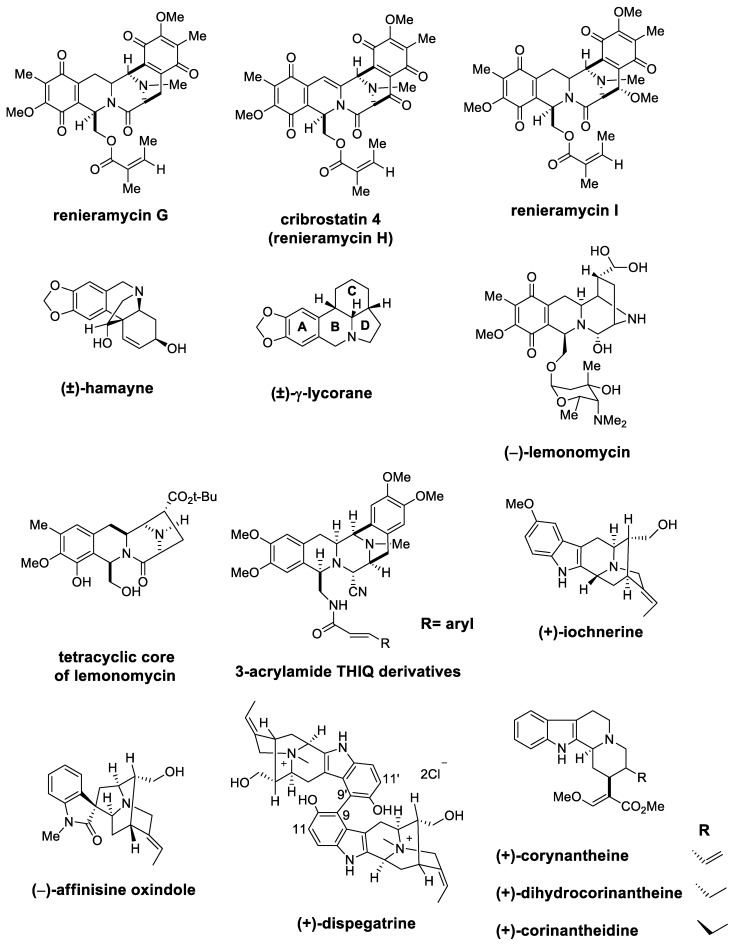
Targets of the total syntheses of THIQ/THBC-containing isoquinoline and indole alkaloids.

The two privileged nuclei, THIQ and THBC, can be embodied, even in the number of two or three units, into the framework of polycyclic complex structures as those reported in Figure 3. The total syntheses of structurally diverse compounds, all being endowed with a host of biological activities, include in most cases a step overcome by P-S methodology.

**Scheme 20:**
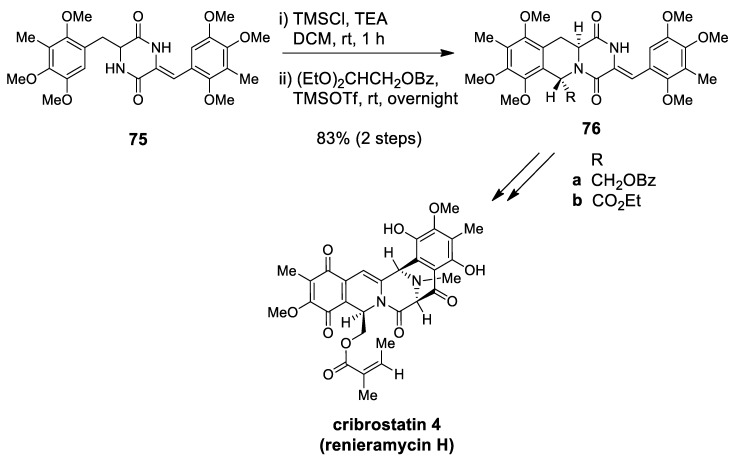
Construction of the core ring system of cribrostatin 4 via P-S cyclization.

In Table 1, the target products of multi-step syntheses are summarized together with some information on the P-S cyclization as well as on the type of related natural products.

(2011) Cyclization of 3-arylidene-6-methylpiperazinedione **75** with 2,2-diethoxyethyl benzoate afforded in two steps the P-S-adduct **76** as a single isomer (Scheme 20). The lactam **76** was used to construct the pentacyclic key intermediate framework (not shown) [82] and achieve the total synthesis of cribrostatin 4 [83] and renieramycin G (Figure 3) [84,85].

**Scheme 21:**
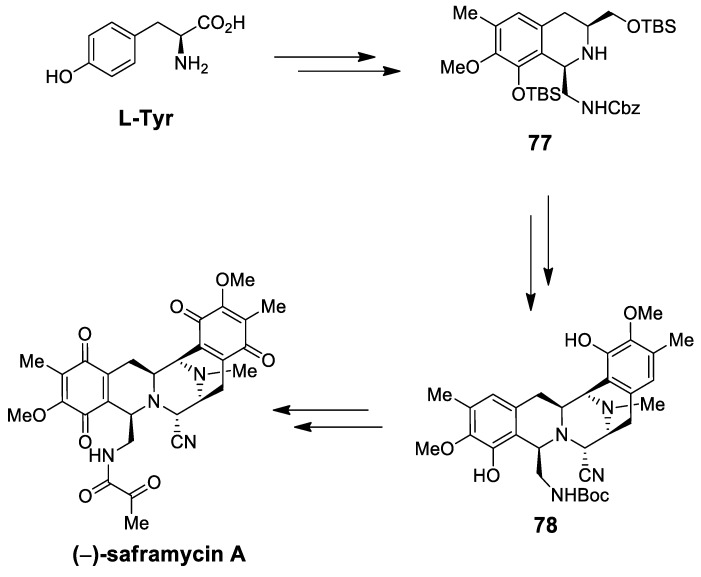
Asymmetric total synthesis of (−)-saframycin A by two P-S ring closures.

**Scheme 22:**
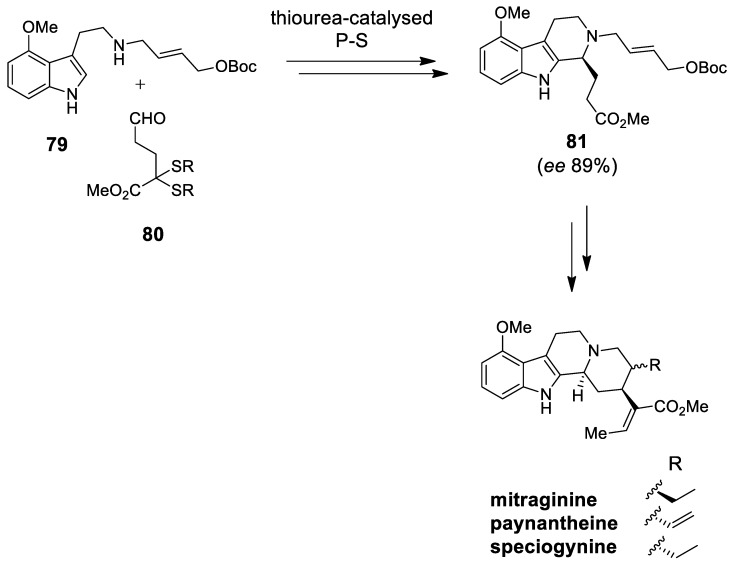
Enantioselective thiourea-catalyzed P-S reaction in the route to corynantheidine alkaloids.

- in the total synthesis of (±)-hamayne (Figure 3) the C-1 methylene was introduced via a P-S reaction at the end of 13 steps, just before deprotection of hydroxyl groups [86];
- the total synthesis of γ-licorane (Figure 3) was also completed by a P-S ring closure [87]; and,
- the asymmetric total synthesis of (−)-saframycin A (Scheme 21) from L-tyrosine involved stereoselective intramolecular and intermolecular P-S reactions to induce the correct stereochemistry at C-1 and C-11, respectively [88].

(2012) The key intermediate **81**, containing the THBC ring of mitragynine, paynantheine, and speciogynine (Scheme 22), was constructed via an enantioselective thiourea-catalyzed P-S cyclization involving the tryptamine derivative **79** and the aldehyde **80** [89].

**Scheme 23:**
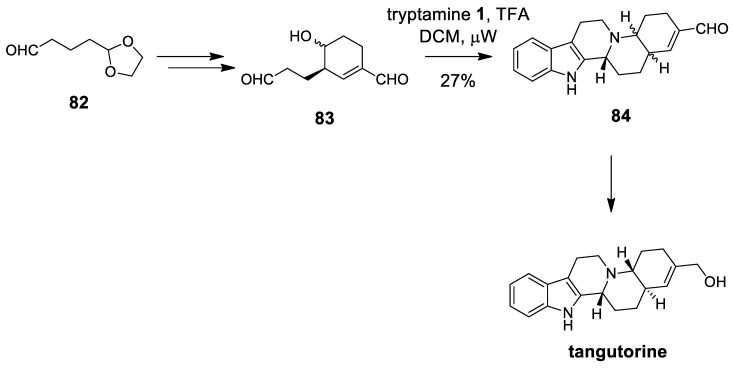
Total synthesis of tangutorine via P-S cyclization from monoprotected glutaraldehyde.

- Aldehyde **83**, as obtained from protected glutaraldehyde, is condensed with tryptamine (P-S reaction, TFA) to form the pentacyclic THBC **84**, a precursor of tangutorine (Scheme 23) [90].
- A thermodynamically controlled P-S reaction for the formation of the tetrahydroisoquinoline skeleton is among the steps that lead to the efficient and convergent total synthesis of (−)-lemonomycin (Figure 3) [91].

(2013) The tetracyclic core of lemonomycin (Figure 3) was synthesized from a known substituted tyrosinol through a 16-step sequence, which involved the P-S reaction *inter alia* [92].

**Scheme 24:**
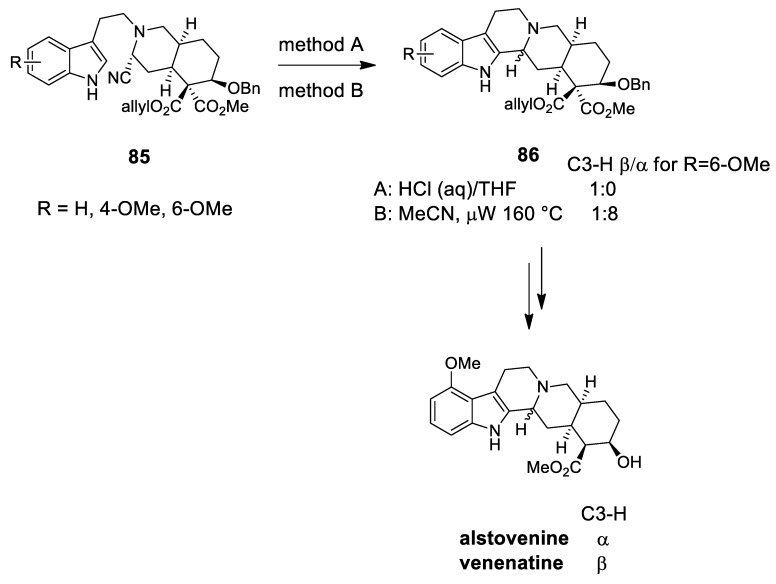
Divergent synthesis of alkaloids alstovenine and venenatine through P-S reaction.

In the first total syntheses of C-3 epimeric natural products venenatine and alstovenine (Scheme 24), the stereochemistry at C-3 of the yohimbinoid skeleton was effectively controlled in a P-S cyclization utilizing an aminonitrile intermediate [93].

**Scheme 25:**
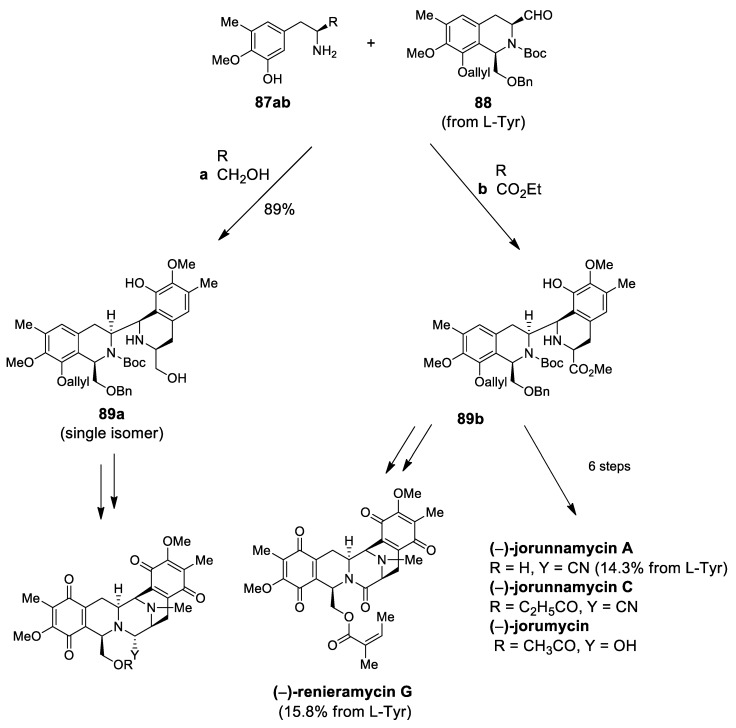
Synthesis of renieramycin-type alkaloids via stereoselective cyclization to P-S adducts.

- 24 compounds with diversified 3-aryl acrylic amide side chains of the simplified saframycin-ecteinascidin pentacyclic skeleton (Figure 3) were synthesized via a stereospecific route, starting from L-DOPA [94,95].
- In the framework of the synthesis of indole alkaloids such as the monomers (+)-locknerine, (+)-spegatrine, and the dimer P-(+)-dispegatrine (Figure 3), the mixture of *cis/trans* products from the P-S reaction was converted by treatment with TFA into the desired *trans* isomer [96].

(2013–2014) Three renieramycin type anticancer alkaloids, jorunnamycins A and C, and jorumycin, were synthesized by a new convergent approach, which couples for a highly regio- and stereo-selective P-S cyclization tryptamine **87a** and tetrahydroisoquinoline **88** to provide the intermediate **89a** as a single isomer (Scheme 25, up) [97].

**Scheme 26:**
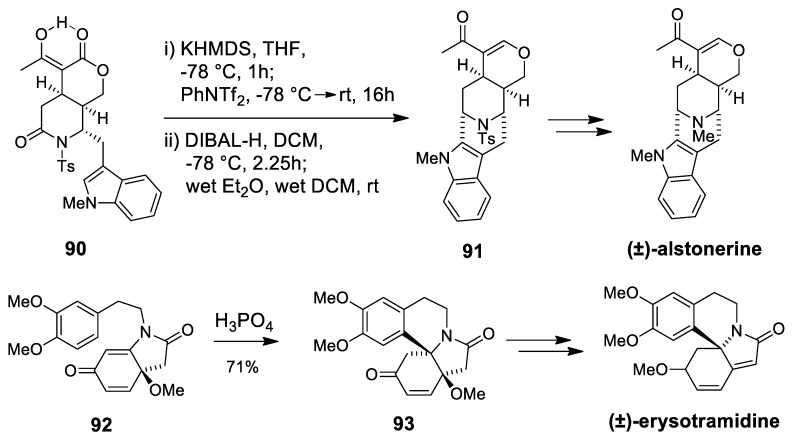
P-S cyclization as a key step in the synthesis of (±)-alstonerine (up) and erysotramidine (down).

**Scheme 27:**
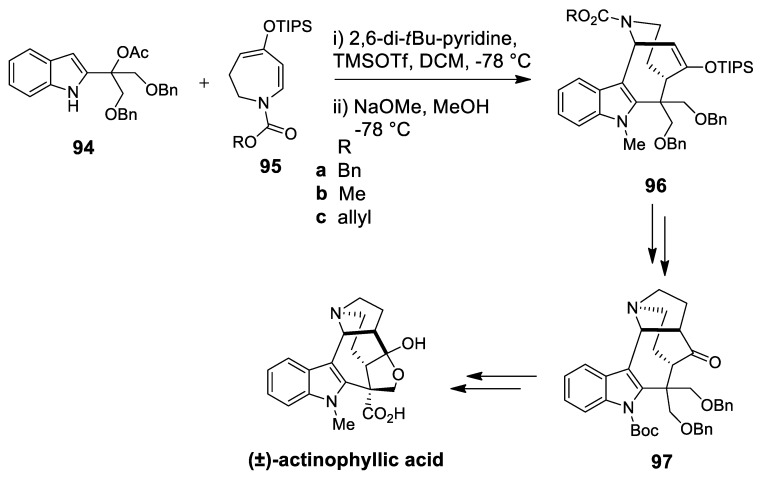
Lewis acid catalyzed cascade: key step in the total synthesis of (±)-actinophyllic acid.

**Scheme 28:**
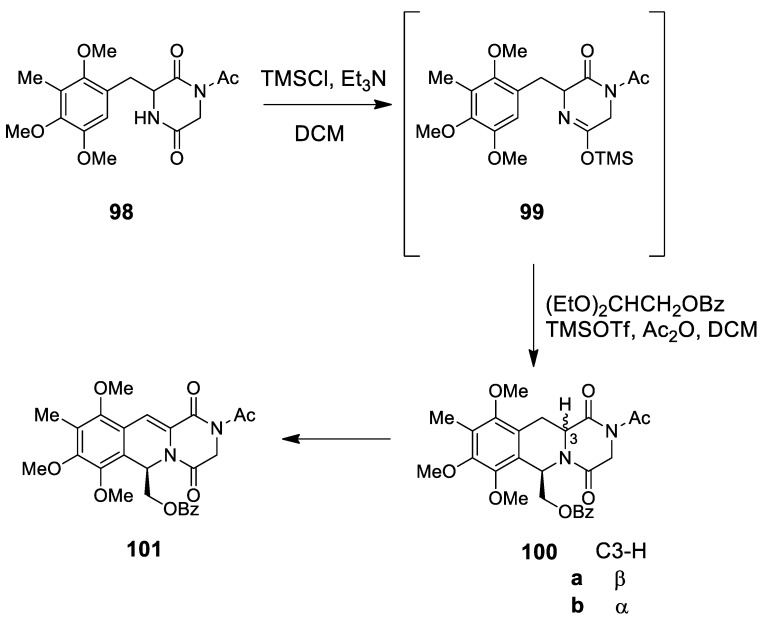
Construction of tricyclic unsaturated intermediate **102** in the synthesis of renieramycin I and cribrostatin 4.

Conversely, a temperature-dependent stereoselective P-S reaction of amino ester **87b** and aldehyde **88** afforded the cyclization product **89b**; the subsequent deprotection and the lactamization of this compound were the protagonists of a flexible protocol for the asymmetric synthesis of antitumor alkaloids (−)-jorunnamycin A and (−)-renieramycin G (Scheme 25, down) [95].

(2013) *O*-Triflation of indolyl derivative **90**, followed by the addition of DIBAL-H and wet Et_2_O-Rochelle salt work-up, gave the crude hemiaminal lactol, which, after quick treatment with wet DCM, underwent triflic acid elimination and P-S cyclization to afford the pentacyclic vinylogous ester **91**. The removal of the *N*-tosyl group and *N*-methylation of **91** ended a concise synthesis of the macroline-related alkaloid (±)-alstonerine (Scheme 26, up) [99].

(2014) The key steps of the synthesis of erysotramidine included two oxidative dearomatization processes that were mediated by a hypervalent iodine reagent, a novel tandem aza-Michael rearomatization, a P-S cyclization (**92**➔**93**) to produce the main tetracyclic system and the final stereoselective ketone reduction (Scheme 26, down) [100,101].

(2014) In a P-S type reaction, the electrophile **94** and the π-nucleophile **95** gave the tetracyclic intermediate **96**, which can be converted into the pentacyclic precursor **97** of the total synthesis of the indole alkaloid (±)-actinophyllic acid (Scheme 27) [102].

(2014–2015) Saito and coworkers presented an alternative large-scale approach for the total synthesis of cribrostatin four analogs, as well as C3-C4 unsaturated bis-*p*-quinone derivatives, such as renieramycin I. According to the results of previous studies [103], the treatment of the readily available compound **98** with trimethylsilyl chloride (TMSCl) in DCM in the presence of triethylamine afforded lactam intermediate **99**, which, in turn, treated with 2,2-diethoxyethyl benzoate in the presence of trimethylsilyl triflate (TMSOTf) and Ac_2_O gave the P-S product **100** as a diastereomeric mixture (**a/b**: 10/3) in 92% yield (Scheme 28) [104]. The conversion of the diketopiperazine THIQ **100** into unsaturated compound **101** was the first key step of the synthesis of renieramycin I and cribrostatin 4 (Figure 3) through the intermediate **76** (see Scheme 20).

(2011) The P-S reaction got a lead role in the total syntheses of (+)-yohimbine [105], (−)-corynantheidine [106,107], (+)-corynantheine, and (+)-dihydrocorynantheine [107] (active as antimicrobial against *Staphylococcus aureus*-induced infections [109]), which have been reviewed in Todd’s paper [65]. Analogously, the total synthesis of (−)-affinisine oxindole [108] was reported previously in the paper of Dalpozzo [66].

## 4. Dressing with Fashionable Clothes a Classic Reaction

*Not to be old fashioned*

*I’ll put something red*

(Emily Dickinson)

In the second century on stage, the P-S cyclization maintains its peculiar chameleonic quality, but to remain at the limelight needs to join the forces with in-fashion allies, such as the support of solid phases, the push of new catalysts, and mainly the impulse of new habits, such as the cascade sequences and the multicomponent reactions (MCRs).

### 4.1. A New Scenography: Updating the Solid Phase Strategy

Advances in support, protecting group strategies, and extensive optimization of chemical methodology have expanded the scope of solid phase chemistry from mere peptide preparation to the synthesis of pharmacologically relevant small molecules [110,111,112], as well as the total synthesis of natural products and their analogues [113,114]. Different versions of P-S reaction have found application in a procedure, where the solid-phase support can be chosen to modulate the reactivity of the three functional groups (aldehyde, amine, and aromatic nucleophile) [115].

In 2009, a paper from Nielsen et al. reviewed the methods used to generate *N*-acyliminium ion intermediates on solid support and gain access via the intra- or intermolecular P-S condensation products to diverse structures [116].

Meldal and coworkers focused on solid phase variants of the P-S reaction as rich sources for the construction of polyheterocyclic scaffolds. The authors synthesized pyrroloisoquinolines [117], bicyclic dipeptide mimetics [118], and polycyclic compounds containing THIQ, THBC, or analogous motifs [119]; they successfully applied the methodology to the formation of a range of (5,6,5)-, and (6-6-5)-fused heterotricyclic ring systems [120].

**Scheme 29:**
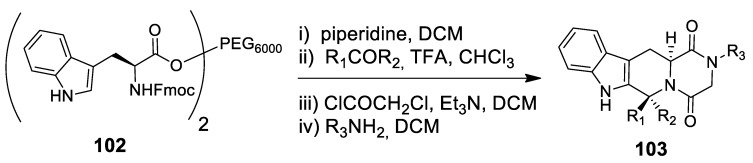
Synthesis of diketopiperazine-fused THBCs on soluble polymer support.

In the 2011–2015 year range, only two papers concerning an approach in solid phase of the intramolecular P-S reaction appeared [121,122]. In the first one by Chanda et al., polyethylene glycol-immobilized tryptophan ester **102** was combined with a variety of ketones by reflux in acidic chloroform, to give soluble polymer-supported THBCs in good yields [121]. Amination of the N_b_-chloroacetamide that was obtained by treatment with chloroacetyl chloride, followed by intramolecular cyclization and cleavage of the polymer, finally led to the costruction of a tetracyclic architecture. The reactions and the following synthesis of biologically promising diketopiperazine-fused THBC structures **103** are resumed in Scheme 29 [121].

**Scheme 30:**
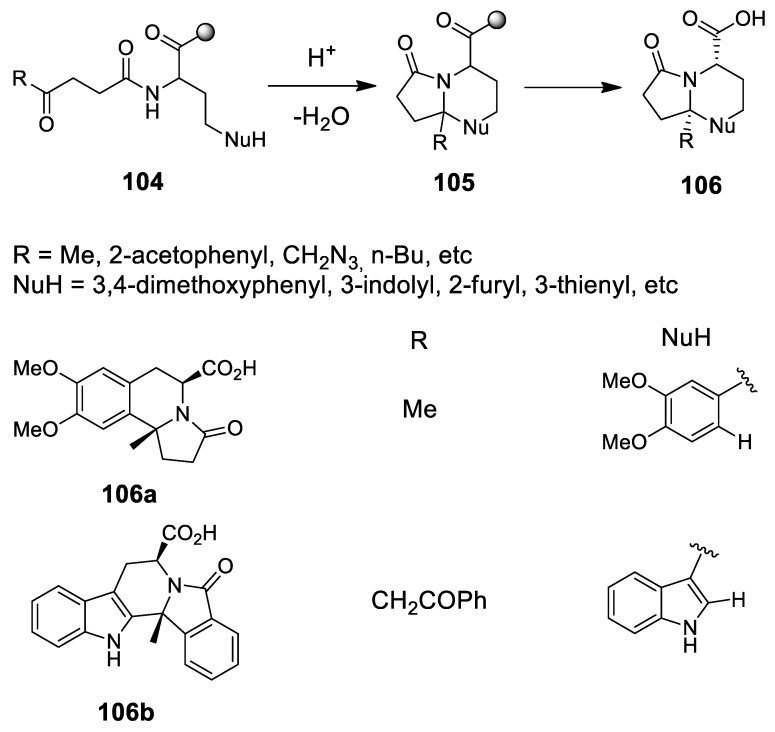
Intramolecular solid-supported amide ketone cyclization for the synthesis of heteropolycycles.

Another process (by Nielsen et al., 2012) relies on an efficient ketone-amide condensation, starting from γ-ketocarboxylic acids, immobilized on a solid support as **104**. Substrates, by treatment with a mild acid (HCOOH, the more efficient after a screening), generate the *N*-akyliminium ions, which undergo P-S type cyclization (Scheme 30) [122]. The cascade sequence afforded in a high stereoselective fashion pure products **106** after cleavage from the resin. The combination of various γ-ketoacid amides with a range of nucleophiles, including electron-rich aromatic (as 3,4-dimethoxy substitution for **106a**) and heteroaromatic rings (as indole nucleus for **106b**), led to a library of a range of pharmaceutically interesting heterocyclic scaffolds with exclusive diastereocontrol of the junction stereocenters [122].

### 4.2. Ruthenium-Catalyzed N-Acyliminium Cyclization

**Scheme 31:**
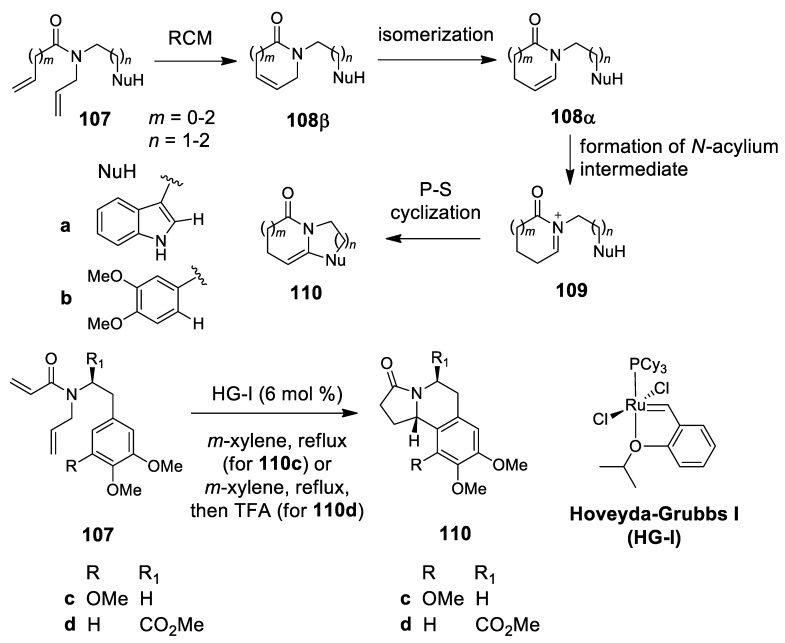
Ruthenium-catalyzed RCM/isomerization/*N*-acyliminium cyclization tandem sequence.

**Scheme 32:**
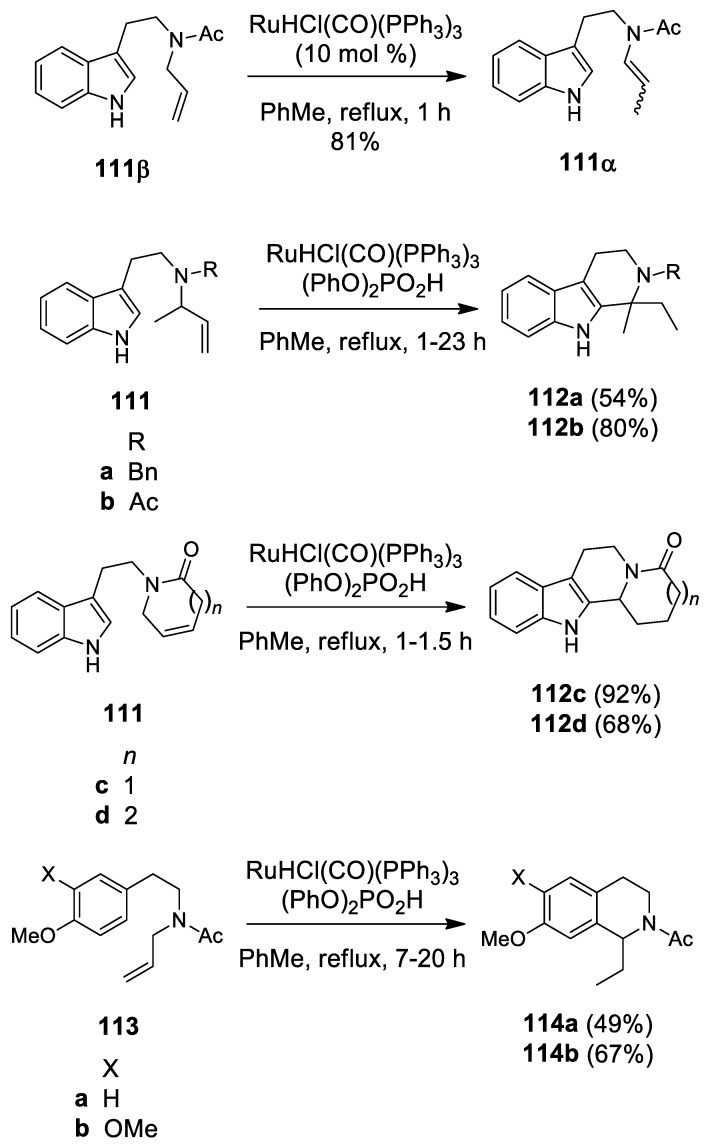
Ruthenium hydride/Brønsted acid-catalyzed synthesis of THBCs (up) and THIQs (down).

**Scheme 33:**
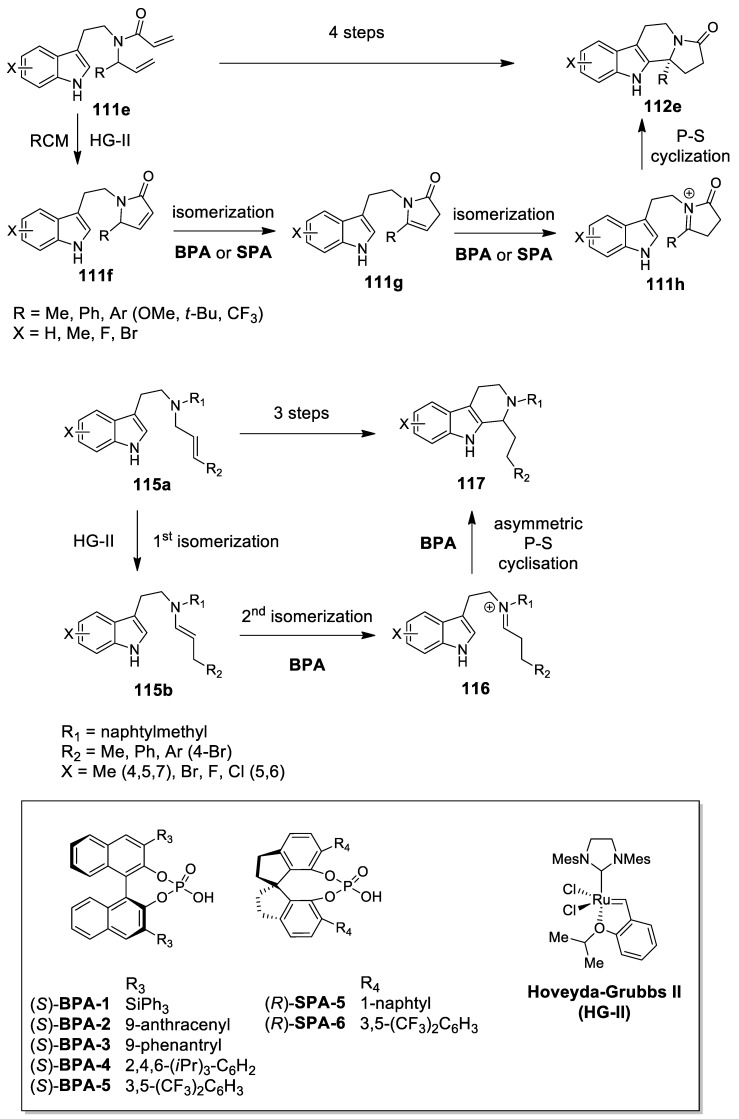
Synthesis of indolizinoindole derivatives via ruthenium/chiral phosphoric acid sequential catalysis.

(2011) The Nielsen group developed the synthesis of indolizinoindoles **110**, starting from the ruthenium alkylidene catalyzed tandem ring closing metathesis (RCM) of dienes **107** via *N*-acyliminium intermediates **109** (Scheme 31, up). In the case of indole substitution (**107a**) and five-membered ring (*n* = 0), the corresponding unsaturated lactams **108β** and **108α** are formed after a RCM and subsequent isomerization, respectively. The successive protonation or reaction of **108α** with Ru^+^ gave the reactive *N*-acyliminium species **109**, which were finally trapped by the tethered nucleophile to give the THBC tetracycle **110a** (*n* = 0). The homologous indole-based substrates **107a** (*n* = 1; *n* = 2) underwent RCM reactions, but not further conversions into THBCs, being required the conjugation of the double bond that formed in the RCM step with the lactam carbonyl. Hoveyda–Grubbs catalyst HG-I (at 5 mol%, in *m*-xylene at reflux) gave the cleanest and highest conversion **107a** (*n* = 1, 2)➔**110a** (95%; *n* = 1, 2). The trimethoxybenzene derivative **107c** (*n* = 1) also underwent the tandem reaction sequence to provide the tetrahydroisoquinoline derivative **110c** in good yield (64%, Scheme 31, down). By contrast, the conversion of substrate **107d** or other substrates bearing a heterocycle moiety (not shown) required the successive addition of TFA (1 eq.) and further 2 h heating to give the tricycle product **110d** (or the other corresponding cyclization products) in 98% yield and diastereomeric ratio > 20:1, or the other cyclization products [123].

(2012) As disclosed earlier, Nielsen and coworkers performed the metal-catalyzed isomerization of *N*-acyl-*N*-allyl tryptamines that were previously described (Scheme 32, down) [124].

(2013) In a third step, ruthenium hydride RuHCl-(CO)(PPh_3_) was found as an effective promoter of the isomerization **111β**➔**111α** since the Wilkinson’s catalyst [124] was no more efficient for the isomerization of the double bond of acylated allylamines **111**. The combination of the ruthenium catalyst (10% mol) together with the chiral phosphoric acid (PhO)_2_PO_2_H (30% mol) proved to be the most efficient for the transformation of the allylic amides **111ab** into THBCs **112ab**, but high temperature (toluene at reflux) was needed for the completion of the reaction. Finally, the cyclic allylic amides **111cd**, in optimized reaction conditions, gave the corresponding THBCs **112cd** in 92% and 68% yields, respectively (Scheme 32) [125,126]. The treatment of other electron-rich aromatics, such as the cyclic allylic amides **113ab** in the same conditions as before, afforded the corresponding tetrahydroisoquinoline derivatives **114ab**, which were isolated in moderate yields (49–67%, Scheme 32) [125,126]. Notably, N_b_-benzyl substituents in L-tryptophan derivatives proved to be more important than the N_b_-acetyl groups for the diastereoselectivity in a substrate-controlled version of the above tandem sequence.

(2012) A ruthenium alkylidene complex and chiral phosphoric acid catalyzed an enantioselective version of the RCM/isomerization/P-S cascade process (Scheme 33 up). You et al. showed that the steric bulk of the substituent adjacent to the nitrogen in the allylic system was crucial for the high enantioselectivity [125]. Subsequently (2013), the authors established the optimal conditions for the reaction of *N*-1-naphthylmethyl protected substrate **115b**: benzene as solvent, 0.5 mol% Hoveyda-Grubbs II (HG-II) and 5 mol % SPINOL-derived phosphoric acid (*R*)-**SPA-5** as binary catalyst, and 4Å molecular sieves as an additive. Consequently, the yield and the *ee* of THBCs **117** were considerably increased (Scheme 33) [126].

**Scheme 34:**
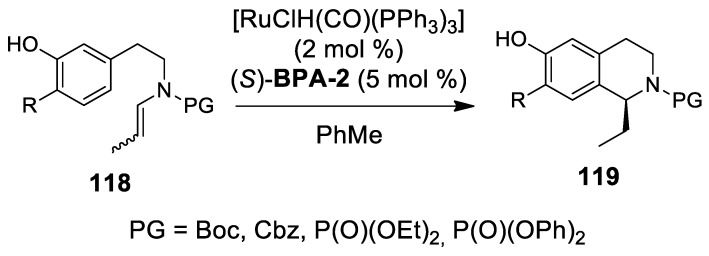
Relay catalysis of protected arylethylamines.

Toda and Terada described a similar enantioselective P-S type cyclization, catalyzed by ruthenium hydride complex and chiral phosphoric acid. Relay catalysis on protected arylethylamine **118** afforded the protected tetrahydroisoquinoline **119** (Scheme 34). The substitution pattern (*R*) of the aromatic ring, the bulky 9-anthranyl group of the chiral phosphoric acid catalyst, and the *N*-protecting group (PG) were surveyed and optimized [127].

### 4.3. All in One Pot

Bond formation via the intramolecular attack of *N*-acyliminium ion electrophiles by π-nucleophiles is a popular method for the construction of nitrogen-containing ring systems [128,129,130,131,132,133]. When *N*-acyliminium ion cyclization reactions are incorporated into cascade sequences, which are powerful strategies for the one-pot production of nitrogen-containing polycyclic ring systems emerge. At the beginning of the XXI century, the usual procedure for the synthesis of organic compounds, i.e., the stepwise transformation of the individual bonds in the target molecule, was being substituted by more efficient processes, where the readily available reactants are converted in a one-pot fashion into complex molecules. By a strict definition, a cascade reaction is a process in which multiple bonds are formed in sequence without changing conditions, adding reagents, or isolating intermediates. Most of the reported cascade sequences employ a single starting material, containing functional groups that are strategically positioned along a chain ending with an alkene moiety. The reactions enable two or more bond-forming and/or -cleaving events to occur in one vessel, where subsequent operations result as the consequence of the functionalities that formed in the previous step. The definition includes the prerequisite intramolecular to distinguish this reaction type from a multi-component reaction (*vide infra*). The cascade approach might assume different synonyms, such as tandem, domino, or one-pot and one-flask sequence, and it is also defined by the features of the key event, assuming the nuance of nucleophilic, electrophilic, cationic, anionic, pericyclic, radical, transition-metal catalyzed, enzymatic reaction, as well as being described as Heck reaction.

The main advantages of a cascade reaction in organic synthesis are given by reduction of time, labor, waste and resources, atom economy and the cleanliness of environmental tolerable procedures. Moreover, the process does not involve the workup and isolation of many intermediates and increases in efficiency bearing much complexity in effectively one step [128,129,130,131,132,133].

**Scheme 35:**
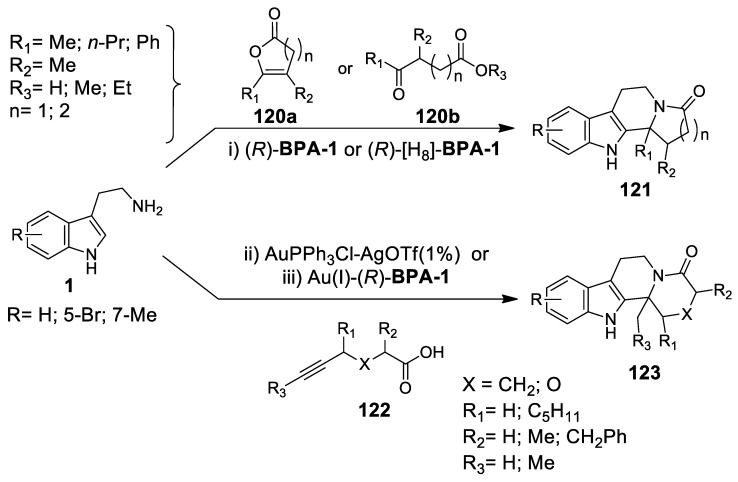
Gold (I)- and/or chiral Brønsted acid-catalyzed P-S cyclization cascade reactions.

Pioneering papers from Padwa and coworkers connected the one-pot strategy with P-S chemistry [134,135,136,137,138]. Dixon and coworkers introduced gold (I) catalyzed reaction sequences, where the metal ion activates alkyne, alkene, and allene functionalities under mild conditions and at low catalyst loading [139,140]. For instance, the group developed a direct enantio- and diastereoselective condensation of tryptamines **1** with five- or six-membered-ring enol lactones **120a** [140] or γ- and δ-keto esters **120b** [141], in the presence of chiral Brønsted acids, such as (*R*)-**BPA-1** (10 mol%) or *(R*)-[H_8_]-**BPA-1** (Scheme 33). The tetracyclic products **121** were obtained in good overall yields (53–99%) and moderate to high enantioselectivities (68–99% *ee*) (Scheme 35 up). The reactions were mostly run in toluene with the temperature ramp [140,141]. Notably, the enantioselective cascade reaction of the enol lactones was compatible with an in situ gold (I)-catalyzed synthesis from alkynoic acids **122** [140], which had been shown to undergo P-S condensation with amine tethered π-nucleophiles **1**, for the building of architecturally complex heterocycle structures **123** (Scheme 35 down).

#### 4.3.1. Au(I)-Catalyzed *N*-Acyliminium Cyclization Cascade

Historically, gold(I) complexes have emerged over the last decade as powerful tools for the synthesis of polyheterocyclic molecules. Au(I)-catalyzed reaction sequences have taken center stage due to the metal ion’s ability to activate various functionalities under mild conditions and at low catalyst loading. Moreover, the gold tolerance to oxygen, moisture, and many functional groups is very high, and this makes this metal an ideal candidate for the development of tandem catalytic strategies. The chemistry of gold-catalysis has been reviewed during the years from several papers [142,143,144,145,146].

**Scheme 36:**
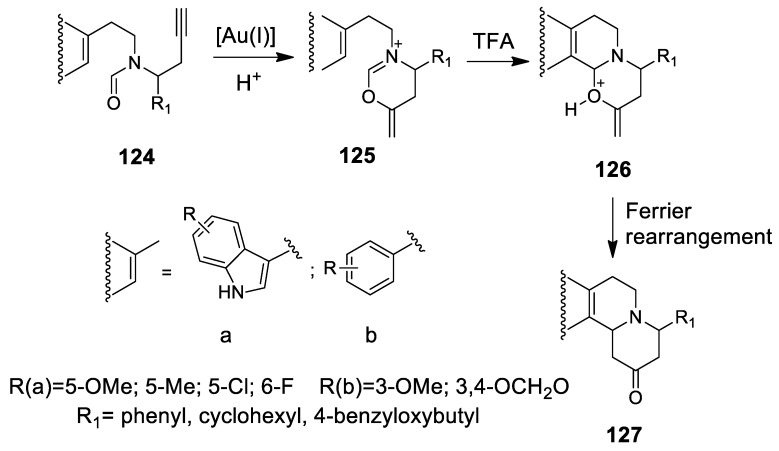
Gold catalysis-triggered cascade *en route* to bicyclic piperidine-4-ones.

**Scheme 37:**
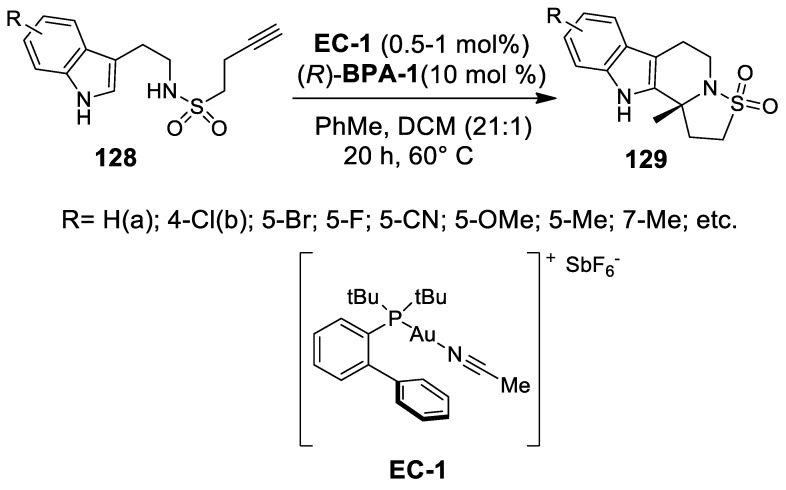
*N*-Sulfonyliminium cyclization cascade.

**Scheme 38:**
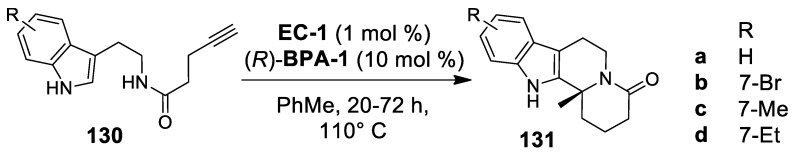
Synthesis of δ-lactams via 6-*exo*-cyclization cascade.

After the research of Dixon et al. [139,140,141], several papers [147,148,149,150,151,152,153,154,155,156,157,158,159,160] nourished the gold-fashion version of P-S cyclization in the years between 2011 and 2015.

(2012) Liu and Zhang reported a gold catalysis-triggered cascade reaction, where the formamide **124a** (indole, R = H) gave, in presence of *i*PrAuNTf_2_ or BrettPhosAuNTf_2_ (5 mol %, as catalyst) and TFA (as acid additive) in the optimized reaction conditions, the indole-fused hexahydroquinolizin-2-one **127a** (indole as aromatic moiety, R_1_ = H; 82% yield) after 24 h via the THBC intermediate **126a** (Scheme 36). Electron-rich aromatic rings, such as methoxy- and methylenedioxy-benzene yielded instead benzene-fused hexahydroquinolizin-2-ones **127b** (benzene as aromatic moiety, R = OMe, OCH_2_O) in synthetically serviceable yields. The new method was applied for the succinct and stereoselective synthesis of dihydrocorynantheol and a formal synthesis of yohimbine (Figure 3) and β-yohimbine (β-17-OH) [147].

(2013) The Dixon group developed a high enantioselective *N*-sulfonyliminium cyclization cascade, which provided complex and unusual sulfonamide scaffolds in excellent yield [148]. Treatment with Echavarren catalyst (**EC-1**, 10 mol% [149,150]) of a mixture of sulfonamide **128a** (R = H) and **BPA-1** (10 mol%) in toluene at 60 °C afforded the cascade product **129a** (R = H) in 84% yield and 88% *ee* (Scheme 37). The choice of sulfonamide over carboxylic acid amide was influenced by their abundance in medicinally relevant compounds and the lack of sulfonamide scaffolds via cyclization cascade. The versatility of the new methodology was verified on the chain extended amide analogue **130**, which was given by reaction cascades with **EC-1** (0.5–1 mol%) and (*R*)-**BPA-1** (10 mol%) in toluene at reflux the desired δ-lactams **131** in 86% yield and 66% *ee* (Scheme 38) [148].

**Scheme 39:**
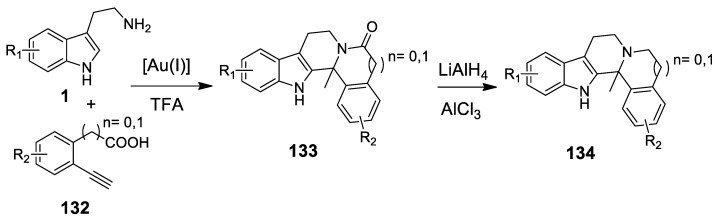
Gold(I)-catalyzed one-pot cascade for the synthesis of α_1A_ -AR antagonists.

The treatment of substituted tryptamines **1** and 2-ethynylbenzoic acids **132a** (*n* = 0) or 2-ethynylphenyl acetic acids **132b** (*n* = 1) by an efficient, facile gold(I)-catalyzed one-pot cascade protocol featured the formation of polycyclic privileged structures **133** with high yield and broad substrate tolerance (Scheme 39). Selected target molecules **134**, which were obtained after reduction, were validated as α_1_-adrenergic receptor antagonists [151].

**Scheme 40:**
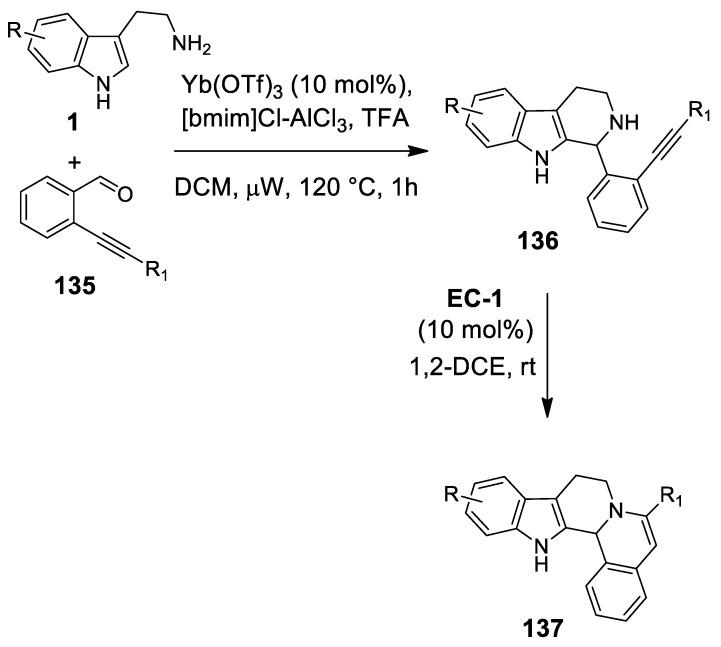
Two-step sequence for the synthesis of indoloquinolizines.

**Scheme 41:**
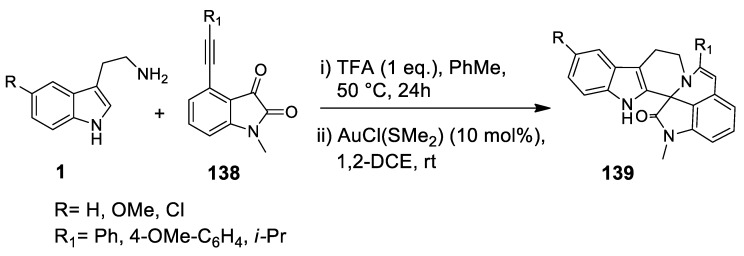
Two-step protocol for the synthesis of hexacyclic indoloquinolizines containing spirooxindole ring-system.

A P-S-type reaction was invoked for the final aromatic 1,7-cyclization step in the proposed mechanisms of gold(I)-catalyzed [5 + 2] cycloaddition of propargyl esters or acetals with imines (not shown), leading to benzo-fused azepine derivatives [152].

(2015) Waldmann and coworkers proposed a reaction sequence, where, in a first step acetylenic aldehydes **135** and tryptamine, yielded in a P-S reaction THBCs endowed with an alkyne substituent, which could be given via a hydroamination reaction the indoloquinolizine (IQZ) scaffold. Actually, microwave heating (120 °C) of a mixture of tryptamine (**1a**, R = H) and *o*-2-phenylethynyl benzaldehyde (**135a**, R_1_ = Ph) in DCM with 10 mol% of Yb(OTf)_3_ after the addition of the ionic liquid [bmim]Cl-AlCl_3_ provided the THBC **136** (R = H, R_1_ = Ph; 74% yield) within one hour (Scheme 40) [153]. However, the second step, i.e., the hydroamination reaction of the P-S adduct, did not occur in the presence of ytterbium complexes [154,155]. By contrast, after a survey of selected gold complexes, the catalyst **EC-1** (10 mol%) (Scheme 37) gave, at room temperature, the desired IQZ **137a** (R = H, R_1_ = Ph; 62%). The authors explored the utility of the two-step protocol for the synthesis of hexacyclic indoloquinolizines, where a spirooxindole ring system is fused to a THBC moiety. Tryptamines **1** and isatins **138** afforded the hexacyclic heterocycle **139** (Scheme 41) [153].

**Scheme 42:**
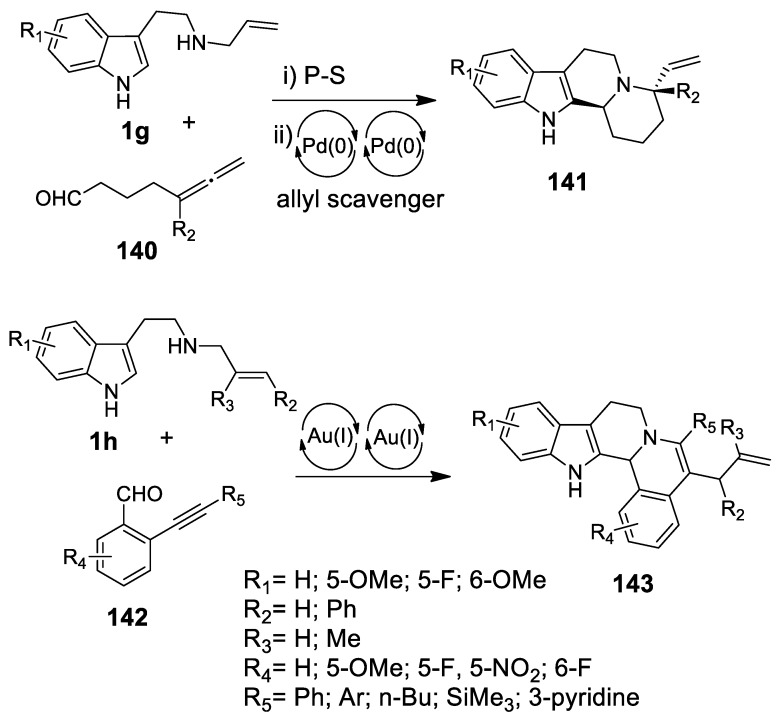
Synthesis of polycyclic indole derivatives by relay catalysis with Pd(0) or Au(I).

Guinchard and coworkers used allenals **140** as bifunctional key building blocks for the synthesis of polycyclic chiral architectures **141** (Scheme 42) in a reaction that combines an asymmetric phosphoric acid catalyzed P-S reaction and a self-relay palladium catalyzed cyclization [56,57].

Inspired by Waldmann and Kumar [153], ending in pentacyclic derivatives **143** (Scheme 42) [158], the authors proposed a gold-catalyzed cascade, where the P-S reaction between *N*-allyl tryptamines **1h** and *O*-alkynyl arylaldehydes **142** is followed by cyclization with a concomitant allyl transfer to give compounds **143** in good yield (Scheme 42). **EC-1** (Scheme 37) was the sole catalyst for both of the reactions. Further optimization came from the use of the stable cationic catalyst [(Ph_3_P)Au(NTf_2_)] (**EC-2**), as well as molecular sieves (4Å) [158].

#### 4.3.2. Michael Addition/Pictet-Spengler Reaction Sequences: Update 2011–2015

**Scheme 43:**
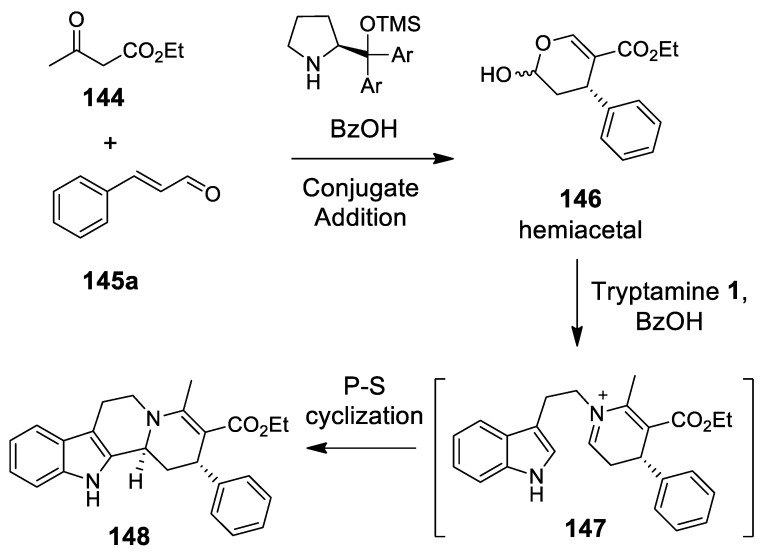
Michael addition/*N*-acyl iminium P-S cyclization sequence for one-pot complex heterocycles synthesis.

**Scheme 44:**
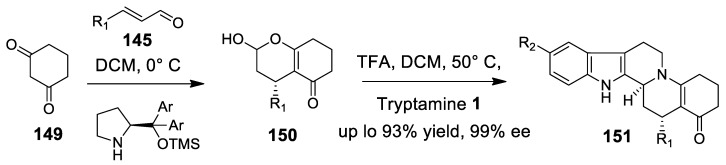
Diastereoselective cascade synthesis of indoloquinolizines.

The combination of a base-catalyzed intermolecular Michael addition reaction—featured by an α,β-unsaturated carbonyl compound and a suitable amide pronucleophile—with an acid-catalyzed intra-molecular *N*-acyliminium ion P-S cyclization of the resulting adduct, was a quite popular cascade sequence for building complex multiring heterocyclic molecules in one-pot and under mild conditions. The β-ketoester **144**, cinnamic aldehyde **145a**, the Michael adduct **146**, and the final indoloquinolizidine product **148** feature the sequence (Scheme 43) [159].

(2011) The Zhao group, after β-ketoesters [159] and dialkyl malonates [160], investigated alkyl propiolates [161] and β-keto amides [162] as carbonyl partners of α,β-unsaturated aldehydes for the asymmetric organocatalyzed synthesis of indoloquinolizidines derivatives. By a similar pathway, the group developed a cascade sequence between cyclic hemiacetals **150** and tryptamine **1** to provide efficient access to highly substituted diazaindeno[2,1*-a*]-phenanthrenes **151**. Aromatic and aliphatic hemiacetals **150** were both prepared by the asymmetric organocatalyzed conjugate addition of cyclic 1,3-diketones to α,β-unsaturated aldehydes **145** (Scheme 44) [163].

Contemporaneously Rueping et al., developed a similar methodology for the efficient synthesis of functionalized indolo [2,3-*a*]quinolizidine skeletons in a one-pot operation [164]. The same group successfully applied the cascade reaction to other nucleophiles [66,165].

**Scheme 45:**
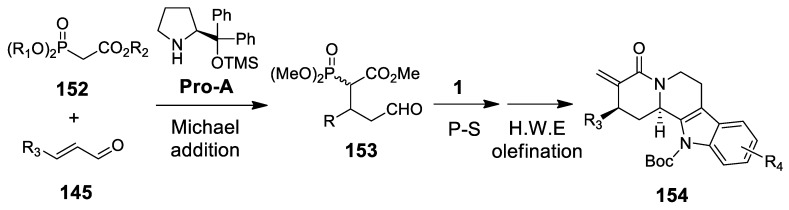
Michael adducts provide via P-S cyclization and H.W.E. olefination indolo[2,3-*a*]quinolizine derivatives.

Michael addition of trimethyl phosphonoacetate **152** to α-β-unsaturated aldehydes **145**, catalyzed by (*S*)-(−)-α,α-diphenyl-2-pyrrolidinemethanol trimethylsilyl ether (**Pro-A**), afforded enantiomerically enriched adducts **153**, which were employed in three different protocols leading to biologically important α-methylene-δ-lactones and δ-lactams. In one pathway, the indolo[2,3-*a*]quinolizine scaffold **154** was accessed by the P-S reaction with tryptamine **1**, followed by Horner-Wadsworth-Emmons olefination (Scheme 45) [166].

The assembly by Zhu et al. of medicinally important butyrolactam-fused indoloquinolizidines in a highly stereo-controlled organocatalytic one-pot Michael/P-S sequence has been previously described [66,167].

**Scheme 46:**
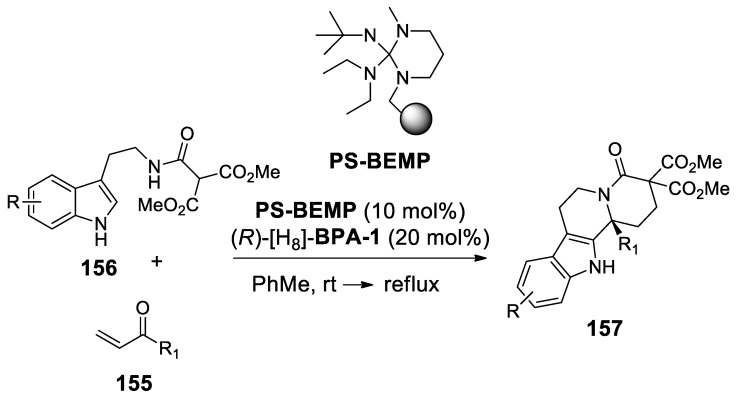
Novel size exclusion phenomenon for enantioselective acid/base cascade catalysis.

Although many examples of cascade sequences that were catalyzed by a single chemical entity have been reported [134,136], which involve more than one mutually compatible catalyst are much less common [168,169]. With the aim of overcoming the problem of annihilation (catalyst quenching), due to the simultaneous use of both strongly basic and strongly acidic reagents, the Dixon group employed site isolated base and acid (SIBA) catalysis [170,171]. The group developed cascade reactions that incolve the polymer supported 2-*tert*-butylimino-2-diethyl-amino-1,3-dimethyl-perhydro-1,3,2-diaza-phosphorine (PS-BEMP) and (*R*)-**BPA** or bulky derivatives, such as (*R*)-**BPA-1** and (*R*)-[H_8_]-**BPA-1** [172]. The authors hypothesized that the system would provide the necessary site isolation of mutually destructive acidic and basic functional groups and a size exclusion (molecular sieving) phenomenon would operate between **PS-BEMP** and (*R*)-**BPA-1**, but not between the first one and diphenyl phosphate (DPP). The model reaction of pro-nucleophile malonamate (**156a**, R = H) with methyl vinyl ketone (MVK, **155a**, R_1_ = Me) required the following optimal conditions: **PS-BEMP** (10 mol%), (*R*)-**BPA-1** (10 mol%), the addition of MVK (3 eq) at room temperature for 24 h, *N*-acyliminium cyclization at reflux in toluene for 24 h. The product **157** (R_1_ = H) was obtained in 81% yield and 57% *ee* (Scheme 46). The teatment of a set of malonamate nucleophiles **156** with methyl vinyl ketone (MVK, **155a**, R_1_ = Me) and ethyl vinyl ketone (EVK, **155b,** R_1_ = Et) in the presence of **PS-BEMP** and (*R*)-[H_8_]-**BPA-1** investigated the scope of the reaction. In general, yields that range from 73 to 80% and *ee* from 56 to 76% were achieved [172].

**Scheme 47:**
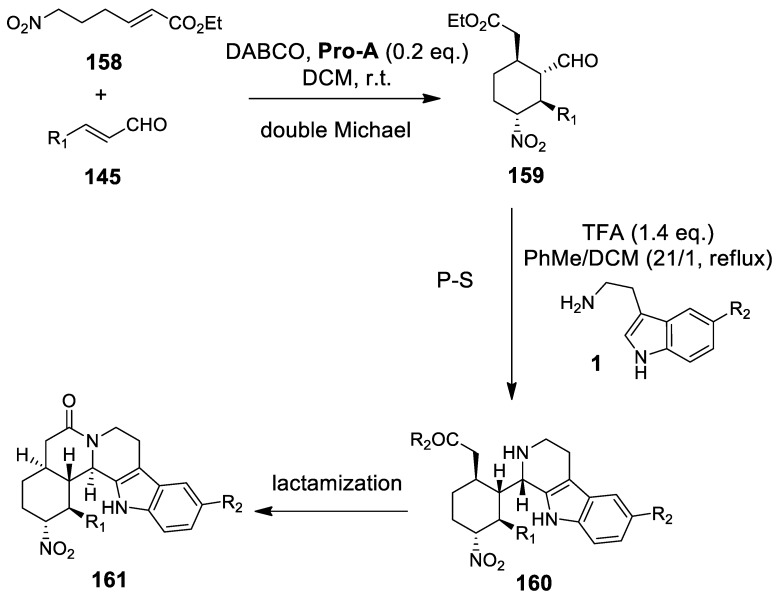
Synthesis of dodecahydro[a]indolo[3,2-*h*]quinolizine via a domino double Michael/P-S/lactamization reactions sequence.

Liao and coworkers reported an organocatalytic domino double-Michael/P-S/lactamization reaction sequence leading to dodecahydrobenz[*a*]indolo[3,2-*h*]quinolizines in good yields and excellent diastereoselectivities and enantioselectivities (up to >99% *ee*). The optimization of the double Michael reaction of nitroalkenoate **158** and cinnamaldehyde **145a** (R_1_ = Ph) required **Pro-A** as catalyst, DABCO as additive, and DCM as solvent for 44 h reaction to give **159a** *(*R_1_ = Ph) in 89% yield with 99% *ee*. The one-pot reaction strategy was achieved via the addition of toluene to the reaction mixture, followed by treatment with tryptamine **1** and TFA, to provide cyclized product **160a** (R_1_ = Ph) in 77% yield with >99% *ee*. Finally, the target products **161** were obtained after lactamization (Scheme 47). The one-pot domino reaction protocol was repeated with various α,β-unsaturated aldehydes (R_2_ = Ar, 2-furyl), obtaining high yields and stereoselectivities. The highly enantioselective transformation and the highly functionalized pentacyclic benzoindoloquinoline products render this reaction sequence a potential protocol for future synthetic applications of “inside α-yohimbane” intermediates [173].

**Scheme 48:**
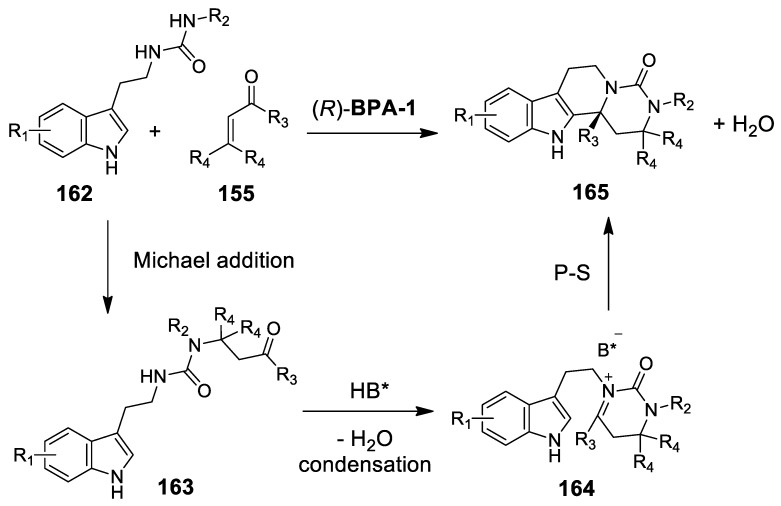
Enantioselective Michael addition/iminium ion cyclization of tryptamine-derived ureas.

Tryptamine-derived urea **162a** (R_1_ = R_2_ = H) and methyl vinyl ketone **155a** (MVK, R_3_ = Me, R_4_ = H) produced the best results in toluene with BINOL phosphoric acid when the heating was increased to 110 °C. Catalyst screening revealed that optimal enantiocontrol was associated with (*R*)-**BPA-1** and/or (*R*)-[H_8_]-**BPA-1** (both at 10 mol%) and optimal concentrations of substrates **162** were 5 mM (**162**) in the presence of 5 eq of MVK **155a**. Under these conditions, compound **165a** (R = R_1_ = R_2_ = H, R_3_ = Me) was obtained in 76% yield and 73% *ee*, through the intermediates **163** (Michael addition to the distal nitrogen) and **164** (condensation of the ketone with tryptamine N_b_) (Scheme 48). The scope of the reaction was surveyed while using an array of substituted ureas **162** and enones **155** [174].

**Scheme 49:**
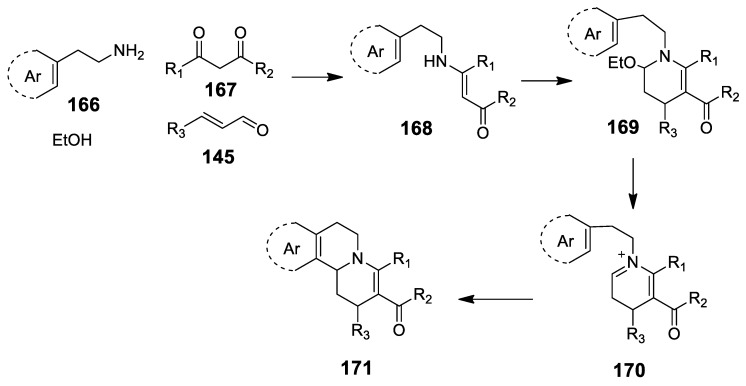
Sketched synthesis of areno[2,3-*a*]quinolizines by the use of cerium (IV) ammonium nitrate (CAN).

**Scheme 50:**
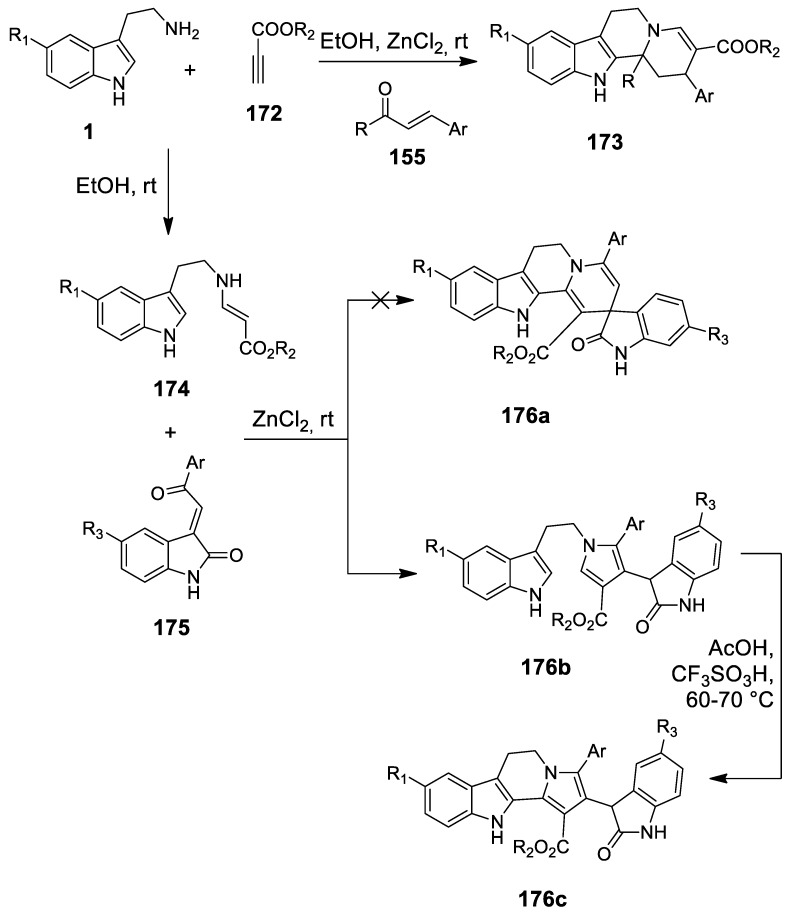
Construction of hexahydroindolo[2,3-*a*]quinolizines and dihydro-5H-indolizino[7,8-*b*]indoles.

**Scheme 51:**
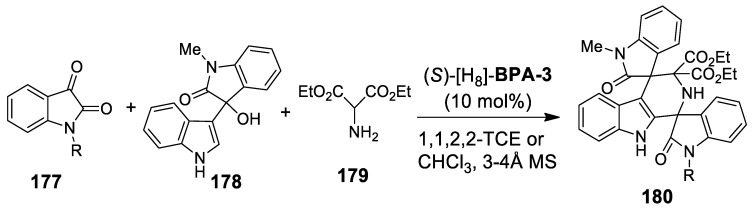
Design and practice in the construction of THBC-fused bispirooxindoles.

Menéndez and coworkers highlighted cerium (IV) ammonium nitrate (CAN) as an excellent catalyst for the fast synthesis of β-enaminones **168 [175]** and performed the one-pot, efficient preparation of 1-alkyl-6-ethoxy-1,4,5,6-tetrahydropyridines **169** from acyclic precursors, i.e., primary amines **166**, β-dicarbonyl compounds **167**, α,β-unsaturated aldehydes **145**, and alcohols (as sketched in Scheme 49). The combination of this preparation with a P-S cyclization makes up a domino process that involves the generation of an iminium cation **170** from the tetrahydropyridines **169** and culminates in the direct one-pot preparation of a variety of benzo[*a*]- or indolo[2,3-*a*]quinolizidines **171** [176].

(2014) Yan et al. performed a facile synthetic procedure that involves the sequential Michael addition and P-S reactions of β-enamino ester generated in situ. The one-pot three component reaction of tryptamines **1**, alkyl propiolates **172**, and α,β-unsaturated aldehydes **155a** (R = H)**,** as well as arylidene-acetones **155b**, (R = Me) afforded the functionalized 1,2,6,7,12,12*b*-hexahydro-indolo[2,3-*a*]quinolizines **173** in moderate to high yields and with high diastereoselectivity (Scheme 50 up) [177]. Hunting for a new efficient domino reaction, the group envisioned that spiro-indolo[2,3-*a*]quinolizine-oxindoles **176** could be synthesized from a similar reaction of in situ generated β-enaminoesters **174** with oxindoles **175**. However, the domino reaction of tryptamine **1a**, alkyl propiolates **172**, and 3-phenacylidene-oxindoles **175** in the presence of anhydrous ZnCl_2_ provided, instead of the expected compound **176a,** the functionalized 2-pyrrolo-3′-yloxindoles **176b**, which, in turn, can be converted into the corresponding 6,11-dihydro-5*H*-indolizino[8,7-*b*]indoles **176c** via a CF_3_SO_3_H catalyzed P-S cyclization process (Scheme 50, down). By contrast, when arylamines replaced tryptamines **1**, the one-pot domino reaction only afforded the corresponding 2-pyrrolo-3′-yloxindoles (not shown) [178].

(2014) The first catalytic asymmetric construction of a new class of bispirooxindole scaffold that incorporates a THBC moiety was established via a BPA-catalyzed three-component Michael/P-S cascade sequence, which afforded the structurally complex and diverse target compounds in excellent stereoselectivities (*dr* > 95:5; *e.r.* up to 98:2) [179]. *N*-benzylisatin **177**, *N*-methylisatin-derived 3-indolylmethanol **178**, and diethyl-2-aminomalonate **179** in CHCl_3_ at 45 °C in the presence of (*S*)-[H_8_]-**BPA-3** (with bulky 9-phenanthrenyl groups at 3,3′-positions; Scheme 33) afforded the desired bispirooxindoles **180** with good yield (72%) and stereoselectivity (*ee* 82%) (Scheme 51). 1,1,2,2-tetrachloroethane (TCE) and CHCl_3_ were alternated as needed, while changing the molecular sieves (MS) from 3 to 4 Å greatly improved the yield, but decreased stereoselectivity. Conversely, lowering the temperature to 25 °C provided the highest stereoselectivity (*ee* 92%) with a relatively high yield (61%). The incorporated THBC moiety and bispirooxindole framework are both core structures of pharmaceutically important compounds [179].

#### 4.3.3. Other Pictet-Spengler Reactions in Cascade Sequences: Update (2011–2015)

**Scheme 52:**
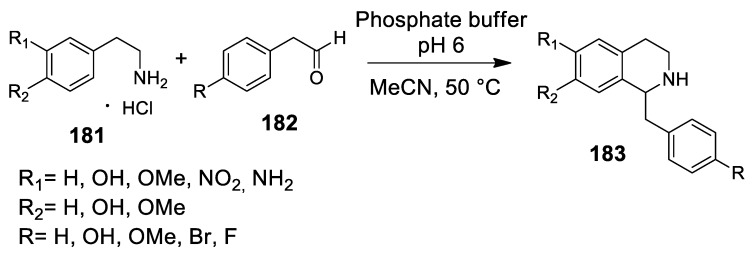
One-pot synthesis of tetrahydroisoquinoline alkaloids by phosphate buffer catalysis.

P-S cyclization attends in different manners to cascade reactions, providing frameworks that are mostly centered on THIQ or THBC motifs.

(2011) The previously cited coupling of arylethylamines **181** and arylacetaldehydes **182** to give 1-benzyl-tetrahydroisoquinoline derivatives **183** in mild reaction conditions (Scheme 52) [30].

**Scheme 53:**
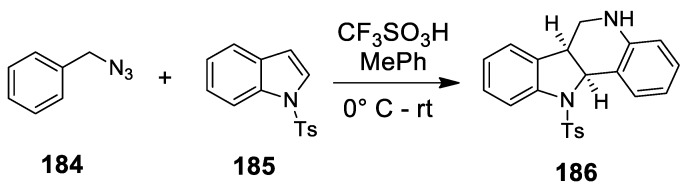
Two sequential P-S reactions in the cascade construction of tetrahydroindoloquinoline derivatives.

**Scheme 54:**
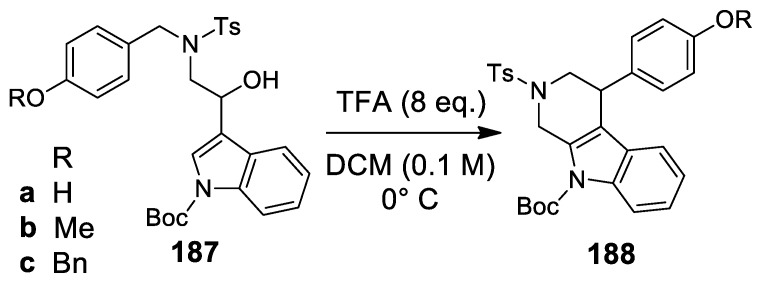
Acid-promoted unexpected skeletal rearrangement.

**Scheme 55:**
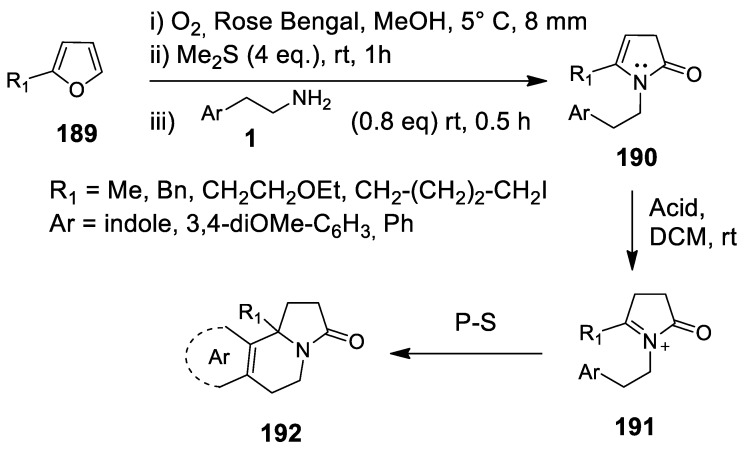
Conversion of a simple furan into the intact erythrinane skeleton.

- Inter- and intramolecular P-S cyclizations conclude cascade sequences in the mechanism invoked for the assembling of tetrahydro-5*H*-indolo[3,2-*c*]quinolines **186** through the formal [4 + 2] cycloaddition of benzyl azide **184** and *N*-protected indole **185** (Scheme 53) [180].
- A methodology (including P-S reaction) was applied in the stereodivergent synthesis of Corynantheine and Ipecac alkaloids and their unnatural analogues [66,181].

(2012) A final intramolecular P-S cyclization was invoked for the skeletal rearrangement of substrate **187** to the tryptoline derivatives **188** (Scheme 54) [182].

(2013) Singlet oxygen transforms simple furan substrates in the presence of arylethylamines into complex nitrogen-bearing aromatic poly-cycles with the structural features of important natural products, such as Erythrina alkaloids. The combination of monosubstituted furan substrates **189** with different arylethylamines **1** in TFA/DCM at room temperature triggers a cascade reaction sequence to generate in a novel way an *N*-acyliminium ion **191** precursor of tricyclic compounds **192** (Scheme 55) [183].

**Scheme 56:**
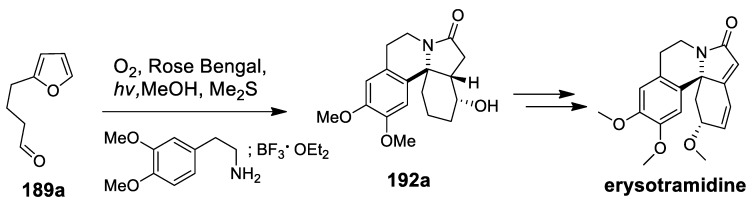
Formal synthesis of erysotramidine from simple furan derivative.

The method has been used to achieve a rapid and highly effective formal synthesis of erysotramidine, a dienoid-type member of Erythrina alkaloids, by a sequence (see Scheme 56) starting with singlet oxygen photoaddition to the furan **189a** and terminating with P-S-type aromatic substitution to give the tetracycle **192a [184]**.

**Scheme 57:**
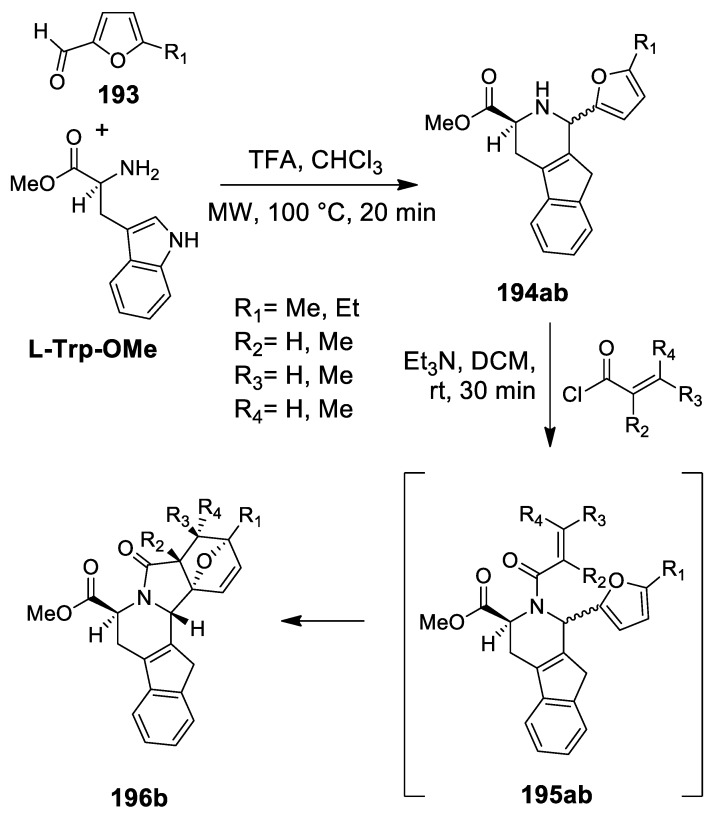
Synthesis of hexacyclic indole alkaloids via Diels-Alder reaction of *N*-acylated THBC constructed via P-S ring closure.

**Scheme 58:**
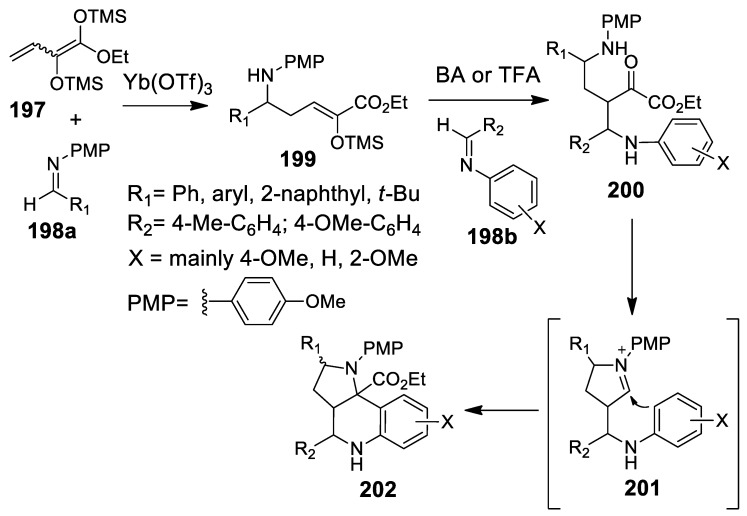
Synthesis of hexahydropyrrolo[3,2-*c*]quinolines via a sequential Mannich/Mannich/P-S procedure.

Hexacyclic indole alkaloids with a THBC motif were obtained by a stereoselective synthesis that is based on sequential P-S cyclization, *N*-acylation, and intramolecular Diels-Alder reactions. The synthetic route started with the installation of a diene motif in the THBC skeleton by a P-S reaction between 5-substituted furan 2-carbaldehydes **193** (R_1_ = Me, Et, Br) and L-tryptophan methyl ester. The resulting product **194ab** (as a mixture of *cis/trans* diastereomers) that was treated with acryloyl chlorides (DCM, Et_3_N at rt) afforded the α,β-unsaturated amides **195ab**, which spontaneously underwent [4 + 2] Diels–Alder cycloaddition to give the bridged indole alkaloids **196b** in the *exo* form, while the *cis*-isomer **195a** remained unreacted (Scheme 57) [185].

(2014) The treatment of a novel 1,2-dinucleophile (bis-silyldienediolate **197**) with two *p*-methoxyphenyl (PMP) imines (**198a** and **198b**) in a sequential Mannich/Mannich/P-S tandem process provided complex hexahydropyrrolo[3,2-*c*]quinolines (Scheme 58). The α-keto ester **200**, which were obtained after a vinylogous Mannich addition (with Yb(OTf)_3_ in dry MeCN or DME) and a second Brønsted acid-catalyzed Mannich reaction, spontaneously cyclizes within 10 min. into the pyrroloquinolines **202** (as a mixture of diastereomers, optimized by screening of the solvents and acids) (Scheme 58). Notably, being the second step triggered by the addition of the Brønsted acid, two different imines **198a** and **198b** can be employed, which substantially broadens the scope of the transformation [186].

**Scheme 59:**
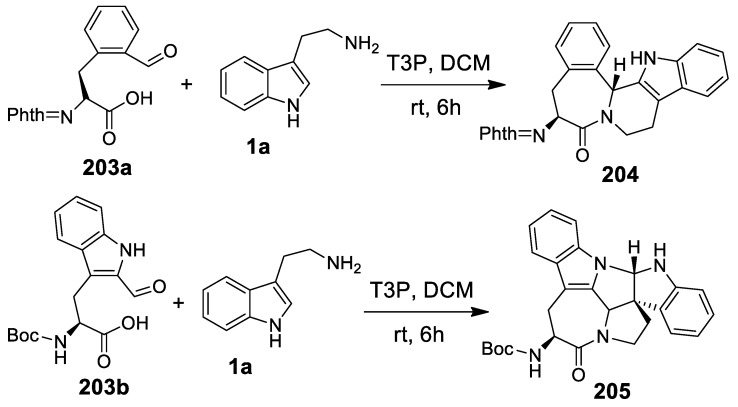
Diastereoselective synthesis of constrained 7,5 and 7,6 fused azabicycloalkanes.

The reaction between *N*-Phth-2-formyl-L-Phe-OH (**203a**) and tryptamine **1a** in the presence of propylphosphonic anhydride (T3P, 1 eq.), under stirring at RT for 6h in DCM afforded the polycyclic lactam **204** as a single *trans* diastereomer, in good yield (72%) and excellent diastereomeric excess (*de* > 98%, Scheme 59 up). By contrast, *N*-Boc-2-formyl-L-Trp-OH (**203b**) and tryptamine **1a** provided the polycyclic spirolactam **205** instead of the expected product (Scheme 59 down). No polycyclic lactam formation was observed from the condensation of keto acid **203b** and L-Trp-OMe, whereas the thiophene derivative (not shown) was obtained under optimized P-S conditions in 86% isolated yield and 98% *de* [187].

**Scheme 60:**
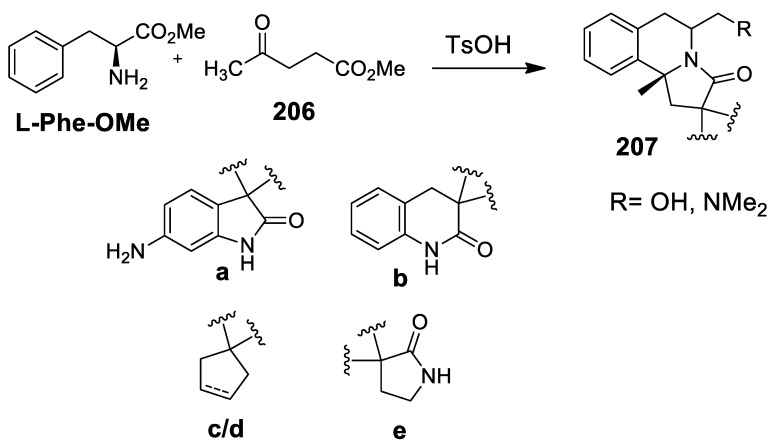
Synthesis of pyrroloisoquinoline scaffold as a platform for diversity-oriented synthesis (DOS) strategy.

The tandem P-S/lactamization of L-phenylalanine methyl ester with methyl levulinate (**206**) produced the tricyclic pyrroloisoquinoline motif **207**, which was utilized as a platform containing the pluripotent reaction site for the diversity-oriented synthesis (DOS) of a library of hybrid systems. The structural diversity was generated through the introduction on the main motif of spiro-connected privileged scaffolds, such as oxoindole (**a**), quinolone (**b**), cyclopent-ane/ene (**c/d**), and pyrrolidinone (**e**) (Scheme 60) [188].

## 5. The Ring-a Ring of Multi-Component Reactions

*Awake–awake!*

*Now come and make*

*A ring–a ring of roses*

(A.S. Stephens)

In the continuous search for an efficient synthesis of the basic skeleton of biologically active THIQs, THBCs, or polycyclic alkaloid systems embodying them, the technique of multi-component reaction (MCR) gained increasing attention from the chemists as an alternative to sequential multistep synthesis [189,190,191]. MCR chemistry enables the rapid construction of complex and diverse structures from readily accessible starting materials in a single operation under mild conditions. MCR is defined as a reaction, in which three or more starting materials combine in a single event to form a single product that contains features of all of the inputs, with the exception of condensation products, such as H_2_O, HCl, or MeOH [192]. In practical terms, the order of addition of the individual components does not matter, since multiple elements of diversity are being introduced in a single operation, regardless of the sequence in which they are added. MCRs mostly involve a number of equilibrium subreactions, which, like in a spiral frame culminate in the final step, an irreversible process, such as C—C bond formation or a rearrangement [193,194]. Several descriptive tags are regularly attached to MCRs: atom economic, the majority of the atoms of the reactants being incorporated in the products; efficient, the final product being formed in one-step instead of multiple sequential steps; convergent, several reactants combining in one reaction; endowed with very high bond-forming index (BFI), many non-hydrogen atom bonds being formed in one synthetic preparation [190,195]. Multicomponent reactions, in which all of the substrates are added at once, can also be considered to be domino processes; alternatively, MCRs can be performed by the sequential addition of reactants, without the isolation of intermediate species or a change of solvent. After the emergence of combinatorial chemistry and diversity-oriented synthesis, today MCRs play a central role in the development of modern synthetic methodology for pharmaceutical and drug discovery research [196,197].

**Scheme 61:**
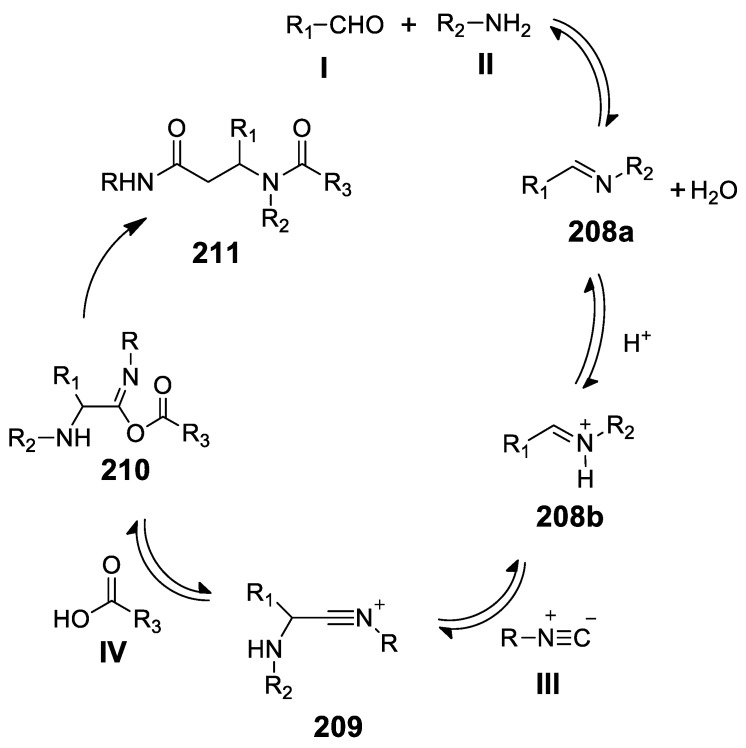
Educts in equilibrium involved in a classical Ugi-4CR reaction.

Ugi fully recognized the huge potential of the MCR methodology, who postulated that the reaction was ideally suited to probe structure-activity relationships via the synthesis of “collections of compounds”, nowadays called libraries. Since its original publication in 1959 [198], the Ugi reaction has emerged as the most well known and widely used MCR in organic synthesis. The classical Ugi four-component reaction (U-4CR) allows for simultaneous variation of four very common starting materials (educts): carbonyl compound (**I**), amine (**II**), isocyanide derivative (**III**) as a special guest and carboxylic acid (**IV**). Isocyanides (formerly known as isonitriles) are the only class of stable organic compounds with a formally bivalent carbon and a functional group fundamentally different from the others. The most synthetically important property of isocyanides is the α-addition of nucleophiles and electrophiles at the carbon atom. Scheme 61 shows a very simplified reaction mechanism with carboxylic acid as the acid component. In the first step, the oxo component (**I**) and the amine (**II**) condense to the imine **208a**, via a hydroxy aminal. The Schiff base **208a** upon proton activation (**208b**) combines with the isocyanide (**III**) to give the intermediate nitrilium ion **209**, which by addition of carboxylate (**IV**) gives intermediate **210**. Finally, the irreversible intramolecular acyl migration of **210** drives the reaction out of the ring-a-ring turning into the classic peptide-like Ugi adduct **211 [199,200,201,202,203]**.

### 5.1. Ugi Meets Pictet-Spengler

**Scheme 62:**
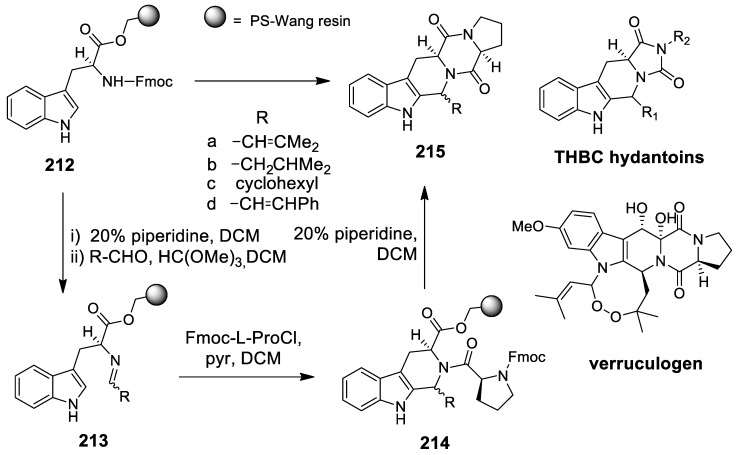
Indolyl diketopiperazines (**215a**–**d**) and THBC hydantoins synthesized by solid phase multicomponent (MCR)/P-S approach.

In earlier two-component (2C) intramolecular variations of the P-S condensation, THIQ and THBC scaffolds were prepared by the reaction of a solid-phase bound amine with a carbonyl derivative, prior to the cyclization of the incipient iminium species [204,205,206]. Successively, Wang and Ganesan added the *N*-acyliminium P-S reaction to the repertoire of multicomponent reactions, adapting a previous total synthesis of demethoxyfumitremorgin C (**215a**) [207] to the solid phase: Fmoc-protected L-tryptophan **212**, which was immobilized on polystyrene-Wang resin, was treated with senecialdehyde (R = —CH = CMe_2_) and trimethyl orthoformate to give the imine **213a**. The addition of Fmoc-L-proline chloride gave rise to *N*-acyliminium P-S cyclization to **214**, which, in turn, treated with piperidine undergoes deprotection, diketopiperazine ring closure, and concomitant resin cleavage. The natural product **215a** was obtained, together with its *trans* epimer (Scheme 62). Analogous compounds **215** were also prepared varying the aldehyde and/or replacing the proline unit by other amino acids [208]. On the other hand, Bonnet and Ganesan obtained THBC hydantoins (Scheme 62) with a similar *cis/trans* ratio by a slightly modified strategy [110]. Finally, van Loeveijin et al. reported a similar approach for the construction of a 42-membered library of fumitremorgin-type indolyl-diketopiperazines (**215**), such as verruculogen (Scheme 62) [209].

**Scheme 63:**
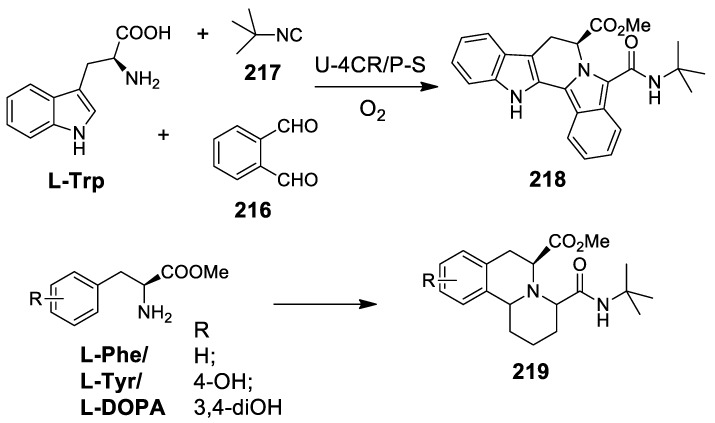
Synthesis of THBC- or THIQ-containing polycyclic scaffolds by Ugi-4CR/P-S and oxidation reactions.

The P-S-adducts finally appeared at the edge of the ring-a-ring in the framework of a substantial body of work devoted to post condensation modifications of Ugi-4CR educts **211**. Dömling and Ugi first reported the combination of Ugi and P-S reactions and included the amino acid tryptophan, phthalic aldehyde **216**, and *t*-butyl isocyanide **217** as substrates of the synthesis of complex polycyclic products containing the THIQ and THBC motifs. The non-isolable Ugi product afforded the pentacyclic compound **218** after spontaneous P-S cyclization and the following oxidation, while other aromatic amino acids, such as phenylalanine (R = H), tyrosine (R = 4-OH), and DOPA (R = 3,4-di-OH), produced tricyclic compounds, such as **219** (Scheme 63) [199].

**Scheme 64:**
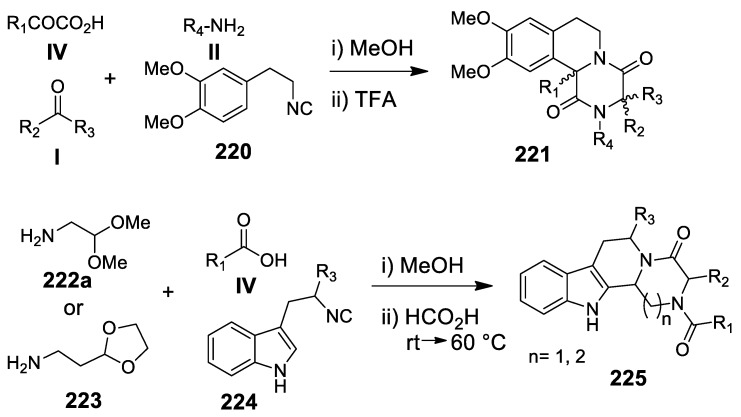
Classic version A (up) and versatile version B (down) of Ugi-4CR/P-S sequence.

The Ugi-4CR/P-S-2CR became quite popular for the rapid assembly of diverse polycyclic scaffolds. El Kaim et al. reported a classic version (A) of Ugi-4CR/P-S sequence (Scheme 64, up) [210] with the use of phenethyl isocyanide **220** for the obtainment of diketopiperazines **221**, while Dömling et al. introduced tryptophan-derived isocyanides **224** in a version (B) of Ugi/P-S reactions for the preparation of polycyclic indole alkaloid scaffolds **225** (Scheme 64, down) [211].

**Scheme 65:**
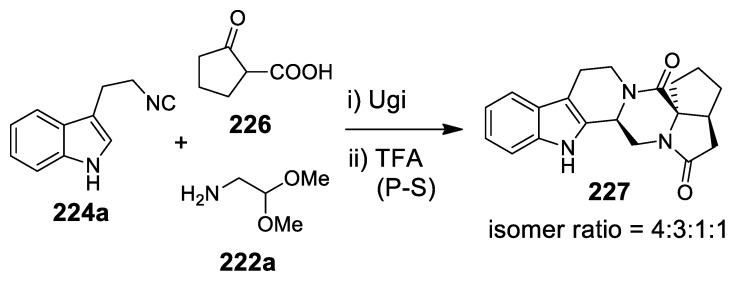
Ugi-4CR/P-S-2CR sequence for hexacyclic architecture.

Dömling and coworkers reported an efficient and flexible 2-step procedure for the synthesis of complex multicyclic indole alkaloid-type compounds **227**, which feature Ugi MCR and P-S reactions (Scheme 65). The final product is a highly complex molecule that contains six cycles in total, four heterocycles (pyrrole, piperazinone, hydropyridine, γ-lactam) and two carbocycles (benzene, cyclopentane) [212].

Other notable employments of Ugi/P-S sequence prior to 2011 can be found in the combination of tandem MAO desymmetrization/MCR/P-S cyclization for the asymmetric synthesis of alkaloid-like polycyclic compounds [213,214], as well as for the synthesis of cyanocycline A and bioxalomycin β2 [215]. The mature state and the use in the synthesis of biologically active compounds of this large group of reactions are reflected in the numerous preclinical and development drugs, for instance, almorexant (first-in-class orexin I antagonist) and retosiban (oxytocin receptor antagonist) [216,217].

### 5.2. Update of Ugi/Pictet-Spengler Combinations

**Scheme 66:**
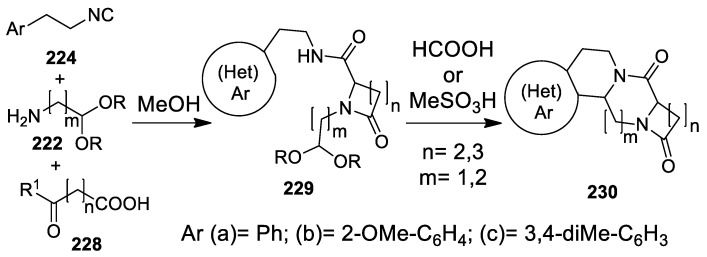
Ugi-3CR/P-S ring-closure reaction sequence for the synthesis of tetra- and pentacyclic structures.

(2011) Dömling and coworkers elaborated on their previous Ugi-3CR/P-S reaction sequence (Scheme 65 [212]), the synthesis of a small focused library of polycyclic ring systems, based on phenylethylamine-derived isocyanides **224**. All the suitable bifunctional oxocarboxylic acids **228** reacted satisfactorily with aminoaldehyde dimethyl acetal and the aryl ethyl isocyanides **224** to afford Ugi-adducts **229** with yields that range from 38 to 62% (Scheme 66). P-S reaction of intermediates **229** afforded polycyclic scaffolds **230**, in the presence of formic acid at RT for the more reactive **229c** and with methanesulfonic acid at 70 °C for the less reactive **229a** and **229b**, which also required longer reaction times [218].

**Scheme 67:**
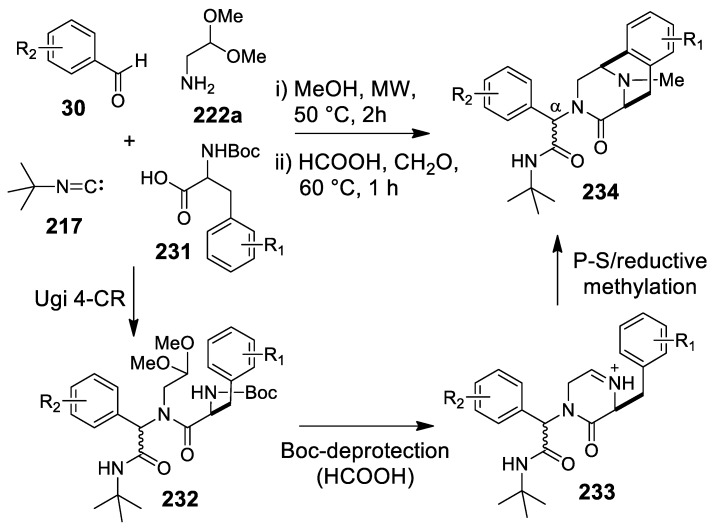
Synthesis of the piperazinohydroisoquinoline system while using a sequential Ugi/P-S/reductive methylation reaction protocol.

A sequential Ugi/P-S/reductive methylation reaction was used for the synthesis of the piperazinohydroisoquinoline ring system, starting from aminoacetaldehyde dimethyl acetal, (−)-(*S*)-*N*-Boc-3-(3,4-dimethoxyphenyl) alanine **231**, and *t*-butyl isocyanide **217**, in combination with several aldehydes **30** (Scheme 67). The Ugi-4CR/P-S-2CR reaction was completed in a straightforward manner within 1–2 h when the reaction was carried out at 50 °C while using microwave irradiation. The one-pot transformation of the Ugi-adducts **232** into the desired piperazinohydrosoquinolines **234** via iminium intermediates **233** was performed with comparable yields, simply by evaporating the solvent from the reaction flask after the completion of the Ugi reaction and then adding the reactive mixture CH_2_O/HCOOH [219].

**Figure 4:**
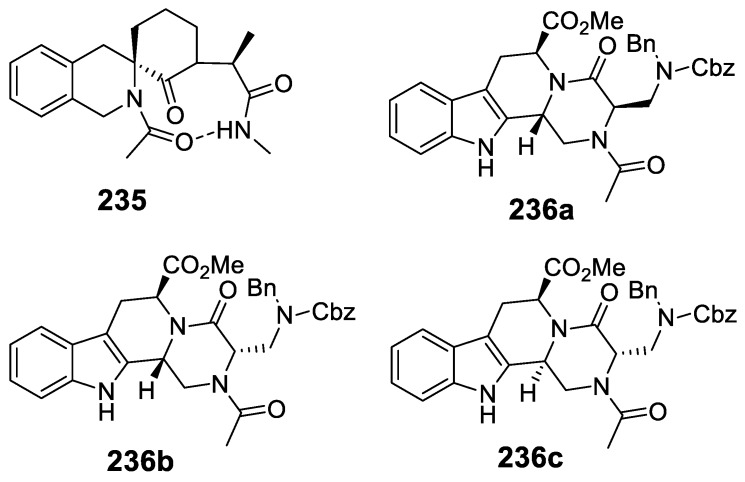
Ugi/P-S-based synthetic β-turn and γ-turn peptidomimetics.

The Lesma group carried out the synthesis of a novel Phe-Ala dipeptidomimetic **235** (Figure 4), built up on a diazaspiro-cyclic lactam core. Molecular modeling, IR, NMR, and X-ray diffraction experiments agree on the presence of a strong intramolecular hydrogen bond supporting the ability of this spiro compound to act as type II′ β-turn inducer [220].

(2012) In the framework of a program focused on the identification of new peptidomimetic of potential interest in drug discovery, Lesma and coworkers reported a two-step efficient route for the synthesis of THBC-based compounds as privileged molecular targets in the design of potential reverse turn mimics [221]. The authors applied the Ugi/P-S sequence for a rapid assembly of peptidomimetic scaffolds of type **236**. NMR and molecular modeling on the corresponding methyl carboxamide *N*-acetyl derivatives both confirmed a β-turn like conformation for the *cis*-isomer **236a** and γ-turn for the *trans*-isomers **236b**-**c** (Figure 4) [222].

**Scheme 68:**
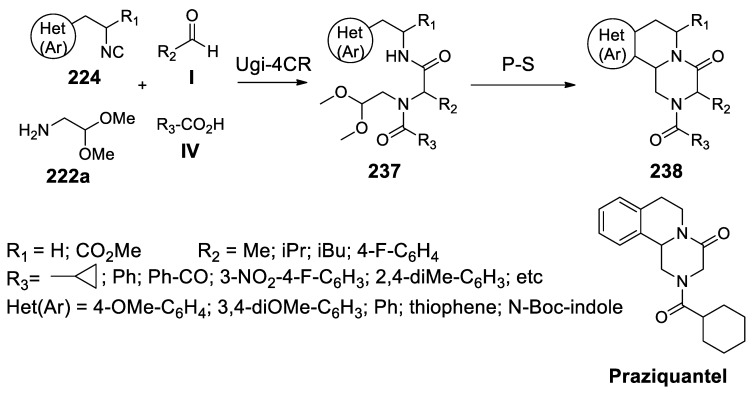
General approach Ugi-4CR/P-S for the synthesis of praziquantel analogous.

Praziquantel (PZQ) is the only effective drug for the treatment of schistosomiasis, a high volume neglected tropical disease that affects more than 200 million people worldwide. Liu et al. developed a convergent and versatile synthetic method to prepare easily accessible and highly diversified PZQ derivatives for extensive structure-activity relationship studies. The approach includes Ugi-4CR, followed by a P-S ring closure in a sequential one-pot, two-step procedure (Scheme 68). Even though the products were found to be slightly less active than the mother drug PZQ, the Ugi/P-S reaction sequence remains the shortest and scalable approach towards a future bioactivity-guided optimization of PZQ analogous [223].

**Scheme 69:**
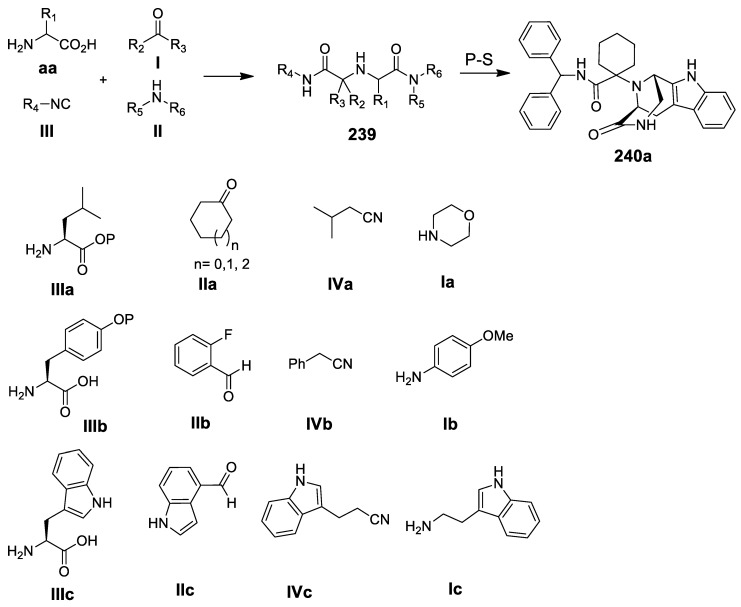
Assembly of iminodicarboxamides by a truly Ugi-4CR followed by P-S cyclization.

**Scheme 70:**
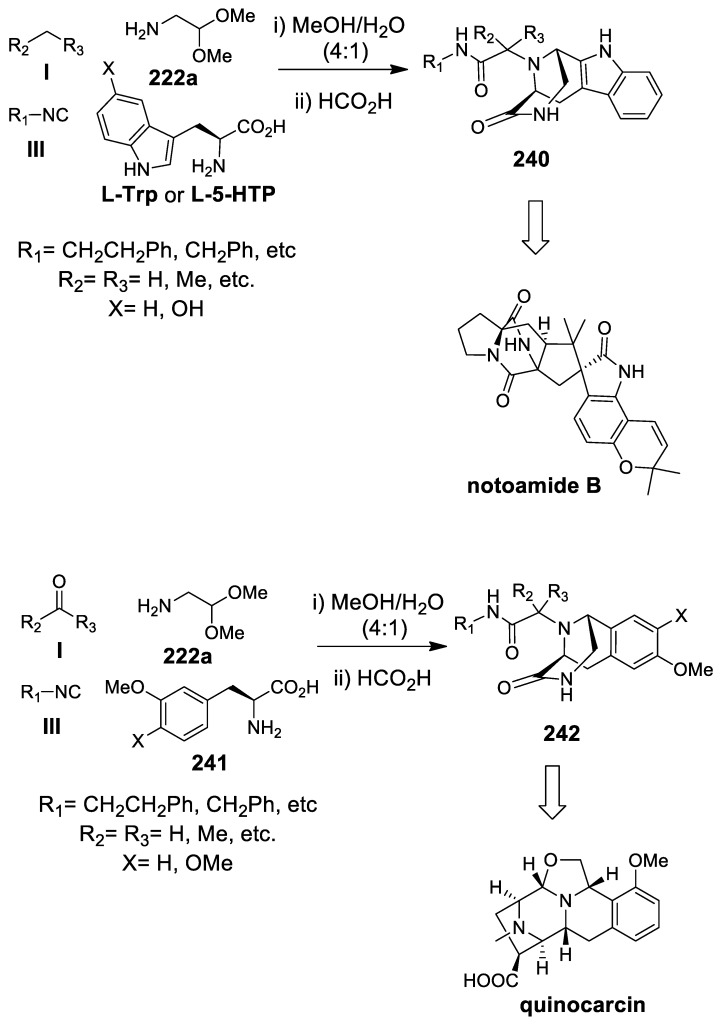
New indole and isoquinoline scaffold from P-S cyclization of Ugi-4CR adducts.

**Scheme 71:**
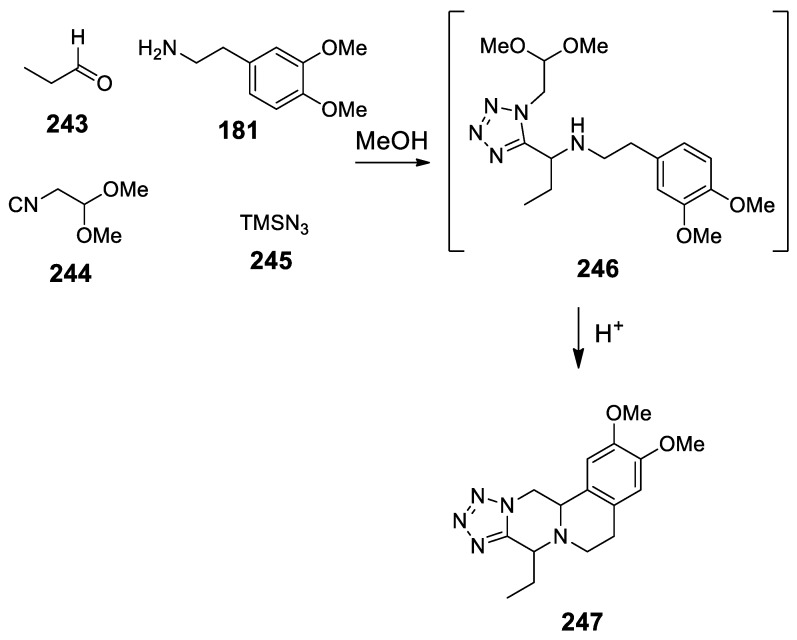
Synthesis via Ugi/P-S of 5H-tetrazoleo[1′,5′:4,5]pyrazino [2,1-*a*]isoquinoline.

A novel stereoselective Ugi-type reaction of the four highly variable starting materials α-amino acid (e.g., leucine**)**, oxo component **I** (e.g., 2-fluorobenzaldehyde), isocyanide **224** (e.g., benzyl isocyanide), and primary or secondary amine (e.g., morpholine), thus comprising a novel and true 4-CR, provided the iminodicarboxamide **239** (Scheme 69). The extensive optimization of the reaction included: the solvent, a mixture MeOH/H_2_O = 4:1, as a compromise in solubility for the different classes of starting materials; the temperature, RT instead of microwave conditions avoided the formation of undesired side products); the reaction time, three days, and the catalyst. The scope of the reaction was investigated while using representative starting material of each class. The use of bifunctional starting materials allowed for cyclizing the initially formed Ugi products, such as compound **240a**, whih was obtained by P-S reaction and deprotective cyclization [224].

(2013) The authors presented a paper containing full experimental detail on the synthesis of indole (**240**) and THIQ (**242**) derivatives that were obtained using conc. HCOOH at room temperature by the P-S reaction reported in the previous paper [224]. Notably, the cyclization was performed without purification of the initial Ugi product. The structure of the indolo annulated derivative **240b** (R_1_ = CH_2_CH_2_OCHO; R_2_ = R_3_ = Me) was confirmed by single-crystal X-ray analysis. Structures **240** and **242** can be found in the architectures of potential natural-product targets, such as the antitumor quinocarcin or the insecticidal notoamide B (Scheme 70) [225].

(2013) The Ugi reaction of four suitably components and post-condensation reactions can provide four heterocyclic scaffolds: tetrahydroimidazo[1,2-*a*]pyrazine-2,6(3*H*,5*H*)dione, pyrrolidinedione, and isoindolone systems have been yielded via post-Ugi secondary cyclization, while the unique structures of the strained tricyclic 3,9-diazabicyclo[3.3.1.]nonane skeletons **240/242** were obtained by a P-S reaction, where the required functional groups, an electron rich aromatic ring and the oxo partner, were conveniently introduced via the α-amino acid and the primary amine component, respectively. Several analogous reactions show the potential of this transformation (not shown) [226].

(2015) The authors designed novel bi- and tri-cyclic scaffolds based on the Ugi tetrazole synthesis. The reaction of propionaldehyde (**243**), 3,4-dimethoxyphenethylamine (**181**), isocyanoacetaldehyde dimethyl acetal (**244**), and trimethylsilyl azide (**245**) in MeOH at room temperature afforded after 18 h the classical Ugi product **246**, which in turn gave the P-S cyclized product **247** (67% yield) by simple treatment with methanesulfonic acid for 18 h at room temperature (Scheme 71). The scope of the methodology was examined with different oxo components and various aryl ethyl amines that produce a large range of results. Diastereomeric mixtures were obtained, but a major stereoisomer was isolated in some cases [227].

## 6. The Enzymatic Pictet-Spengler: Briefing and Update 2011–2015

*We are star-crossed;*

*cursed to walk*

*divergent paths*

(Grace, crossroad poems)

The connection of small metabolites to the gene that encodes them has sparked a renaissance in natural product research, which is primarily focused on the biosynthesis [228]. Most of the strength of P-S reaction in the field resides in the parenthood with analogous enzyme-catalyzed key-transformations in the biogenetic pathways of natural products [1,229].

Starting in the ancient world of natural products, the pathway of P-S reaction culminates in the modern technological enzymatic applications to biological systems.

### 6.1. Pictet-Spenglerases

Enzymes catalyzing the P-S condensation have been isolated from several plants and they have entered in the community, named “Pictet-Spenglerases”. These are carbon-carbon bond-forming enzymes, which join two relatively simple molecules to form a nitrogen heterocycle with excellent stereochemical control of the resulting chiral center [230].

#### 6.1.1. Biogenetic Studies

**Scheme 72:**
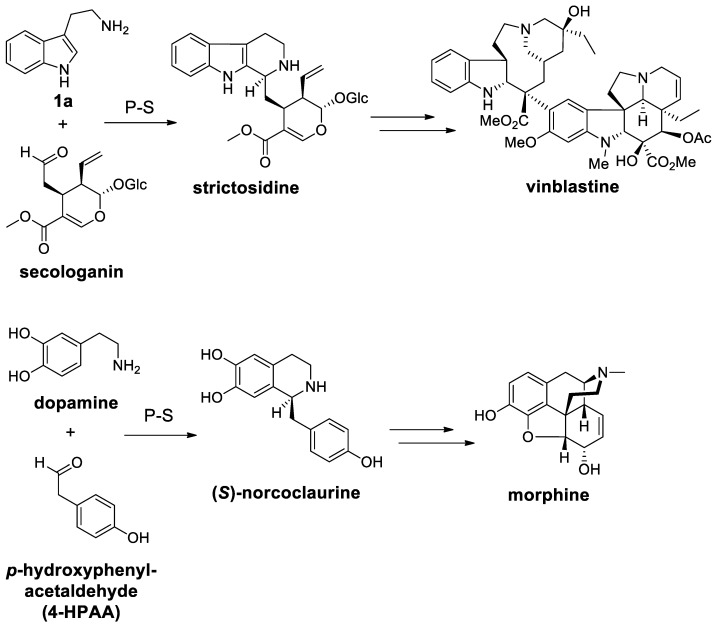
Pictet-Spenglerases at work in the biosynthesis of vinblastine and morphine.

Strictosidine synthase (STR) and norcoclaurine synthase (NCS) are the best known and studied enzymes among the Pictet-Spenglerases. By the enzymatic P-S condensation of tryptamine **1a** and an iridoid monoterpene, namely the secologanin, strictosidine synthase (EC 4.3:3:2) triggers the formation of strictosidine, the common precursor of the biosynthesis of more than 2500 monoterpene indole alkaloids of the *Apocynaceae* family (Scheme 72 up) [228,231,232,233,234,235]. STR enzyme, which was characterized in many plants, such as *Rauwolfia serpentina*, *Catharantus roseus*, and *Ophiorrhiza plumila*, only produces the 1α(*S*)-epimer (strictosidine), but not the 1β(*R*)-epimer (vincoside). However, strictosidine synthase was demonstrated to serve as precursor for tetrahydroisoquinolines (THIQs), not only with α- but also with β-configuration at C-1 chiral center [236,237].

Conversely, the enzyme norcoclaurine synthase (NCS; EC 4.2.1.78), which is isolated from *Thalictrum flavum* (*Tf*NCS), is responsible of the synthesis of benzylisoquinoline alkaloids (BIAs), which include morphine and codeine, through the precursor (*S*)-norcoclaurine (formed by the P-S condensation of dopamine and 4-hydroxyphenylacetaldehyde (4-HPAA) (Scheme 72 down) [238,239]. For instance, the anticancer candidate noscapine was obtained via this pathway [240].

An enzyme mechanism for natural substrates was developed on the basis of the *holo*TfCNS crystal structure, featuring dopamine and a non-productive aldehyde. This “HPAA-first” mechanism suggests the binding of 4-hydroxyphenylacetylaldehyde (4-HPAA) to the enzyme prior to dopamine [241,242].

**Scheme 73:**
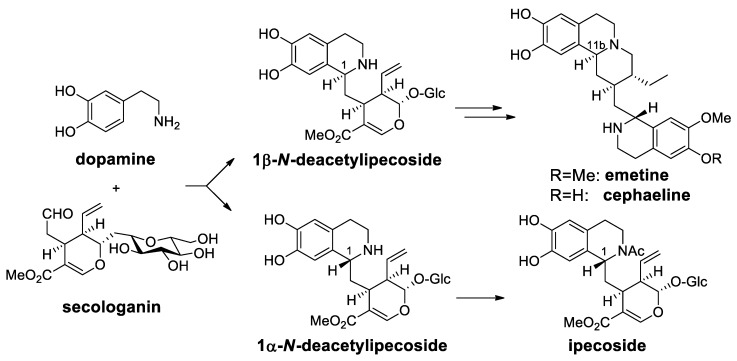
Biosynthetic pathways of Ipecac alkaloid glucosides.

Deacetylipecoside synthase (DIS) is the enzyme that catalyzes the Pictet-Spengler-like condensation of dopamine and secologanin to form two epimers, 1α-*N*-deacetylisoipecoside, the precursor of alkaloids, emetine, and cephaeline, and 1β-*N*-deacetylipecoside, which is converted to tetrahydroisoquinoline monoterpene glucosides, such as ipecoside (Scheme 73). Emetine, ipecoside, and similar natural products are named collectively Ipecac alkaloids, having been isolated from *Psychotria ipecacuanha*. Their biosynthesis has been extensively studied [243,244,245], but no new paper appeared in the 2011–2015.

#### 6.1.2. Biosynthesis: Update 2011–2015

**Scheme 74:**
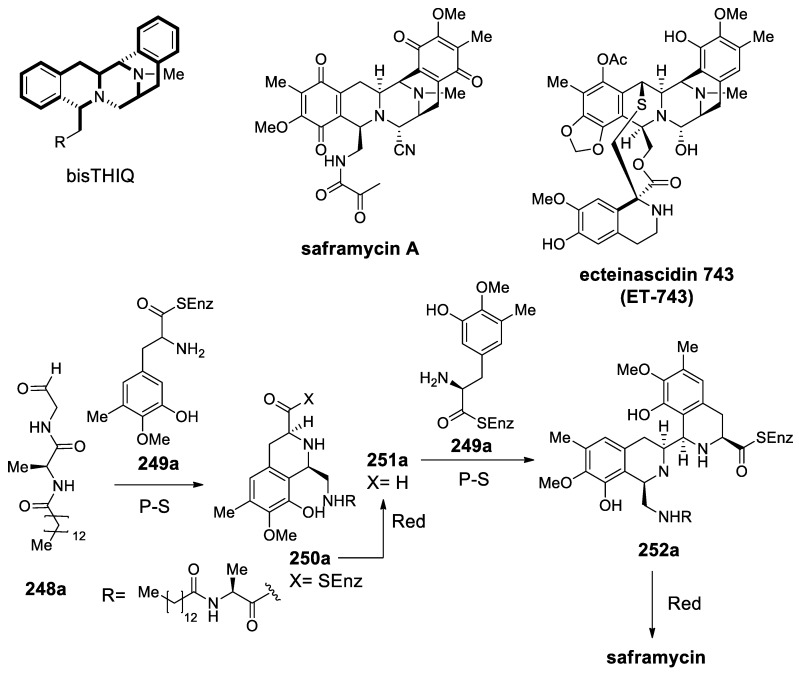
Schematic representation of saframycin biosynthetic pathway.

**Scheme 75:**
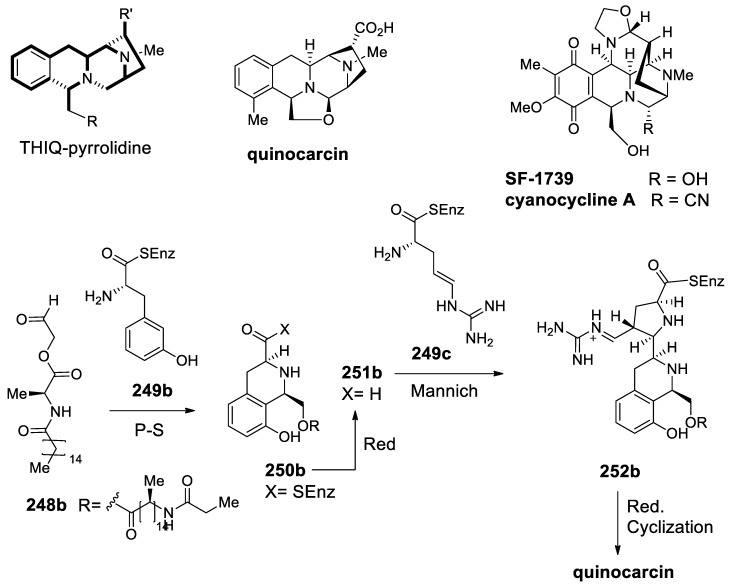
Schematic representation of quinocarcin core assembly.

(2012) Oikawa and coworkers demonstrated that the core scaffold of microbial THIQ antitumor antibiotic of the type bis-THIQ, such as saframycin A and ecteinascidin, is biosynthesized by a nonribosomal peptide synthetase (NRPS), which catalyzes a highly unusual seven step transformation while using a simple fatty acyl-dipeptidyl coenzyme A (**248a**) and a modified tyrosine analogue (**249a**). The authors proposed a biosynthetic pathway that involves multiple reductions (Red) of thioester intermediates (**250a** and **252a**) and two rounds of P-S cyclization (Scheme 74) [246,247]. The group described a protocol for the biochemical characterization of saframycin NRPS SfmC and showed that the P-S reaction relies heavily on the chain length of the cryptic long acyl chain in the peptide substrates [248].

(2013) The Oikawa group reported then the identification of the biosynthetic gene clusters of quinocarcin and the antitumor antibiotic SF-1739, which share a common type II tetracyclic tetrahydroisoquinoline (THIQ)-pyrrolidine core scaffold. The authors proposed a reaction pathway for the construction of the quinocarcin core scaffold (sketched in Scheme 75), with similar protagonists as for saframycin and involving one P-S and one intramolecular Mannich reaction [249].

**Scheme 76:**
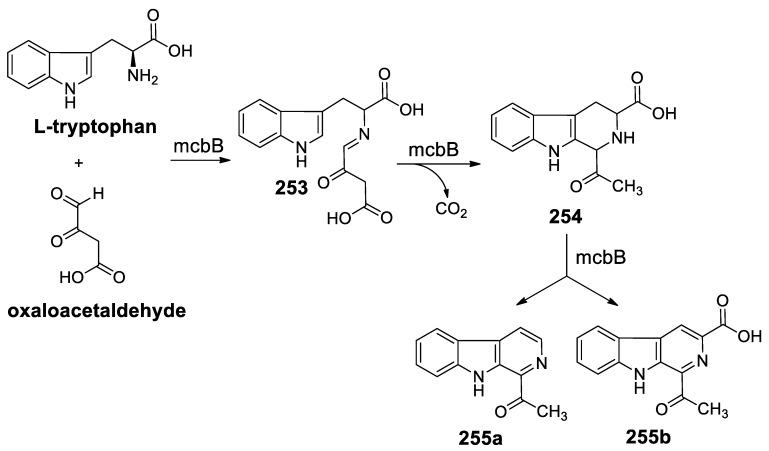
Marinacarbolines biosynthetic pathway.

Ju and coworkers identified three genes *mcbA*, *mcbB*, and *mcbC* as the solely responsible for scaffold construction of marinacarbolines (MCBs), 1-acetyl-β-carboline (**255a**), and 1-acetyl-3-carboxy-β-carboline (**255b**), which were isolated from *Marinactispora thermotolerans*. In particular, *mcbB* was proposed to be a multifunctional enzyme that catalyzes the P-S reaction of tryptophan and oxaloacetaldehyde for the assembly of the tetrahydro-β-carboline (THBC) **254** skeleton, followed by decarboxylation and C-ring oxidation to β-carbolines (Scheme 76) [250].

Abe et al. confirmed that *mcbB* indeed catalyzes the PS condensation as well as the decarboxylation and oxidation reactions by crystallographic studies and biochemical characterization. The resolution of the crystal structure of *mcbB* complexed with tryptophan revealed a totally different structure from those of other P-S cyclization catalyzing enzymes from plants, such as strictosidine synthase or norcoclaurine synthase [251].

(2014) The crystal structure of *mcbB* was also analyzed and its catalytic mechanism discussed by another group [252].

#### 6.1.3. Biocatalysis

Biotechnological approaches are gaining importance for the production of alkaloids that cannot be isolated from their natural sources in quantities that were comparable with the demand of modern medicine.

Recent advances in metabolic engineering have enabled the tailored production of plant secondary metabolites in microorganisms. A Japanese group, using selected enzyme to construct a tailor-made biosynthetic pathway, being produced the plant benzylisoquinoline alkaloid, (*S*)-reticuline (*vide infra*: Scheme 76) with a yield of 46.0 mg/L culture medium (*Escherichia coli* fermentation system) [253]. Analogously, O’Connor and coworkers suppressed tryptamine biosynthesis in *Catharanthus roseus* hairy root cultures, introduced an unnatural tryptamine analog to the silenced plant cells, and obtained a variety of novel products [254,255].

Enzymatic catalysis has been proven to be useful for a number of synthetic biotransformations [256,257], although natural product biosynthetic enzymes often have narrow substrate scope that limits their use as biocatalysts. Pictet-Spenglerases are expected to produce nitrogen heterocycles with excellent stereochemical control of the resulting chiral center. For instance, strictosidine synthase from *Ophiorrhiza pumila* can utilize a range of simple achiral aldehydes and substituted tryptamines to form highly enantioenriched (*ee* > 98%) THBCs via the P-S reaction. These findings represent the first example of aldehyde substrate promiscuity in the strictosidine synthase family of enzymes [258].

**Scheme 77:**
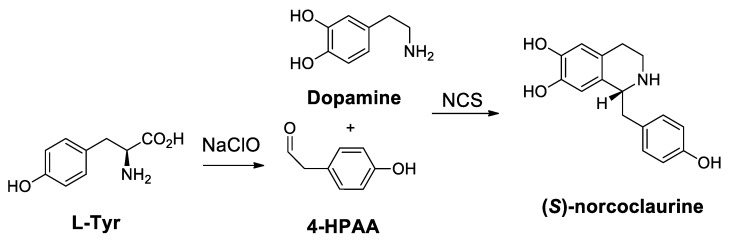
Combined chemical and enzymatic synthesis of norcoclaurine.

A recombinant norcoclaurine synthase (NCS) from *E. coli* was used to prepare (*S*)-norcoclaurine (R = H), starting from tyrosine and dopamine (R = H) as substrates in a one-pot, two-step process. Tyrosine was first chemically decarboxylated by stoichiometric amount of NaClO to generate the aldehyde species (4-HPAA), to which the enzyme and tyrosine were added (Scheme 77). The optimized process afforded (*S*)-norcoclaurine (R = H; *ee* 93%) in 81% yield and allowed for the recycling of the enzyme [259].

Successively, the same group achieved the fully enzymatic asymmetric synthesis of substituted tetrahydroisoquinolines analogs of norcoclaurine in two steps, starting from dopamine and a set of amine substrates by an oxidation performed with a diamine oxidase from plant *Lathyrus cicero* L., followed by the P-S reaction being catalyzed by the recombinant NCS from *Thalictrum flavum*. In the first step, various aliphatic and aromatic amines were transformed into the corresponding aldehydes by the broad specificity of diamine oxidase enzyme, while the second step afforded chiral substituted THIQs in good yield [260].

#### 6.1.4. Biotransformations: Update 2011–2015

**Scheme 78:**
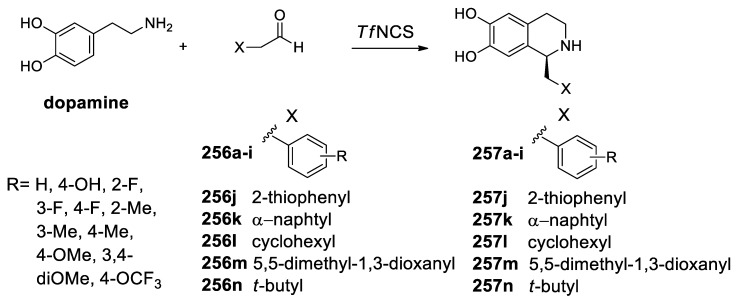
Aldehyde variability of *Thalictrum flavum* (*Tf*NCS) in the synthesis of substituted THIQs.

(2011) Pictet-Spenglerases (P-Sases) are highlighted among the enzymes that have been employed for C-C bond forming reaction on a preparative scale [261]. In concert with Oikawa model for saframycin [246], the presumed activity of a P-Sase was invoked in the biosynthetic pathway for anticancer agent ET-743 [262].

(2012) O’Connor and coworkers described the substrate scope and limitations of a NCS from *Thalictrum flavum* (*Tf*NCS). Nineteen aldehyde analogs **256a**–**n**, which were synthesized or commercially available, were treated (at 1 mM concentration) with dopamine and *Tf*NCS (300 µM) in TRIS buffer (100 µM, pH 7) to give the corresponding norcoclaurine derivatives **257** in enantioselective fashion (*S*-form) (Scheme 78) [263]. Including for each reaction inactive (boiled) enzyme controlled the enzymatic background. As a result, *Tf*NCS proved to have exceptionally broad aldehyde substrate specificity, turning over aldehydes **256a**–**n**. Only the THIQs corresponding to (the small) formaldehyde (HCHO) and acetaldehyde (CH_3_CHO), as well as benzaldehyde (C_6_H_5_CHO), could not be detected. By contrast, NCS showed a strict requirement for the amine substrate, which is dopamine. In fact, neither tryptamine, which is the natural substrate of the enzyme in the plant, nor commercially available 3,4-disubstituted phenylethylamines (not shown), provided any product with native 4-HPAA. This substrate specificity is consistent with earlier studies [238,239] that require the amine substrate to contain a hydroxy group at the C-3 position of the aromatic ring. In conclusion, these findings revealed that NCS could be used as a catalyst to yield a variety of substituted THIQs.

Pesnot et al. investigated the versatility and potential of a NCS from *Coptis japonica* (*Cj*NCS2), together with the development and application of a novel fluorescence-based high-throughput assay using several amines/aldehydes. The tetrahydroisoquinolines were formed as the (1*S*)-isomer in 95% *ee*. The exceptional tolerance of NCS towards aldehyde substrates was further supported by the proposed mechanism, in which the aldehydes protrude out of the enzymatic pocket [264].

**Scheme 79:**
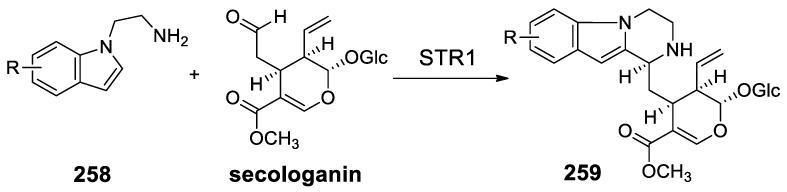
Enzymatic synthesis of the novel piperazino[1,2-*a*]indole scaffold.

**Scheme 80:**
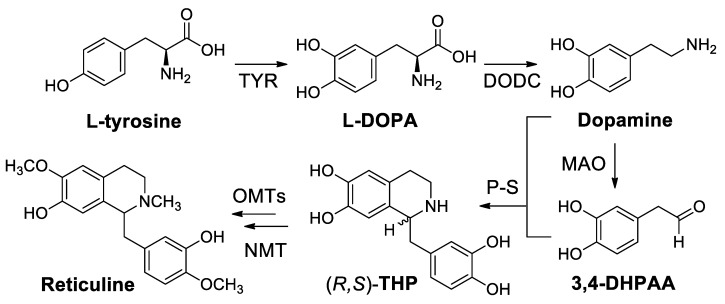
(*R*,*S*)-Tetrahydropapaveroline (THP) synthetic pathway constructed in engineered *E. coli* strains.

A novel function of strictosidine synthase (STR1) allowed, for the first time, a simple enzymatic synthesis of the strictosidine analogues **259**, harboring the piperazino[1,2-*a*]indole (PI) scaffold, starting from secologanin and the novel indole-like amine **258** (Scheme 79). STR1 provided exclusively access to products **259** and can generate by chemoenzymatic approach libraries of a novel class of alkaloids with potentially new biological activities [265].

(2014) Nakagawa et al. developed a production system of (*R*,*S*)-tetrahydropapaveroline (THP) by altering the reticuline synthetic pathway [253] that was previously constructed while using *E. coli*. L-tyrosine (obtained from glycerol via a tyrosine over-producing pathway) is oxidized by tyrosinase to L-DOPA, which in turn is transformed into dopamine by DOPA decarboxylase (DODC). Finally MAO oxidizes dopamine to 3,4-DHPAA and these last two compounds are spontaneously converted to (*R*,*S*)-THP through a non-enzymatic P-S reaction (Scheme 80) [266]. In the synthetic pathway from glycerol to tyrosine to 3,4-DHPAA, previously reported [254], NCS was used instead for THP synthesis.

Maresh et al. reported a convenient method for oxidative decarboxylation of α-amino acids and extended the enantioselective enzymatic synthesis of (*S*)-norcoclaurine (R = H, Scheme 72 [259,260]) to halogenated (R = Cl, Br, I) high purity derivatives. Phenylalanine and tryptophan were also successfully converted in the corresponding P-S products (not shown) [267]. 

Nishihachijo et al. showed that NCS is a promising catalyst for synthesizing non-natural, optically active THIQs. The authors examined the aldehyde substrate specificity of a NCS from *Coptis japonica* expressed in *E. coli*, by synthesizing 6,7-dihydroxy-1-phenethyl- and 6,7-dihydroxy-1-propyl-1,2,3,4-tetrahydroisoquinolines. The two P-S products were obtained in yield of 86.0 and 99.6%, and in *ee* of 95.3 and 98.0%, respectively [268].

(2015) Stöckigt and coworkers updated strategies and methods for exploring the applicability of STR to the formation of new alkaloids with unusual substitution pattern or (even) with novel scaffold while taking strictosidine synthase from *Rauwolfia serpentina* (*Rs*STR) and *Catharanthus roseus* (*Cr*STR) as representative models. The authors introduced the latest released complex structures of *Rs*STR with new substrates. The examples provided in the article pave the way to the construction of novel alkaloid libraries by chemoenzymatic approaches [269]. 

Ward et al. described and assessed two different mechanisms of NCS activity: The “HPAA-first” mechanism (based on the *holo* X-ray crystal structure [239]) and the “dopamine-first” mechanism. The authors observed novel kinetic parameters that show NCS to operate with low catalytic efficiency. The amino acid substitution pattern L76A, which was located in the proposed “dopamine-first” aldehyde binding site, resulted in a modification of the aldehyde activity profile, strongly supporting the mechanism in question [270].

### 6.2. Chemistry and biology of Pictet-Spengler Reaction

Started in the old world of natural products, the P-S reaction pathway culminates in the modern technological applications of enzymes.

Aldehyde- and ketone-functionalized proteins are appealing substrates for the development of chemically modified biotherapeutics and protein-based materials.

The close tie of P-S reaction with biology materializes in the iso-Pictet-Spengler reaction as the basis of a bond that connects the orthogonal bioconjugation of a small molecule with a large protein.

#### 6.2.1. Iso-Pictet-Spengler

Natural and synthetic compounds containing the THBC framework are endowed with an extraordinary range of biological activity [271]. The closely related tetrahydro-γ-carboline (THGC) scaffold is unknown among natural product structures, however, holds considerable potential as a template for drug discovery [272,273].

**Scheme 81:**
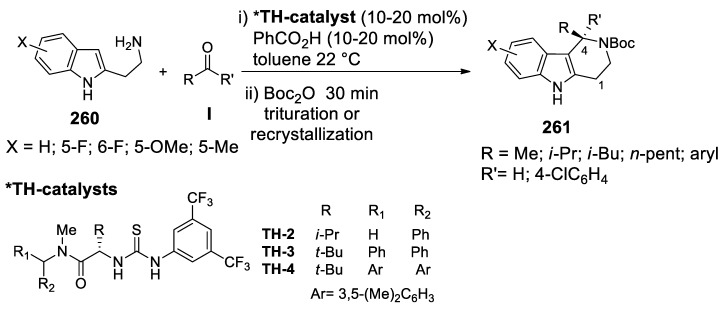
Thiourea/benzoic acid-catalyzed enantioselective iso-Pictet-Spengler reaction.

**Scheme 82:**
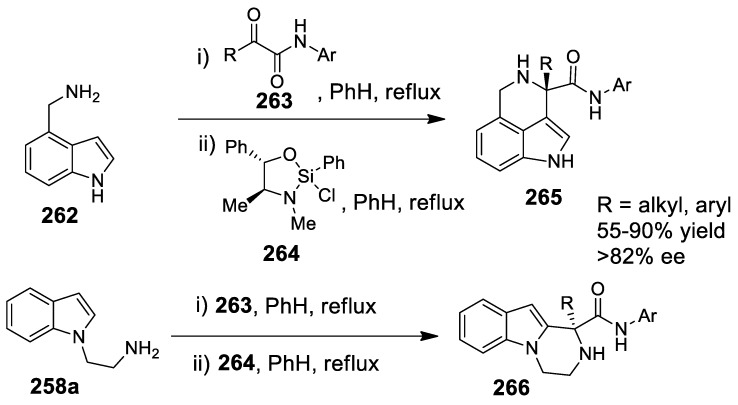
Enantioselective iso-Pictet-Spengler reaction for indole-based core structures.

Klausen and Jacobsen reported that chiral thiourea derivatives in combination with benzoic acid promote catalytic asymmetric iso-P-S reactions of electronically and sterically diverse imines, providing unprotected THBCs in high *ee* and yield [274].

(2011) Through an approach founded on the above findings, Jacobsen and coworkers described a straightforward and direct route to enantiomerically enriched THGCs **261** via the one-pot condensation/cyclization of 2-substituted-indolyl-ethylamines (isotryptamines) **260** and aldehydes or ketones **I** (Scheme 81). In a reaction defined “iso-Pictet-Spengler”, chiral thioureas **TH-2, TH-3**, or **TH-4** (20 mol%) (see Scheme 81) and benzoic acid PhCO_2_H (10–20 mol%) were found as the more effective cocatalysts and gave the THGC **261a** (X = H, R = *i*-Pr; R′ = H) with a 97% yield and 95% *ee*. [275]. Ketone substrates **I** were also successfully applied to the iso-P-S protocol. The authors explored also whether the THGC framework might undergo analogous transformations into structurally and stereochemically complex alkaloid scaffolds. They targeted the synthesis of the spirocyclic oxindole **261b** (X = H; R = Me; R′ = 4-ClC_6_H_4_) [276].

(2012) Highly enantioselective iso-P-S reactions concerned the condensation of either (1*H*-indol-4-yl)methanamine **262** or 2(1*H*-indol-1-yl)ethanamine **258a** with a variety of α-keto-amides **263**, followed by the addition of a chiral silicon Lewis acid **264**. The reaction, in a modified P-S-like procedure, provided access to 3,3-disubstituted-1,3,4,5-tetrahydropyrrolo[4,3,2-*de*]isoquinolines **265** and 1,1-disubstituted-1,3,4,5-tetrahydropyrazino[1,2-*a*]indoles **266**, respectively (Scheme 82) [277].

**Scheme 83:**
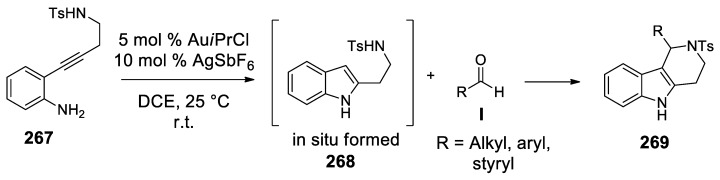
One-pot domino cycloisomerization/iso-Pictet-Spengler reaction.

Conversely the substrate **268** was prepared by a gold-catalyzed domino cycloisomerization of 2(4-tosylaminobut-1-yn-1-yl)aniline **267** and then afforded with aldehydes **I** the corresponding 1-aryl-*N-*tosyl-2,3,4,5-tetrapyrido[4,3*b*]indole **216** (Scheme 83) [275].

**Scheme 84:**
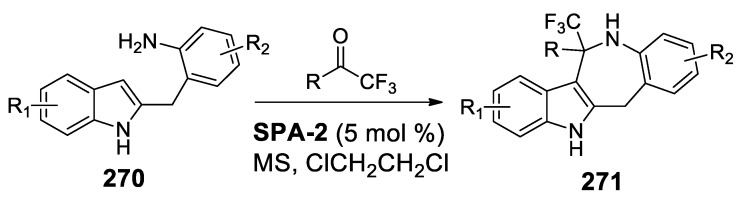
Synthesis of benzazepinoindoles via organocatalysis.

C2-linked *o*-aminobenzylindoles **217** give with trifluoromethylketones, in the presence of chiral spirocyclic phosphoric acids (**SPA-2**, see Scheme 33), optically active benzazepinoindoles **218**, bearing trifluoromethylated quaternary stereocenters (Scheme 84) [278].

#### 6.2.2. Orthogonal Bioconjugation

**Scheme 85:**
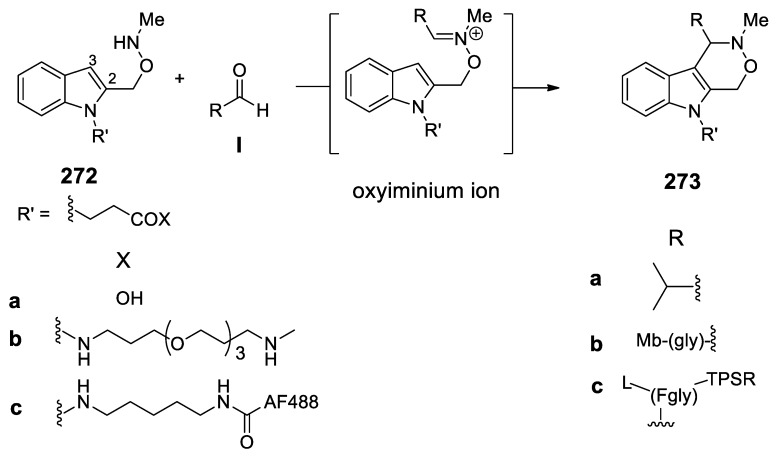
Synthesis of hydrolytically stable conjugates with glyoxyl- and formylglycine-modified proteins through a reactive oxyminium ion intermediate.

The bioorthogonal chemical reactions are the key for selective modifications of biological species and they involve the creation of non-biological molecules that exert an effect on or reveal new information about biological systems. The bioorthogonal ligation between a biomolecule and a reactive partner does not perturb other chemical functionality naturally found within the cell system [279]. Recently, McKay and Finn provided an update on the last developments in the selective chemical modification of biological molecules, the so-called bioorthogonal chemistry, and analyzed strategies and applications [280]. Aldehydes and ketones were among the functionalities amenable to incorporation into biomolecules for their synthetic accessibility and small size. Several chemical, enzymatic, and genetic methods have been developed to introduce aldehydes and ketones into protein sites specifically [279]. Historically, oximes and hydrazones have been used for ligation to carbonyls, which, as mild electrophiles are typically conjugated with α-effect nucleophiles, such as substituted hydrazines or alkoxyamines. However, the resulting C = N linkages are susceptible to hydrolysis under physiologically relevant conditions [279,280]. Moreover, oxime formation requires acidic conditions (pH 4.5) to proceed at any appreciable rate, while prolonged exposure to acid might damage sensitive biomolecules. A C-C bond was taken in consideration and the classic P-S cyclization was applied to the *N*-terminal labeling of horse heart myoglobin to overcome the instability of the oxime constructs [281,282,283], but to avoid harsh acidic conditions, which were not consistent with protein bioconjugation, the used the incubation of the protein and tryptophan or tryptamine in phosphate buffer (pH 6.5) at 37 °C for 18 h as reaction conditions.

(2013) Bertozzi and coworkers introduced a P-S ligation to prepare hydrolytically stable conjugates with glyoxyl- and formylglycine-modified proteins, including a monoclonal antibody [281]. The canonical aliphatic amine was replaced with alkoxyamine since kinetic studies of the classic P-S reaction suggested the formation of the iminium ion as the rate-limiting step. In addition, the functionality was moved to the 2-position, leaving the more nucleophilic 3-position to engage in the electrophilic substitution, such as in an “iso-Pictet-Spengler” reaction [276]. Finally, the aminooxy substituent was methylated to provide a more reactive oxyminium ion intermediate [281,282,283]. Compound **272a** (R′ = CH_2_CH_2_CO_2_H) was prepared in few steps, starting from indole-2-methanol, and culminating in the rapid formation under acidic conditions of dihydroxy-β-oxa-γ-carboline **273a**, a product that was hydrolytically more stable than the model oxime (Scheme 85). Compounds **273b** and **273c** were prepared to evaluate the iso-P-S ligation as a means to label aldehyde-functionalized proteins. The first one was obtained by treatment of Teoc protected **272a** with amino-poly (ethylene glycol)- functionalized biotin, followed by deprotection with CsF, to give **272b**, and by ligation to the *N*-terminal glyoxyl moiety of horse heart myoglobin (glyoxyl Mb). Analogously, the indole **272c** was synthesized by a similar sequence of coupling **272a** with Alexa Fluor 488 (AF488), while the oxacarboline **273c** was obtained by ligation with the formylglicine-functionalized C-terminus of the six-residue peptide sequence LCTPSR of maltose-binding protein (FGly-MBP). The studies put in evidence that P-S ligation might enhance the metabolic, enzymatic, and chemical functionalization of proteins and other biomolecules.

(2013) Rabuka and coworkers introduced a new reaction, the hydrazino-Pictet-Spengler (HIPS) ligation, which proceeds quickly around neutral pH and allows for one-step labeling of aldehyde-functionalized proteins under mild conditions. The HIPS ligation product is very stable (>5 days) in human plasma when compared to an oxime –linked conjugate (~1 day) [283].

(2014) Hydrazino-iso-Pictet-Spengler (HIPS) chemistry was successfully employed to prepare antibody-drug-conjugates (ADCs) [284,285].

(2015) The site-specific HIPS ligation was also used for labeling covalently proteins with a fluorophore [286].

A paper from Kudirka and Rabuka reviews the advance in site-specific ADCs for cancer therapy and highlights the chemistry of the reaction, dubbed iso-Pictet-Spengler ligation [287].

#### 6.2.3. Unnatural Compounds from Fungal Pictet-Spengler Biosynthesis

**Figure 5:**
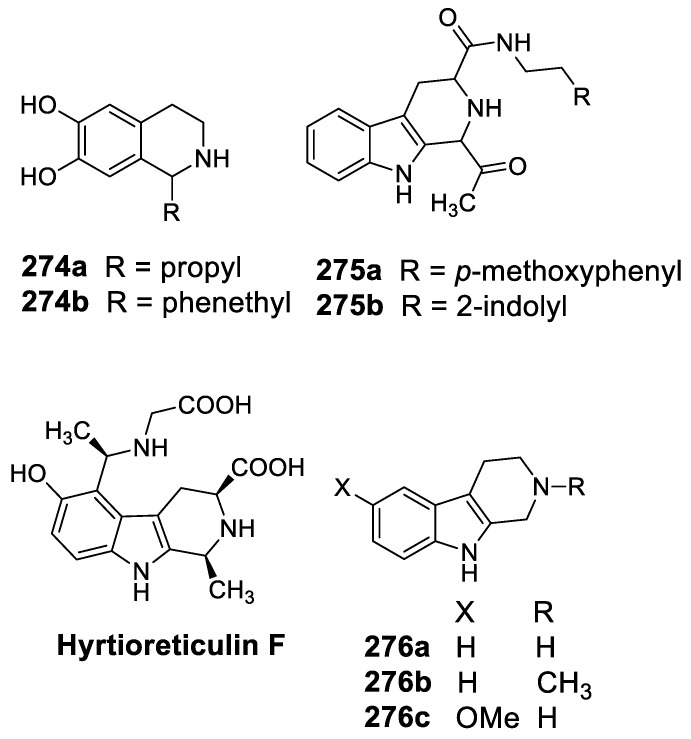
Piperidine ring-containing secondary metabolites from a variety of sources.

The P-S reaction contributes greatly to framework diversification of important alkaloids by forming a piperidine ring condensed to aromatic ring or indole moiety with plant-derived Pictet-Spenglerases. Piperidine ring-containing secondary metabolites have also been found in bacteria (e.g., THIQs **274** from *Streptomyces lavendulae* [288] and THBCs **275** from *Marinactinospora thermotolerans* [250]), in sponges (e.g., marine natural product hyrtioreticulin F from the sponge *Hirtios reticulatis* [289]) and animals (e.g., THBC derivatives **276** from rat brain [290]) (resumed in Figure 5) and a Pictet-Spenglerase has been presumed to be involved in their biosynthetic pathways.

Fungi, especially the *Ascomycota* genus, have been reported as prolific producers of alkaloids containing one or more indole/indoline moieties [291], mostly endowed with potent biological activities [292]. By time nothing was known regardinf the P-S reaction in the fungal kingdom. Only more recently the availability of fungal genome sequences has significantly accelerated the identification of genes involved in the biosynthesis of secondary metabolites from fungi [293,294].

**Scheme 86:**
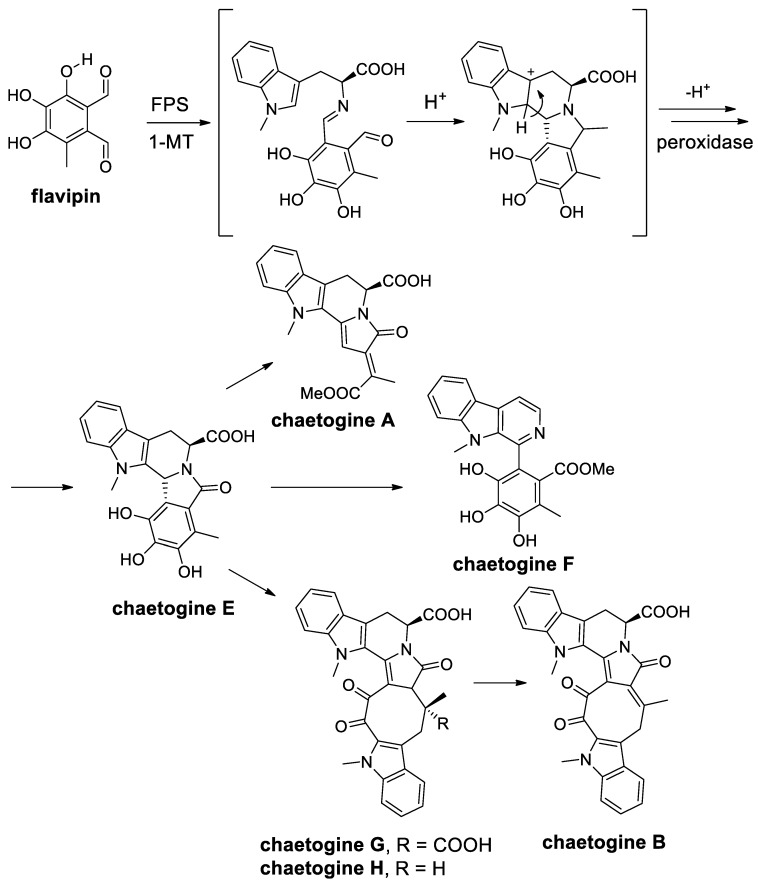
Unnatural products (with new scaffold) obtained from 1-MT activated silent FPS gene of Chaetonium globosum 1C51.

Tang and coworkers revealed that the different strategies to incorporate and derivatize indole moiety in pathways of fungal alkaloids are based on building blocks, such as L-tryptophan and the related 4-dimethylallyl-tryptophan (4-DMAT, by prenylation) and trypamine (by decarboxylation). However, biochemical evidences that confirm the direct incorporation of tryptamine as a precursor have not been reported [295]. Most, if not all, *Chaetonium* fungi in the Chaetomiaceae family produce L-tryptophan-derived alkaloid, but no P-S reaction-based secondary metabolite has been detected [296,297,298].

(2014) A comparative genomic analysis has clarified that *C. globosum* does have a fungal Pictet-Spengler (FPS) gene, which remains silent or poorly activated in laboratory cultivations. Therefore, a *C. globosum* IC5I strain was adopted to test for the activation of its “unworking” P-S reaction-based biosynthetic machinery, and 1-methyl-L-tryptophan (1-MT) was demonstrated to be able to up-regulate the FPS expression and condense with the fungal aldehyde flavipin (3,4,5-trihydroxy-6-methylphthalaldehyde) to unexpectedly form a family of skeletally unprecedented alkaloids (Scheme 86), trivially named chaetoglines A–H. Chaetogline B and F have been found to have antibacterial activity comparable to that of tinidazole (a coassayed drug prescribed in clinic for bacterial infections) against pathogenic anaerobes *Veillonella parvula*, *Bacteroides vulgatus*, *Streptococcus* sp., and *Peptostreptococcus* sp., whereas chaetogline F was also shown to be a potent inhibitor of acetyl-cholinesterase (AChE) [299].

**Scheme 87:**
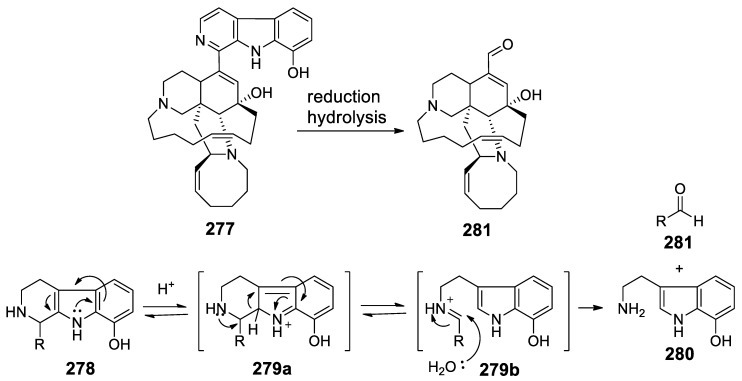
Enzymatic retro-Pictet-Spengler of fungi with manzamine alkaloids and the proposed mechanism.

Kasanah and coworkers studied the in vitro activity of the β-carboline-containing manzamine alkaloids against *Fusarium solani*, *Fusarium oxysporum,* and *Fusarium proliferatum* [300]. The data of their bioassays demonstrated that *Fusarium* spp were resistant to the manzamine alkaloids, because the fungi were able to transform manzamines via hydrolysis, reduction, and a retro-Pictet-Spengler. A pathway that involves the reverse catalytic ring opening and the hydrolysis of the iminium group, namely a retro-Pictet-Spengler reaction, was proposed for the mechanism of the acid catalyzed epimerization of reserpine [301]. A retro-P-S pathway was also suggested for the *cis* to *trans* epimerization of 1,2,3-trisubstituted-1,2,3,4-tetrahydro-β-carbolines, but it was ruled out on the basis of kinetic data [302]. According to the mechanism proposed for the activity of *Fusarium solani* on manzamine F, the THBC **278**, provided by reduction of the metabolite 8-hydroxymanzamine A **277** derived from fungal metabolism of manzamine F, gives by protonation the intermediate **279a**, in equilibrium with the open form **279b**, which finally hydrolyzes to the 7-hydroxytryptamine **280** and the aldehyde **281** (Scheme 87) [300].

## 7. Conclusions: Drawing a Veil over Act II

In the five years following the centenary birthday of P-S cyclization, a mess of paper demonstrated that the reaction did not exit the stage, but came up again on the limelight with new features. In the *lustro* the chameleonic transformation maintained the role of a protagonist, in spite of a fierce competition of variant and complementary reactions. But the history of the venerable reaction is not yet complete fulfilled.

**Scheme 88:**
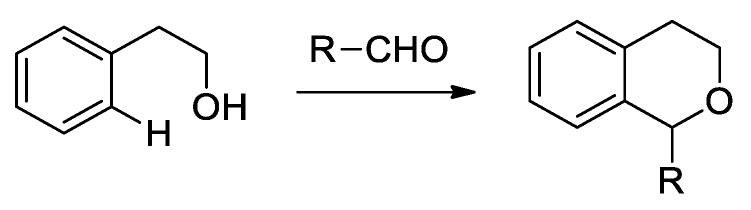
Sketched Oxa-Pictet-Spengler.

**Scheme 89:**
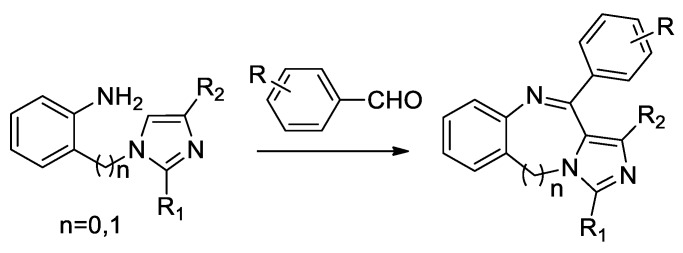
Sketched Aza-Pictet-Spengler.

In the same interval (2011–2015), the synthesis of scaffolds other than THIQ and THBC with potential biological and pharmaceutical application required the use of modified Pictet-Spengler reactions with structurally different substrates. For instance, in the oxa-Pictet-Spengler cyclization, that is an oxygen variation of the P-S reaction, an aromatic alcohol component, usually a β-arylethyl alcohol, reacts with a carbonyl compound (aldehyde, ketone, or their masked derivatives) to yield polysubstituted isochromans (Scheme 88) [303,304]. Recently a minireview on the catalytic enantioselective approaches to the oxa-P-S cyclization has been published by Zhu et al. [305]. On the other hand, arylamines linked to an activated aromatic nucleus, such as imidazole, might be treated with aldehydes to give imidazo-quinoxalines (*n* = 0) after DDQ oxidation [306] or triazabenzoazulenes (*n* = 1) [307] (Scheme 89). Several novel *N*-rich polycyclic skeletons have been synthesized by application of this P-S variation [308,309,310,311,312,313], for which we propose the name of aza-Pictet-Spengler. About these presences and more we may talk in the third Act. The curtain falls.

*No encore.*

*As the curtain falls,*

*All that is waiting is silence.*

(Spike Harper)

## Figures and Tables

**Table 1:** Pictet-Spengler reaction in total synthesis of natural products.

Target	Scheme/Figure	P-S Carbonyl Partner and *Catalyst*	Note	[Ref]
(±)-cribrostatin 4 (±)-renieramycin G	Scheme 20 Figure 3	(EtO)_2_CHCH_2_OBz *TMSOTf*	cytotoxic studies	[82,83,84,85]
(±)-hamayne	Figure 3	CH_2_O/HCO_2_H	crinine type alkaloids	[86]
(±)-lycorane	Figure 3	CH_2_O/HCl	*Ama*r*yllidaceae* family	[87]
(−)-saframycin A	Scheme 21	OHC-CH_2_NHCbz *CF_3_CH_2_OH*, *AcOH*, *4 Å MS*	intermolecular P-S (C-1) intramolecular P-S (C-11)	[88]
mitragynine, paynantheine, speciogynine	Scheme 22	Aldehyde **80** *Thiou*r*ea-de*r*ived catalysts*	*Mit*r*agyna* yohimbinoid alkaloids	[89]
(±)-tangutorine	Scheme 23	Aldehyde **83** *mic*R*owave*	the same cytotoxic activity for racemate and pure enantiomers	[90]
(−)-lemonomycin	Figure 3	Cinnamaldehyde *CSA*, *TMSCN*	potent activity against drug-resistant *cocci*	[91]
tetracyclic core of lemonomycin	Figure 3	OHC-CO_2_Et *CF_3_CH_2_OH*, *AcOH*, *4 Å MS*	substrate-induced stereocontrol strategy	[92]
venenatine alstovenine	Scheme 24	Compound **86** *HCl (aq) o*R *DMAP o*R *NaI*	yohimbinoid alkaloids C-3 stereochemistry	[93]
3-arylacrilamide (C-1)-side chain derivatives	Figure 3	OHC-CH_2_NHCbz *NaOAc/AcOH*	saframycin/ecteinascidin type compounds	[94,95]
(+)-lochnerine (+)-dispegatrine	Figure 3	OHC(CH_2_)_2_CO_2_Me *AcOH*	sarpagine type	[96]
(−)-jorunnamycins A, C (−)-jorumycin	Scheme 26	Compound **89** *CF_3_CH_2_OH*, *AcOH*	renieramycin type	[97]
(−)-renieramycin G	Scheme 26	Compound **89** *CF_3_CH_2_OH*, *AcOH*	renieramycin type	[98]
(±)-alstonerine	Scheme 25	Compound **91** *Wet CH_2_Cl_2_*	macroline/sarpagine type	[99]
erysotramidine	Scheme 25	Compound **93** *H_3_PO_4_*	erythrina alkaloids	[100,101]
(±)-actinophyllic acid	Scheme 27	Different form of P-S	indolohydroazocine	[102]
cribrostatin 4 renieramycin I	Scheme 28	(EtO)_2_CHCH_2_OBz *TMSOTf/Ac_2_O*	synthesis of left-half renieramycin model compound	[103,104]
(+)-yohimbine	Figure 3	Reported in [65]	-	[105]
(−)-corynantheidine	Figure 3	Reported in [65]	corynanthe alkaloids	[106]
(−)-corynantheine (−)-dihydrocorynantheine	Figure 3	Reported in [65]	corynanthe alkaloids	[107]
(−)-affinisine oxindole	Figure 3	Reported in [66]	-	[108]
